# Highly divergent satellitomes of two barley species of agronomic importance, *Hordeum chilense* and *H. vulgare*

**DOI:** 10.1007/s11103-024-01501-5

**Published:** 2024-10-02

**Authors:** Ana Gálvez-Galván, Lorena Barea, Manuel A. Garrido-Ramos, Pilar Prieto

**Affiliations:** 1 Plant Breeding Department, Institute for Sustainable Agriculture, Agencia Estatal Consejo Superior de Investigaciones Científicas (CSIC), Avda. Menéndez Pidal, Campus Alameda del Obispo s/n, 14004 Córdoba, Spain; 2 Area of Plant Breeding and Biotechnology, IFAPA Alameda del Obispo, Avda. Menéndez Pidal s/n, 14004 Córdoba, Spain; 3 Departamento de Genética, Facultad de Ciencias, Universidad de Granada, Avda. Fuentenueva s/n, 18071 Granada, Spain

**Keywords:** Satellite DNA, Satellitome, TEs, Centromeres, Subtelomeres, Chromosome recognition, Homologous pairing, Genome evolution, Cereal evolution, Fluorescence in situ hybridization (FISH), *Hordeum* evolution, *Hordeum vulgare*, *Hordeum chilense*

## Abstract

In this paper, we have performed an in-depth study of the complete set of the satellite DNA (satDNA) families (i.e. the satellitomes) in the genome of two barley species of agronomic value in a breeding framework, *H. chilense* (H1 and H7 accessions) and *H. vulgare* (H106 accession), which can be useful tools for studying chromosome associations during meiosis. The study has led to the analysis of a total of 18 satDNA families in *H. vulgare*, 25 satDNA families in *H. chilense* (accession H1) and 27 satDNA families in *H. chilense* (accession H7) that constitute 46 different satDNA families forming 36 homology groups. Our study highlights different important contributions of evolutionary and applied interests. Thus, both barley species show very divergent satDNA profiles, which could be partly explained by the differential effects of domestication versus wildlife. Divergence derives from the differential amplification of different common ancestral satellites and the emergence of new satellites in *H. chilense*, usually from pre-existing ones but also random sequences. There are also differences between the two *H. chilense* accessions, which support genetically distinct groups. The fluorescence in situ hybridization (FISH) patterns of some satDNAs yield distinctive genetic markers for the identification of specific *H. chilense* or *H. vulgare* chromosomes. Some of the satellites have peculiar structures or are related to transposable elements which provide information about their origin and expansion. Among these, we discuss the existence of different (peri)centromeric satellites that supply this region with some plasticity important for centromere evolution. These peri(centromeric) satDNAs and the set of subtelomeric satDNAs (a total of 38 different families) are analyzed in the framework of breeding as the high diversity found in the subtelomeric regions might support their putative implication in chromosome recognition and pairing during meiosis, a key point in the production of addition/substitution lines and hybrids.

**Supplementary Information:**

The online version contains supplementary material available at 10.1007/s11103-024-01501-5.

## Key message

Several satDNAs have been identified in the subtelomeres of wild and cultivated barley species that might contribute to the specificity of these distal chromosome regions during homologous recognition and pairing in meiosis.

**Supplementary Information:**

The online version contains supplementary material available at 10.1007/s11103-024-01501-5.

## Introduction

Cultivated barley, *Hordeum vulgare* ssp. *vulgare* L. (Baden and von Bothmer 1994; Bothmer et al. 1995) (2*n* = 2*x* = 14) is a member of the Poaceae family and one of the most important cereal crops in the world. Barley is a source of feed for livestock and of malt for brewing, with a production of more than 150 million tons (MT) in 2022, being the European Union the major grower with 65% of the global production (Faostat, https://www.fao.org/faostat/es/#data/QCL/visualize; consulted 04/03/2024). Cultivated barley was domesticated ~ 10,000 years ago from its progenitor *H. vulgare* subsp. *spontaneum* (K. Koch) Thell. (2*n* = 2*x* = 14) in the Fertile Crescent, a historically significant region for its early agricultural and human civilizations corresponding to part of the territories of the Mediterranean Levant and Mesopotamia (Brown et al. 2009), which is considered the primary centre of barley origin and domestication, although additional areas of domestication have been also proposed (Thormann et al. 2016; Blattner 2018). Understanding barley genome organization is essential to enable and facilitate the demand for better-adapted crops with higher yields, which must be implemented through breeding programs to ensure more efficient and sustainable production. Likewise, it is important to have information on the genomic organization of crop wild relatives which may provide new genes for crop improvement and adaptation. Such is the case, for example, of the wild barley species like *H. chilense* Roem. & Schult. (2*n* = 2*x* = 14), mainly distributed in Chile and Argentina (Blattner 2018), that contains desirable genes for wheat breeding (Forster et al. 1990; Martin et al. 1998; Rubiales et al. 2000; Martín et al. 2010; Calderón et al. 2012) or for the development of the wheat-barley amphiploids named tritordeum (Martín et al. 1999).

In the framework of breeding, it is important to elucidate how chromosomes associate and recombine during meiosis (the cellular process to generate the gametes in organisms with sexual reproduction), particularly in inter-specific genetic crosses, in which the success of recombination between chromosomes from different species is not high. In this context, subtelomeric sequences can be of particular importance during meiosis since they might be crucial for homologous chromosome recognition and association required for proper gamete segregation (Calderón et al. 2014; Naranjo 2015). We have previously found that the subtelomeric regions in barley and wheat are highly polymorphic and those polymorphisms could contribute to the specificity of the correct homologous pairing in both species (Aguilar and Prieto 2020, 2021; Serrano-León et al. 2023). Because tandemly repetitive DNA or satellite DNA (satDNA) is one of the main components of subtelomeric as well as (peri)centromeric regions (Garrido-Ramos 2015, 2017, 2021) we have recently proposed that satDNAs also contribute to the polymorphism that exists in the terminal region of the chromosomes (Gálvez-Galván et al. 2024).

The newly updated barley genome sequence assembly (*H. vulgare* subsp. *vulgare*, MorexV3; https://www.ncbi.nlm.nih.gov/datasets/genome/GCF_904849725.1/) has accelerated comparative genomics analyses of barley and other species (Mascher et al. 2021). Lamentably, it has been proven that known repeat arrays in telomeres, subtelomeres, centromeres, and 5S and 45S rDNA loci are not entirely represented in the current barley reference genome sequence assembly (Navrátilová et al. 2022; Serrano-León et al. 2023). Using a computational pipeline designed to identify and characterize repetitive DNA sequences in next-generation sequencing data (Novák et al. 2013, 2017; Ruiz-Ruano et al. 2016) we have screened Illumina sequencing data from *H. vulgare* subsp. *vulgare* and *H. chilense* H1 and H7 accessions to characterize the complete set of satDNA families (i.e., the satellitome) of both species and compared them with special emphasis on their genomic location and organization.

## Materials and Methods

### Plant material and growing conditions

Two different accessions (H1 and H7) from the wild barley *H. chilense* (Roem. et Schult.) (2*n* = 2*x* = 14; genome size =  ~ 5.3 Gb; (Bennett and Smith 1976), and the domestic barley *H. vulgare* L. cv. Betzes (accession H106) (2*n* = 2*x* = 14; genome size =  ~ 5 Gb; Doležel et al. 1998, 2018) were used in this work to perform both genomic and cytogenetic analyses. Seeds from the barley species were germinated and incubated in the dark at 4 °C on wet filter paper in Petri dishes for 4–5 days, then transferred to 25 °C for 1–2 days until germination. Roots were dissected and treated for accumulation of mitotic cells and then seeds were transferred to pots and grown in the greenhouse under semi-controlled conditions of temperature (25 °C day/15 °C night) and relative humidity (40%). After two weeks, the plant material was ready for genomic DNA isolation.

### Satellitome analysis

Genomic DNA (gDNA) was isolated from young frozen barley leaves using the standard CTAB procedure (Murray and Thompson 1980) with some modifications (Hernández et al. 2001). The DNA quality and concentration were determined using a NanoDrop1000 spectrophotometer (NanoDrop Technologies, USA).

Next Generation Sequencing was carried out at Macrogen Inc. (Macrogen Inc., Seoul, Korea) based on Illumina NovaSeq 6000 150PE (2 × 151 bp), yielding about 20.4 Gb (~ 4 × coverage), 20.7 Gb (~ 4 × coverage) and 20.3 Gb (~ 4 × coverage) data for *H. chilense* (accession H1), *H. chilense* (accession H7) and *Hordeum vulgare* (accession H106), respectively. Illumina sequencing raw data can be accessed at SRA-Genbank database in the BioProjects PRJNA1039805 and PRJNA1040438.

We applied the protocol satMiner (Ruiz-Ruano et al. 2016), based on consecutive rounds of clustering of Illumina reads by RepeatExplorer 2 (Novák et al. 2013, 2020), using a subset of reads (2,000,000 per library), and subsequent filtering of the already assembled reads using DeconSeq (Schmieder and Edwards 2011). RepeatExplorer 2 (Novák et al. 2013, 2020) executes an integrated version of the TAREAN tool (Novák et al. 2017), which performs automated identification of satellite DNA repeats based on the topology of their cluster graphs.

We first performed a quality trimming with Trimomatic (Bolger et al. 2014), and randomly selected 2 × 2,000,000 Illumina reads with SeqTK (https://github.com/lh3/seqtk), to run RepeatExplorer2 with default options. Cluster graphs with circular shapes were selected using TAREAN which generates a consensus monomer sequence for each satDNA cluster.

We filtered out the reads showing homology with the already clustered contigs and the already identified satDNA using DeconSeq, and selected a new set of 2 × 2,000,000 reads from the filtered libraries, that were clustered with RepeatExplorer2 in a second round. This allows detecting satDNAs being poorly represented in the raw reads. We repeated the filtering using the clusters in the second round and selected 2 × 2,000,000 reads for three additional rounds. Performing additional rounds of clustering and filtering have shown to be highly successful as it allows the detection of satDNAs which, due to their low abundance, had gone unnoticed because those of highly abundant elements masked their signals (Ruiz-Ruano et al. 2016).

To estimate abundance and divergence for each identified satDNA, we aligned 2 × 10 millions of randomly selected read pairs to the consensus sequences in the resulting satDNA database, using RepeatMasker with a publicly available script (https://github.com/fjruizruano/satminer/blob/master/repeat_masker_run_big.py). We used the calcDivergenceFromAlign.pl built-in tool of RepeatMasker to obtain a histogram of the Kimura two-parameter divergence for each element. Next, we transformed the abundance values to express them as genome proportions by dividing the number of aligned nucleotides by the total number of nucleotides in the selection of 20 million reads. The resulting histograms are referred to as Repeat Landscapes (RLs). We also used this procedure to search for each isolated satellite in each accession in the rest of the accessions analyzed by aligning 2 × 10 millions randomly selected read pairs from each barley accession to the consensus sequences in each accession-specific satDNA database.

We searched for homology between barley satellitomes with the rm_homolgy script (Ruiz-Ruano et al. 2016) that makes all-to-all alignments with Repeat-Masker v4.0.5 (Smit et al. 2015). In addition, we searched for homologies with transposable elements with RepeatMasker with “no_low” and “no_is” options.

The Adenine/Thymine content (AT%) was calculated using the bioinformatic tool “GC Content Calculator” (https://www.biologicscorp.com/tools/GCContent) developed by (Guerra et al. 2016).

A search of these satellite sequences in the barley genome was carried out using Basic Local Alignment Search Tool (BLAST®) trailing the genome assembly of *Hordeum vulgare* subsp. *vulgare* (https://www.ncbi.nlm.nih.gov/assembly/ GCF_904849725.1), through NCBI's Genome Data Viewer (GDW) to identify the locations of each satDNA family within the barley genome.

### Probes designed for cytogenetic validation of satDNA sequences by in situ hybridization

The different satDNAs families were amplified by PCR (“Polymerase Chain Reaction”) using specific primers designed with the Primer-BLAST software tool from NCBI (https://www.ncbi.nlm.nih.gov/tools/primer-blast/index.cgi?LINK_LOC=BlastHome) (Table S1). For monomers shorter than 80 bp, primers were designed manually. OligoAnalyzer™ tool (https://eu.idtdna.com/calc/analyzer) was used to confirm the absence of putative secondary structures in the sequences of the primers (hairpins, self-dimers and hetero-dimers). 20 ng of each *H. chilense* accession or *H. vulgare* (H106) genomic DNA were used to perform the PCR reaction with different polymerases according to the size of the sequence (MyTaqTM Plant-PCR Kit or MyFiTM DNA Polymerase, both from Bioline) (see Table S1). For families with sequences consisting of monomers between 80–1000 bp, we performed PCR amplification with the following conditions: a starting denaturation step at 94 °C for 5 min (minutes), 35 cycles at 94 °C for 30 s (seconds), followed by an annealing step of 42–60 °C (primer-dependent, see Table S1) for 30 s, and an extension at 72 °C during 1 min. A final extension step at 72ºC for 6 min was added. In the cases of amplifying satDNAs shorter than 80 bp, we reduced the time of annealing to 10 s to get longer amplicons according to (Ruiz-Ruano et al. 2016). PCR products from short monomers were displayed as a smear in agarose gels and were re-amplified using 1 μL of the previous PCR product in a new PCR mix. For satDNAs with a monomer sequence larger than 1 Kb, the extension time was modified, to find the optimal condition, around 45 s/Kb. FavorPrepTM Gel/PCR Purification Mini Kit (FAVORGEN) was used to extract the desired size band from the agarose gel. DNA amplification samples were sequenced to confirm the reliability of the PCR products.

PCR products were loaded in 1% agarose gel electrophoresis in 1 × TAE (40 mM TrisBase, 20 mM Acetate and 1 mM EDTA, dH2O until the volume of 1 L) running buffer and visualized using Quantity One 1-D Analysis Software Bio-Rad. According to the size of the sequence, a 100 bp or 1 Kb DNA Ladder ready to Load (Solis BioDyne) were used as a reference for molecular weight DNA.

The satDNAs families sequences from *H. chilense* and *H. vulgare* were labelled by nick translation with digoxigenin-11-dUTP (Roche Applied Science, Indianapolis, IN, USA) and with biotin-11-dUTP (Boehringer Mannheim Biochemicals, Germany), respectively, according to the manufacturer’s instructions. Nick translation was performed in a thermocycler (ThermoBrite® Leica) at 15 °C for 90 min.

### Chromosome preparations of root tip cells in somatic metaphase

Barley seeds were germinated on wet filter paper in the dark for 5 days at 4 °C, followed by 24 h at 25 °C. Emerging seedling roots 1–2 cm (centimetres) long were cut, incubated for 4 h in 0.05% w/v colchicine at 25 °C, fixed in 100% ethanol- acetic acid, 3:1 (v:v) and stored at 4 °C until their use.

Preparation of chromosome spreads was done as described in (Prieto et al. 2001; Prieto et al. 2004a) with some modifications. That is, before squashing, roots were washed in 1 × enzyme buffer (4 mM citric acid and 6 mM sodium citrate) 3 times for 5 min each. Then, meristems were cut and incubated for one hour in the enzyme mixture (0.5% pectolyase Y23 (Kyowa Chemical Products Co., LTD), 1% cellulose “Onozuka” RS (Yakult Pharmaceutical Ind. Co., LTD) and 20% peptinase (Sigma) in dH_2_O).

### Fluorescence in situ hybridization (FISH)

For in situ hybridization experiments, biotin and digoxigenin labelled probes were mixed to a final concentration of 5 ng/μl in the hybridization mixture (50% formamide, 2 × SCC, 5 ng of each digoxigenin and biotin-labelled probes, 10% dextran sulphate, 0.14 μg of yeast tRNA, 0.1 μg of sonicated salmon sperm DNA, and 5 ng of glycogen). The in situ hybridization protocol was performed according to (Cabrera et al. 2002). At least two slides per satDNA per *H. chilense* or *H. vulgare* lines were hybridized in FISH experiments.

Post-hybridization washes were conducted twice at 2 × SSC (5 min each) at 37 °C followed by another wash in 1 × SSC at room temperature (RT). Biotin- and digoxigenin-labelled probes were detected with streptavidin-Cy3 conjugates (Sigma, St. Louis, MO, USA) and antidigoxigenin FITC antibodies (Roche Diagnostics, Meylan, France), respectively. Total DNA was counterstained with 4′,6-diamidino-2- phenylindole (DAPI) and mounted in Vectashield (Vector Laboratories, Burlingame, CA, USA). Hybridization results were visualized using a Nikon Eclipse 80i epifluorescence microscope and images were captured with a Nikon CCD camera using the Nikon 3.0 software (Nikon Instruments Europe BV, Amstelveen, The Netherlands) and processed with Photoshop 11.0.2 software for adjustment of brightness and contrast (Adobe Systems Inc., San Jose, CA, USA).

Chromosomes from the wild and cultivated barley accessions displaying positive signals for the satDNAs sequences were identified using the repeat sequence GAA as described previously (Pedersen et al. 1996; Pedersen and Langridge 1997; Prieto et al., 2004a; Kruppa et al. 2013).

## Results

### Barley satellitome survey

SatDNA mining revealed 25 satDNA families in *H. chilense* (accession H1), representing ~ 4.3% of the genome (Table 1), 27 satDNA families in *H. chilense* (accession H7), representing ~ 3.6% of the genome (Table 2) and 18 satDNA families in *H. vulgare* representing ~ 2.1% of the genome (Table 3). Figures S1–S3 show the reconstruction of representative monomer sequences for each satDNA family. Intra-specific homologies between several of these satDNAs which form different superfamilies were found. For example, the superfamily SF3 in *H. chilense-*H1 is composed of three satDNA families (HchH1Sat03-118, HchH1Sat04-118 and HchH1Sat12-118). There are homologous satellites to these satDNAs in *H. chilense-*H7 (three families) and *H. vulgare-*H106 (three families) and the nine satDNA families comprise the Group number 3 (GR3) of satellite sequences (Tables 1, 2, 3).

**Table 1:** Metrics of different parameters of satDNAs identified in *Hordeum chilense*-H1

GR	SF	Satellite database	Lenght	Abundance	Variation	AT (%)	Homologies	Hch7 homologous	Hvu homologous	FISH
GR1		HchH1Sat01-337	337	3.0498%	0.1030	64		HchH7Sat01-337	HvuSat01-338	ST/I
GR2	SF2	HchH1Sat02-92	92	0.2960%	0.0747	33	HchH1Sat18-46	HchH7Sat06-46		I
GR3	SF3	HchH1Sat03-118	118	0.1678%	0.0735	45	HchH1Sat04-118; HchH1Sat12-118	HchH7Sat02-118; HchH7Sat05-118; HchH7Sat21-118	HvuSat03-118; HvuSat04-118; HvuSat07-118	ST
GR3	SF3	HchH1Sat04-118	118	0.1589%	0.1069	47	HchH1Sat03-118; HchH1Sat12-118	HchH7Sat02-118; HchH7Sat05-118; HchH7Sat21-118	HvuSat03-118; HvuSat04-118; HvuSat07-118	ST
GR4	SF4	HchH1Sat05-334	334	0.0930%	0.1081	36	HchH1Sat11-336; HchH1Sat21-332	HchH7Sat04-334; HchH7Sat08-336		ST/I
GR5	SF5	HchH1Sat06-352	352	0.0904%	0.0520	46	HchH1Sat13-344	HchH7Sat03-355; HchH7Sat09-344		ST
GR6		HchH1Sat07-493	493	0.0641%	0.2090	60		HchH7Sat07-484		C
GR7		HchH1Sat08-570	570	0.0593%	0.0772	52		HchH7Sat19-518		ST
GR8		HchH1Sat09-1236	1236	0.0518%	0.1045	60		HchH7Sat12-728		D
GR9		HchH1Sat10-652	652	0.0491%	0.1758	57		HchH7Sat10-662		ST
GR4	SF4	HchH1Sat11-336	336	0.0489%	0.1152	38	HchH1Sat05-334; HchH1Sat21-332	HchH7Sat04-334; HchH7Sat08-336		ST/I
GR3	SF3	HchH1Sat12-118	118	0.0285%	0.1166	42	HchH1Sat03-118; HchH1Sat04-118	HchH7Sat02-118; HchH7Sat05-118; HchH7Sat21-118	HvuSat03-118; HvuSat04-118; HvuSat07-118	ST
GR5	SF5	HchH1Sat13-344	344	0.0260%	0.1116	44	HchH1Sat06-352	HchH7Sat03-355; HchH7Sat09-344		I
GR10	SF10	HchH1Sat14-88	88	0.0247%	0.0733	71	HchH1Sat25-44	HchH7Sat15-44		I
GR11	SF11	HchH1Sat15-503	503	0.0212%	0.0758	46	HchH1Sat24-1932	HchH7Sat16-503		ST
GR12	SF12	HchH1Sat16-320	320	0.0202%	0.0790	52	HchH1Sat17-320	HchH7Sat13-320; HchH7Sat20-320	HvuSat13-319	ST
GR12	SF12	HchH1Sat17-320	320	0.0117%	0.1435	55	HchH1Sat16-320	HchH7Sat13-320; HchH7Sat20-320	HvuSat13-319	ST
GR2	SF2	HchH1Sat18-46	46	0.0098%	0.0578	33	HchH1Sat02-92	HchH7Sat06-46		I
GR13		HchH1Sat19-917	917	0.0091%	0.0410	55		HchH7Sat17-1262		I
GR14		HchH1Sat20-245	245	0.0086%	0.0541	58		HchH7Sat24-245		I
GR4	SF4	HchH1Sat21-332	332	0.0065%	0.1915	45	HchH1Sat05-334; HchH1Sat11-336			ST
GR15		HchH1Sat22-1044	1044	0.0043%	0.0618	54				ST
GR16		HchH1Sat23-4077	4077	0.0040%	0.0302	55				D
GR11	SF11	HchH1Sat24-1932	1932	0.0040%	0.0983	50	HchH1Sat15-503			–-
GR10	SF10	HchH1Sat25-44	44	0.0001%	0.0441	71	HchH1Sat14-88	HchH7Sat15-44		I
				4.3078%						

Group (GR), superfamilies (SF), satellite database, length (nt), abundance (% of the genome), variation, A + T content (%), homologies in *H. chilense*-H1 (HchH1), satDNAs homologous in *H. chilense-*H7 (HchH7), satDNAs homologous in *Hordeum vulgare*-H106 (Hvu) and FISH pattern. FISH: dispersed (satDNAs with scattered signal along the whole chromosome); (peri)centromeric (satDNAs with positive signal around the centromere of the chromosomes); terminal (satDNAs with signal located in the terminal regions of the chromosomes (subtelomeres); interstitial (satDNAs with interstitial signal in chromosome arms)

**Table 2:** Metrics of different parameters of satDNAs identified in *Hordeum chilense*-H7

GR	SF	Satellite database	Lenght	Abundance	Variation	AT (%)	Homologies	Hch1 homologous	Hvu homologous	FISH
GR1		HchH7Sat01-337	337	2.3856%	0.0892	63		HchH1Sat01-337	HvuSat01-338	ST/I
GR3	SF3	HchH7Sat02-118	118	0.1811%	0.0951	48	HchH7Sat05-118; HchH7Sat21-118	HchH1Sat03-118; HchH1Sat04-118; HchH1Sat12-118	HvuSat03-118; HvuSat04-118; HvuSat07-118	ST
GR5	SF5	HchH7Sat03-355	355	0.1397%	0.0529	45	HchH7Sat09-344	HchH1Sat06-352; HchH1Sat13-344		ST/I
GR4	SF4	HchH7Sat04-334	334	0.1386%	0.0931	35	HchH7Sat08-336	HchH1Sat05-334; HchH1Sat11-336; HchH1Sat21-332		ST/I
GR3	SF3	HchH7Sat05-118	118	0.1224%	0.0893	47	HchH7Sat02-118; HchH7Sat21-118	HchH1Sat03-118; HchH1Sat04-118; HchH1Sat12-118		ST
GR2		HchH7Sat06-46	46	0.1102%	0.0584	33		HchH1Sat02-92; HchH1Sat18-46		ST/I/C
GR6		HchH7Sat07-484	484	0.0581%	0.2157	61		HchH1Sat07-493		C
GR4	SF4	HchH7Sat08-336	336	0.0551%	0.1083	37	HchH7Sat04-334	HchH1Sat05-334; HchH1Sat11-336; HchH1Sat21-332		ST/I
GR5	SF5	HchH7Sat09-344	344	0.0496%	0.0744	44	HchH7Sat03-355	HchH1Sat06-352; HchH1Sat13-344		I
GR9		HchH7Sat10-662	662	0.0480%	0.1669	58		HchH1Sat10-652		ST/I
GR17		HchH7Sat11-505	505	0.0417%	0.1137	62				C
GR8		HchH7Sat12-728	728	0.0371%	0.1326	57		HchH1Sat09-1236		D
GR12	SF12	HchH7Sat13-320	320	0.0361%	0.0915	52	HchH7Sat20-320	HchH1Sat16-320; HchH1Sat17-320	HvuSat13-319	ST
GR18		HchH7Sat14-2790	2790	0.0313%	0.0737	53				C
GR10		HchH7Sat15-44	44	0.0310%	0.0621	71		HchH1Sat14-88; HchH1Sat25-44		I
GR11		HchH7Sat16-503	503	0.0302%	0.0667	46		HchH1Sat15-503; HchH1Sat24-1932		ST
GR13		HchH7Sat17-1262	1262	0.0300%	0.1487	50		HchH1Sat19-917		I
GR19		HchH7Sat18-46	46	0.0247%	0.0659	28				I
GR7		HchH7Sat19-518	518	0.0220%	0.0892	52		HchH1Sat08-570		ST
GR12	SF12	HchH7Sat20-320	320	0.0166%	0.1085	55	HchH7Sat13-320	HchH1Sat16-320; HchH1Sat17-320	HvuSat13-319	I
GR3	SF3	HchH7Sat21-118	118	0.0163%	0.1747	43	HchH7Sat02-118; HchH7Sat05-118	HchH1Sat03-118; HchH1Sat04-118; HchH1Sat12-118		ST
GR20		HchH7Sat22-364	364	0.0107%	0.0430	53				ST
GR21		HchH7Sat23-692	692	0.0062%	0.0509	53				D
GR14		HchH7Sat24-245	245	0.0042%	0.0690	58		HchH1Sat20-245		I
GR22		HchH7Sat25-82	82	0.0037%	0.1049	68				–-
GR23		HchH7Sat26-4341	4341	0.0030%	0.0190	59				ST
GR24		HchH7Sat27-410	410	0.0013%	0.0855	48				–-
				3.6342%						

Group (GR), superfamilies (SF), satellite database, length (nt), abundance (% of the genome), variation, A + T content (%), homologies in *H. chilense*-H7 (HchH7), satDNAs homologous un *H. chilense-*H1 (HchH1), satDNAs homologous in *H. vulgare*-H106 (Hvu) and FISH pattern. FISH: dispersed (satDNAs with scattered signal along the whole chromosome); (peri)centromeric (satDNAs with positive signal around the centromere of the chromosomes); terminal (satDNAs with signal located in the terminal regions of the chromosomes (subtelomeres); interstitial (satDNAs with interstitial signal in chromosome arms)

**Table 3:** Metrics of different parameters of satDNAs identified in *Hordeum vulgare*-H106

GR	SF	Satellite database	Lenght	Abundance	Variation	AT (%)	Homologies	HchH1 homologous	HchH7 homologous	FISH
GR1		HvuSat01-338	338	0.6031%	0.0758	65		HchH1Sat01-337	HchH7Sat01-337	ST/I
GR25		HvuSat02-444	444	0.5547%	0.1211	59				ML
GR3	SF3	HvuSat03-118	118	0.3694%	0.0808	47	HvuSat04-118; HvuSat07-118	HchH1Sat03-118; HchH1Sat04-118; HchH1Sat12-118	HchH7Sat02-118; HchH7Sat05-118; HchH7Sat21-118	ST
GR3	SF3	HvuSat04-118	118	0.1529%	0.2050	49	HvuSat03-118; HvuSat07-118	HchH1Sat03-118; HchH1Sat04-118; HchH1Sat12-118	HchH7Sat02-118; HchH7Sat05-118; HchH7Sat21-118	ST
GR26		HvuSat05-5500	5500	0.1175%	0.2235	65				D
GR27		HvuSat06-4925	4925	0.0647%	0.3377	56				D
GR3	SF3	HvuSat07-118	118	0.0562%	0.1493	48	HvuSat03-118; HvuSat07-118	HchH1Sat03-118; HchH1Sat04-118; HchH1Sat12-118	HchH7Sat02-118; HchH7Sat05-118; HchH7Sat21-118	ST
GR28		HvuSat08-263	263	0.0462%	0.0648	52				ST
GR29	SF29	HvuSat09-900	900	0.0218%	0.0727	66	HvuSat12-541			ST
GR30		HvuSat10-5985	5985	0.0167%	0.1852	59				ST
GR31		HvuSat11-366	366	0.0136%	0.1582	61				ST/I
GR29	SF29	HvuSat12-541	541	0.0111%	0.1318	68	HvuSat09-900			ST
GR12		HvuSat13-319	319	0.0095%	0.1010	56		HchH1Sat16-320; HchH1Sat17-320	HchH7Sat13-320; HchH7Sat20-320	I
GR32		HvuSat14-2330	2330	0.0093%	0.0066	55				I
GR33		HvuSat15-1590	1590	0.0081%	0.1721	51				C
GR34		HvuSat16-483	483	0.0075%	0.0436	47				–-
GR35		HvuSat17-632	632	0.0039%	0.0876	64				I
GR36		HvuSat18-2988	2988	0.0038%	0.1097	60				ST
				2.0700%						

Group (GR), superfamilies (SF), satellite database, length (nt), abundance (% of the genome), variation, A + T content (%), homologies in *H. chilense*-H7(HchH7), satDNAs homologous un *H. chilense-*H1 (HchH1), satDNAs homologous in *H. vulgare*-H106 (Hvu) and FISH pattern. FISH: dispersed (satDNAs with scattered signal along the whole chromosome); (peri)centromeric (satDNAs with positive signal around the centromere of the chromosomes); terminal (satDNAs with signal located in the terminal regions of the chromosomes (subtelomeres); interstitial (satDNAs with interstitial signal in chromosome arms)

**Fig. 1:**
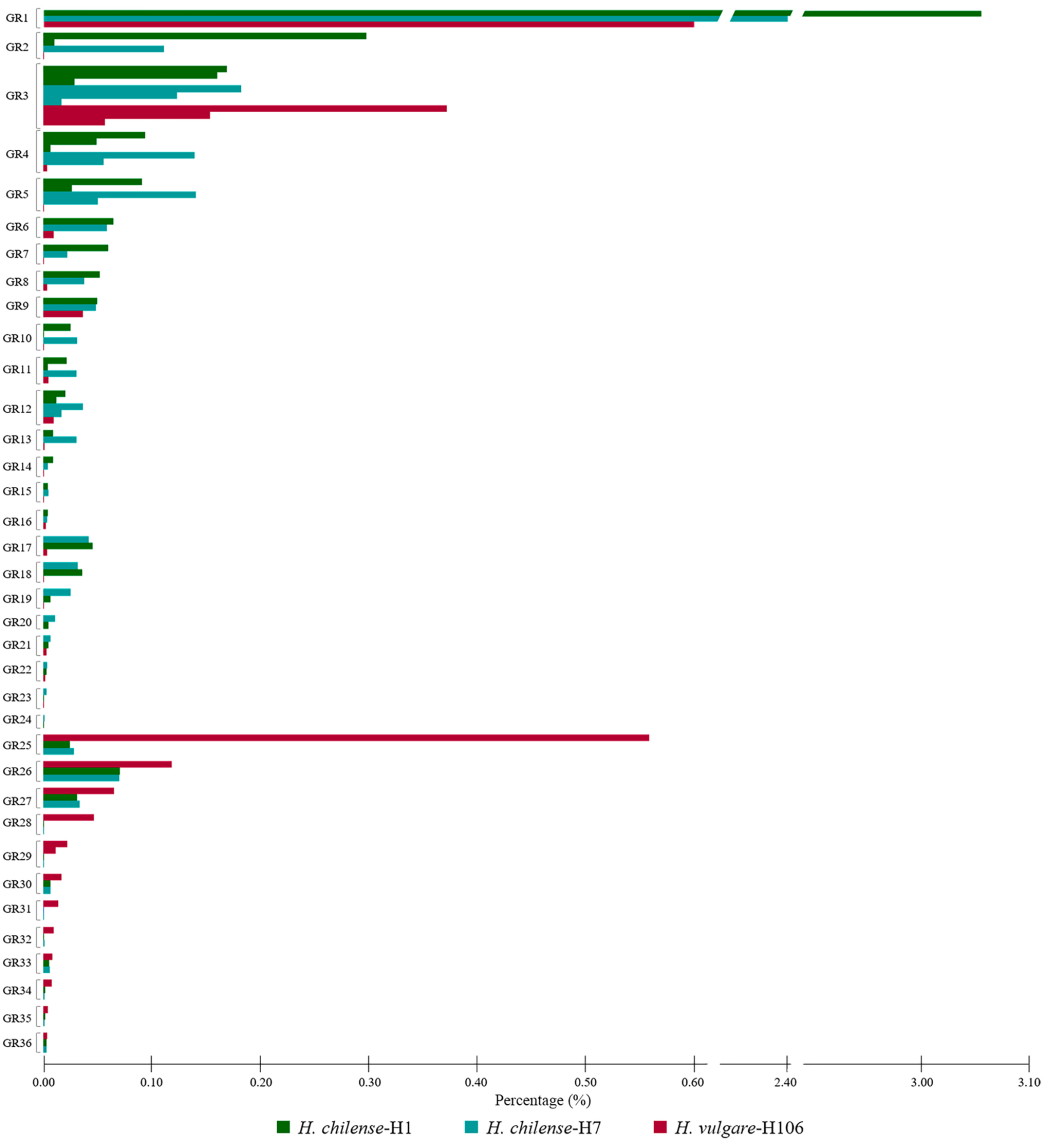
Diagram displaying the abundance of each satDNA family group (GR). Bars represent the abundance (%) of each satDNA family per species (Dark green: *H. chilense*-H1; Light green: *H. chilense*-H7; Red: *H. vulgare*-H106)

It is striking at first sight that very few satellites of *H. chilense* would have homologues in *H. vulgare* and vice versa (Tables 1–3). Thus, in principle, the two species would share only 5 satDNA families. In addition, certain differences between accessions H1 and H7 of *H. chilense* have been found, that is 2 satellites in accession H1 not found in H7 and 8 satellites in H7 not found in accession H1. It is also striking that in the wild varieties of barley, the proportion of satellite DNA in the genome is about twice that in cultivated barley (about 2%) and, in addition, there are also considerable differences in this proportion between *H. chilense* accessions (about 4.3% in H1 and 3.6% in H7). To search for the presence of the presumed accession-specific satDNAs in each barley accession, we aligned 2 × 10 millions of randomly selected read pairs from each barley accession to the consensus sequences in the accession-specific satDNA database, using RepeatMasker as indicated in Materials and Methods. As a result, all thirteen alleged *H. vulgare*-specific satellites were evident in both *H. chilense* H1 and H7 accessions (Table 4). However, in most cases, the proportion of these satellites in H1 and H7 was very low (below 0.01% in 10 of 13 satellites and between 0.02% and 0.07% in the remaining three). Similarly, among the twenty-one alleged *H. chilense*-specific satDNAs, all but two were found in *H. vulgare* (Table 4). In this case, one satDNA was found to represent 0.036% of the *H. vulgare* genome but the remaining 18 were represented in a percentage below 0.01% (in fact, 9 satellites were under 0.001%). Table 4 shows the relationships of all these satDNA families. Collectively, they are grouped in 36 homology groups and 8 superfamilies, summing a total of 46 different satDNA families. According to Table 4, all indications are that 44 of these satellites were already present in the common ancestor of these barley species. Most of the groups are constituted by satDNA families that have been somewhat more amplified either in *H. chilense* (GR2, GR4, GR5, GR6, GR7, GR8, GR10, GR11, GR13, GR14, GR15, GR17, GR18, GR19, GR20, GR21 and GR24) or in *H. vulgare* (GR28, GR29, GR30, GR31, GR32, GR34 and GR35), with traces of each of them remaining in the other species (Table 4; Fig. 1). Conversely, amplifications were significant in the three accessions analyzed in seven groups of satDNAs, although differently among them (GR1, GR3, GR9, GR12, GR25, GR26 and GR27; a total of 10 different families). In addition, within *H. chilense*, we can also distinguish between accessions H1 and H7 since most shared satDNAs are more abundant in the H1 accession. Furthermore, 4 satDNA families are exclusive of this accession (Tables 2, 3 and 4): (a) HchH1Sat02-92 (GR2 and SF2) is composed of two 46-bp subunits and it is almost replacing the 46-bp satellite (HchH1Sat18-46) in H1 (0.30% *vs* 0.01%), which is present in the H7 accession (HchH7Sat06-46) in a smaller proportion (0,11%); (b) HchH1Sat21-332 is a new satDNA of the GR4 group and SF4 superfamily that is not present in H7; (c) In the GR10, HchH1Sat14-88 (0.02%) is composed of two 44-bp subunits and it has almost replaced in H1 the 44-bp satellite present in the H7 accession (HchH7Sat15-44) in a similar proportion (0.03%) while in the H1 accession (HchH1Sat25-44) it has almost disappeared (0.0001%); (d) HchH1Sat24-1932 is a newly emergent and still poorly represented satDNA included in GR11 group. Among the 10 satellite families with significant amplifications in the two species, it is also worth noting that some are more abundant in *H. chilense* and others in *H. vulgare*. Of note are the satellites included in GR1 group, which represent the higher proportion of the satellitome in H1 (71% of the satellitome) and H7 (66% of the satellitome) accessions of *H. chilense*. HvuSat01-338 (GR1) is also the most abundant satDNA in *H. vulgare* but represents 0.6% of the genome and it is four times less abundant than the representative satellites (HchH1Sat01-337 and HchH7Sat01-337) in *H. chilense* accessions (3.05% and 2.4% respectively). On the contrary, HvuSat03-118 (GR3 and SF3), is 0.37% of the *H. vulgare* genome while the respective counterparts in *H. chilense* H1 and H7 (HchH1Sat03-118 and HchH7Sat02-118) represent 0.17% and 0.18%, respectively, of their genomes. Also notable is the case of HvuSat02-444 (GR25) which is 0.55% of the *H. vulgare* genome but only 0.02%-0.03% in *H. chilense* (we couldn’t isolate these counterparts). Interestingly, this satellite consists of a cassette-like structure in which a 174 bp sequence is flanked by imperfect inverted repeats (130/139 bp) of the sequence (GAA)n [(TTC)n-174 bp-(GAA)n], noting that several of the TTC and GAA subrepeats of the (TTC)n and (GAA)n regions are degenerate (Figure S4). Repeat Landscape (i.e. the histogram representing abundance (y-axis) and divergence (x-axis) concerning a satDNA consensus sequence; see Materials and Methods) plots of each of the two parts of this satellite can be seen in Figure S5.

**Table 4:**
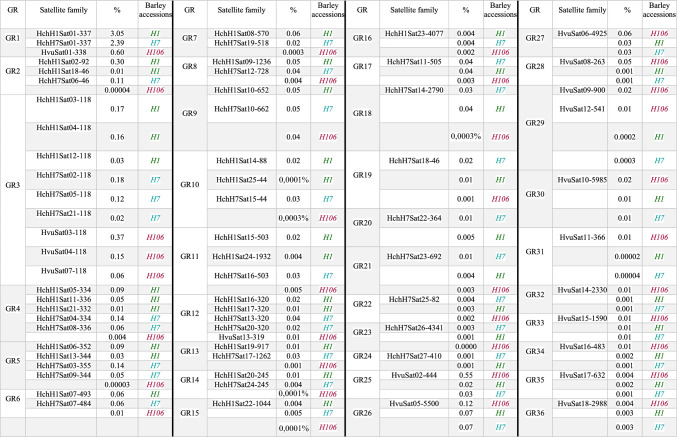
*Hordeum* satDNAs are organized by homology groups in which the percentage of each satellite in each genome analyzed is highlighted

In the comparison between *H. chilense* and *H. vulgare*, some specificity based on the RE results and the proportion that they represent in each genome can be highlighted, although trace copies of most satellites in each other species have been also detected. Thus, we can consider that there are 19 groups of sequences including 25 different satDNA families that are more characteristic of *H. chilense* (GR2, GR4, GR5, GR6, GR7, GR8, GR10, GR11, GR13, GR14, GR15, GR16, GR17, GR18, GR19, GR20, GR21, GR23 and GR24) while there are 6 groups (7 different satDNA families) more characteristic of *H. vulgare* (GR25, GR28, GR29, GR31, GR32 and GR34) (Table 4 and Fig. 1).

### satDNA homologies

BLAST search found significant similarity between several barley satDNA families (especially among those belonging to the groups GR1 and GR3) and other barley satDNAs previously reported (Table S2). In addition, these satellites, as well as other barley satDNAs, were homologous to other satDNAs previously described in other grass species (Table S2). Specifically, the GR1 satDNAs showed homology to the known Afa family found in *Hordeum* and several species of Poaceae while GR3 satDNAs are 118-bp subtelomeric satellites (see below) homologous to other satDNAs described under different designations in different grasses (Table S2). In addition, *H. chilense* satellites included in groups GR4, GR5, GR7, GR9, GR10 and GR12, were homologous to satDNAs sequences found in other Poaceae but not in *Hordeum* (Table S2).

An analysis using RepeatMasker revealed that 14 satDNA groups identified in this article as satDNAs showed homology with transposable elements, particularly DNA/CMC-EnSpm/CACTA elements although some of them are related to LTR retrotransposons (Table S3). Part of the sequence (between 20 and 100%, depending on the family) of six different satDNA families of *H. chilense* H1 showed homology to DNA/CMC-EnSpm/CACTA transposons. All these sequences were subtelomeric/interstitial except HchH1Sat09-1236 which showed a dispersed pattern (Table S3; see below). Part of the sequence (between 8 and 100%, depending on the family) of seven different satDNA families of *H. chilense* accession H7 also showed homology to DNA/CMC-EnSpm/CACTA transposons. All these sequences were subtelomeric/interstitial except HchH7Sat12-728 and HchH7Sat23-692, which were dispersed (Table S3; see below). Additionally, 25% of the (peri)centromeric HchH7Sat14-2790 satDNA sequence (see below), was homologous to LTR/Gypsy retrotransposons and 4.5% of the subtelomeric HchH7Sat26-satDNA sequence was homologous to DNA/hAT transposons. Two satDNA sequences of *H. vulgare*, HvuSat01-338 and HvuSat02-444, were related to DNA/CMC-EnSpm/CACTA transposons (95%-100% of their sequences; Table S3). However, another five satDNAs were homologous to LTR retrotransposons (between 12 and 89% of the sequence), including the (peri)centromeric HvuSat15-1590 satDNA (53% of its sequence is homologous to LTR/Copia elements) (Table S3).

### Satellite DNA location

**Fig. 2:**
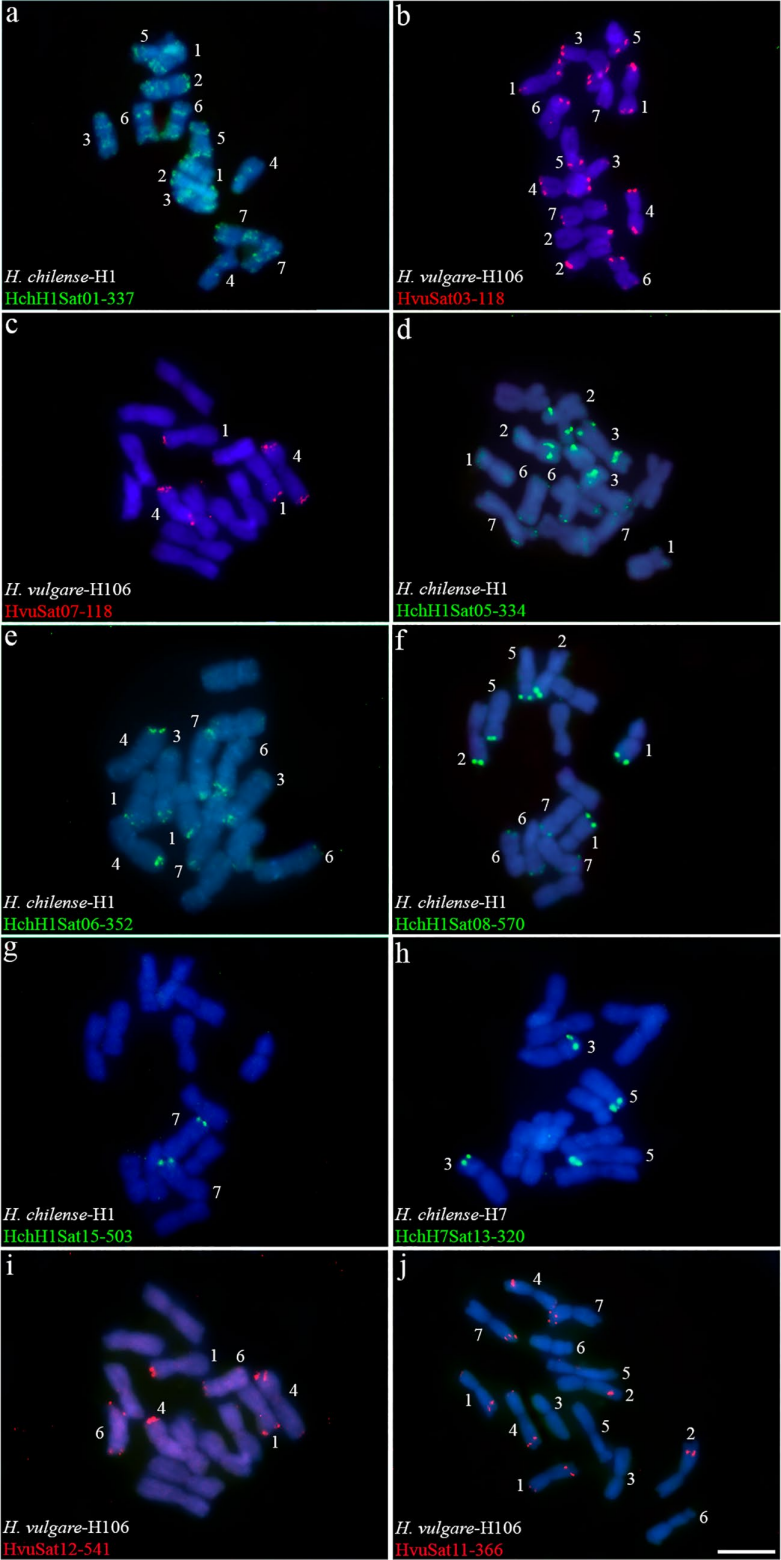
Cytogenetic visualization by fluorescence in situ hybridization of distal/subtelomeric satDNAs in metaphase chromosomes from *Hordeum vulgare* (H106) and *Hordeum chilense* (H1 and H7 accessions). DNA was counterstained with DAPI (blue). SatDNAs were indistinctly visualized in red or green using streptavidin-Cy3 and antidigoxigenin-FITC, respectively. **a** HchH1Sat01-337, **b** HvuSat03-118, **c** HvuSat07-118, **d** HchH1Sat05-334, **e** HchH1Sat06-352, **f** HchH1Sat08-570, **g** HchH1Sat15-503, **h** HchH7Sat13-320, **i** HvuSat12-541 and **j** HvuSat11-366. Scale bar = 10 µm

Most of the satellites analyzed, 38 in total, have a subtelomeric location (satellites included in groups GR1, GR2, GR3, GR4, GR5, GR7, GR9, GR11, GR12, GR15, GR20, GR23, GR28, GR29, GR30, GR31 and GR36) ranging from situations in which their presence is in a single chromosome pair to those in which their presence is in all seven chromosome pairs, passing through intermediate situations (Fig. 2; Table 5). It should be noted that some of these groups are the biggest ones (such as GR3 with 9 satellites). Some of these subtelomeric satellites (included in groups GR1, GR2, GR4, GR5, GR9 and GR31) displayed additional interstitial loci on some chromosomes (Fig. 2). In the cases of the GR5 and GR12 groups, composed mostly of subtelomeric satellites four satDNAs are interstitial, located at a single locus (Table 5).

**Table 5:**
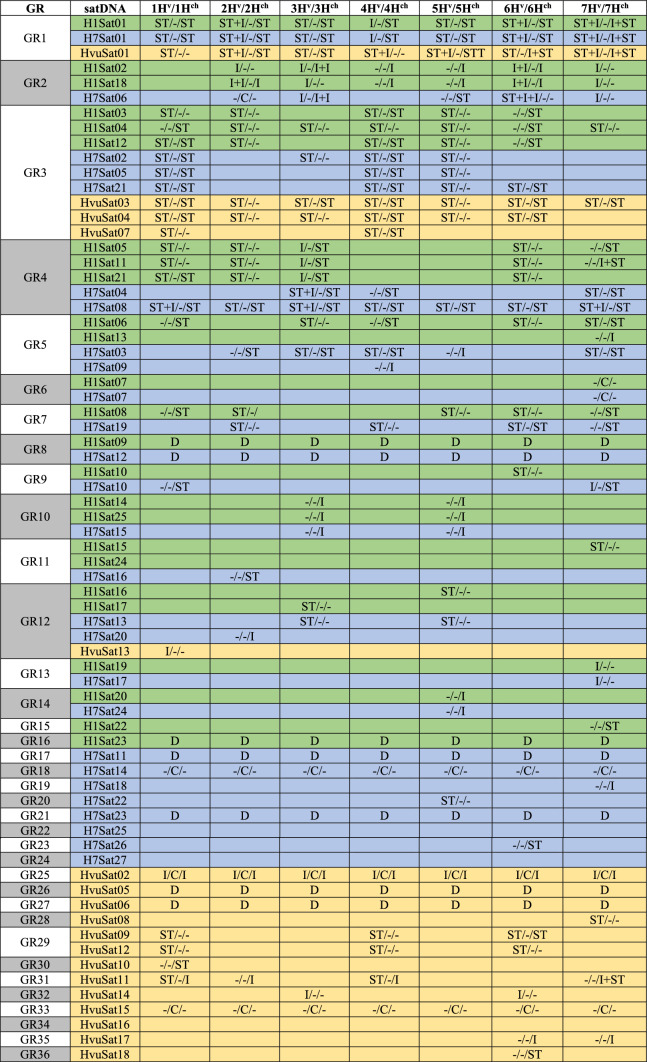
Summary of the fluorescence in situ hybridization (FISH) patterns for the different species: wild barley (*Hordeum chilense*-H1; H7) and cultivated barley (*Hordeum vulgare*-H106) satDNAs families identified in this work

The different lines collect the result to individual satDNA. Each column collects information on particular chromosomes. The different species are differentiated by colour (H1 = green; H7 = blue; H106 = orange). Each cell represents “Short arm/Centromere/Long arm”; ST = subtelomere; I = interstitial; C = (peri)centromeric; D = dispersed

**Fig. 3:**
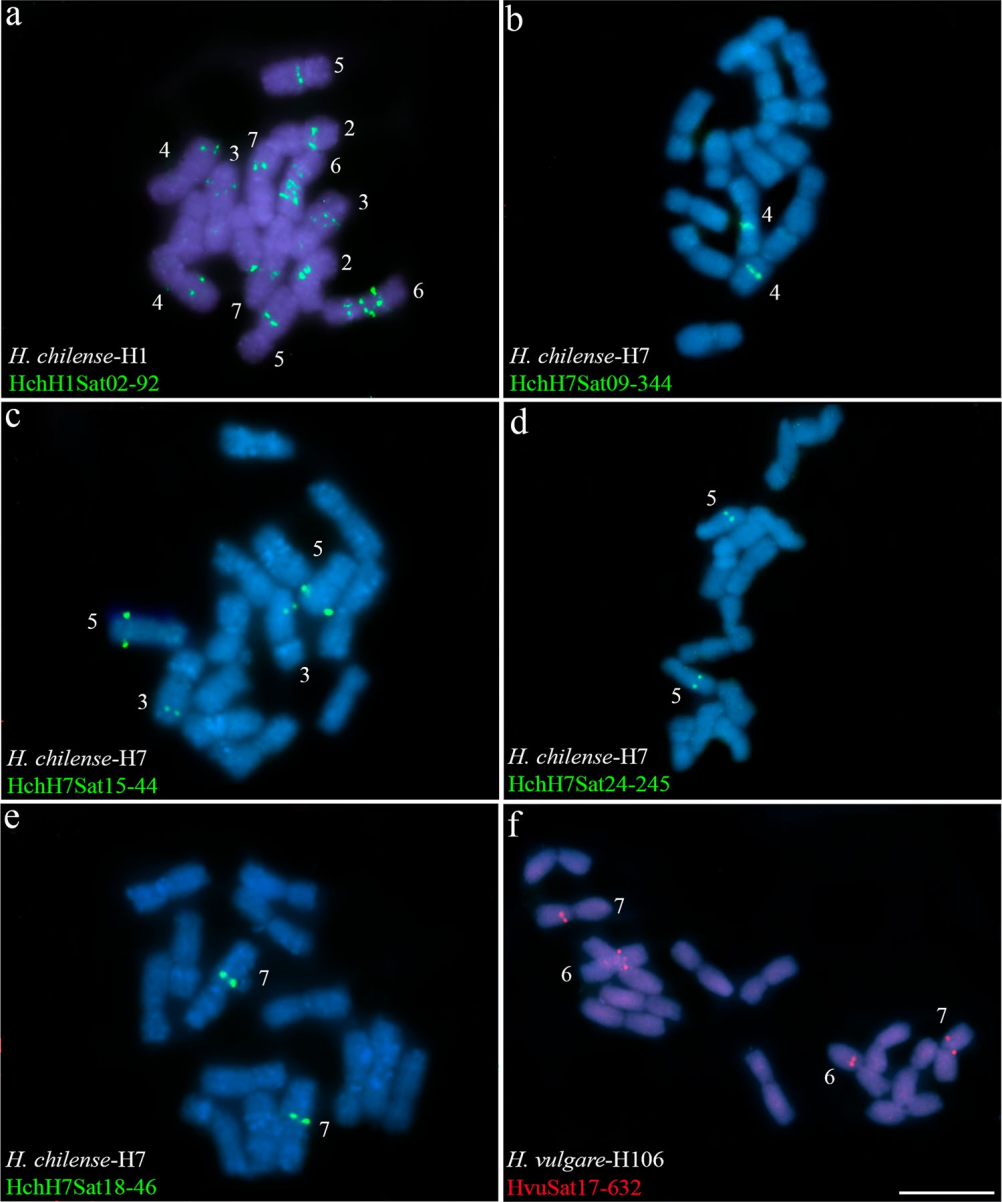
Cytogenetic visualization by fluorescence in situ hybridization of satDNAs displaying an interstitial pattern in metaphase chromosomes from *Hordeum vulgare* (H106) and *Hordeum chilense* (H1 and H7 accessions). DNA was counterstained with DAPI (blue). SatDNAs from *H. chilense* and *H. vulgare* were visualized in green and red using antidigoxigenin-FITC and streptavidin-Cy3, respectively. **a** HchH1Sat02-92, **b** HchH7Sat09-344, **c** HchH7Sat15-44, **d** HchH7Sat24-245, **e** HchH7Sat18-46 and **f** HvuSat17-632. Scale bar = 10 µm

A few satellites (satellites of groups GR2, GR10, GR13, GR14, GR19, GR32 and GR35), 16 in total, are located interstitially on one or a few pairs of chromosomes (Fig. 3 and Table 5). Among the satellites of GR2, HchH1Sat02-92 (Fig. 3a) and HchH7Sat18-46 are interstitial (Fig. 3e) in chromosomes 2–7, while satellite HchH7Sat06-46 occupies subtelomeric (2) loci in chromosomes 5 and 6, interstitial (3) loci in chromosomes 3, 6 and 7, and centromeric (1) loci in chromosome 2 (Table 5). This group of subtelomeric satellites would also include the four aforementioned satellites from the GR5 and GR12 groups.

**Fig. 4:**
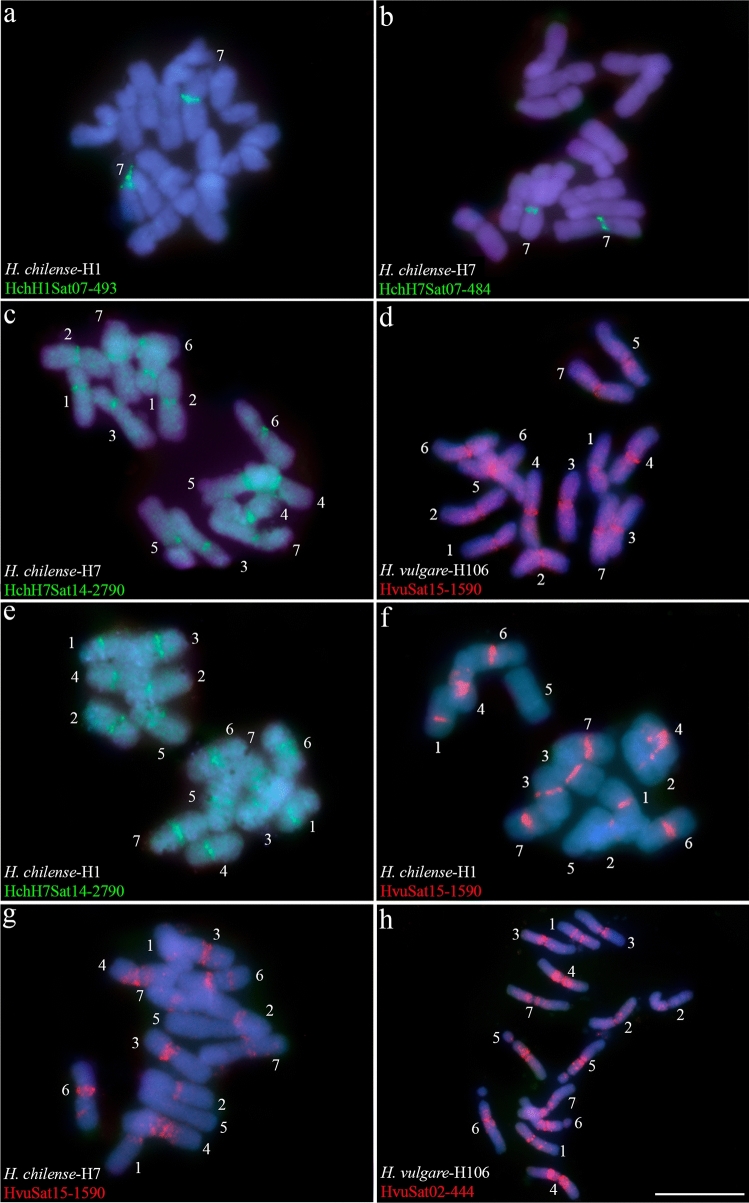
Cytogenetic visualization by fluorescence in situ hybridization of centromeric satDNAs in metaphase chromosomes from *Hordeum chilense* (H1 and H7 accessions) and *Hordeum vulgare* (H106). DNA was counterstained with DAPI (blue). SatDNAs from *H. chilense* and *H. vulgare* were visualized in green and red using antidigoxigenin-FITC and streptavidin-Cy3, respectively. **a** HchH1Sat07-493, **b** HchH7Sat07-484, **c** HchH7Sat14-2790, d) HvuSat15-1590. Panels **e**–**f** show cross-hybridization of *H. chilense* H7 and *H. vulgare* (H106) satellites in *H. chilense* (H1 and H7 accessions) chromosomes. **e** HchH7Sat14-2790 in *H. chilense*-H1, **f** HvuSat15-1590 in *H. chilense*-H1 and, **g** HvuSat15-1590 in *H. chilense*-H7. Panel **h** HvuSat02-444 with a related pattern to the GAA satellite. Scale bar = 10 µm

Four Satellite DNAs are identified as (peri)centromeric (Fig. 4 and Table 5). The homologs HchH1Sat07-493 and HchH7Sat07-484 (GR6), are present on one chromosomal pair (pair 7) of both *H. chilense* accessions H1 and H7 (Fig. 4a, b). In situ hybridization of these satellites in *H. vulgare* did not reveal FISH signals. In situ hybridization experiments also revealed that the (peri)centromeric satellite HchH7Sat14-2790 (GR18), present in all chromosomes of *H. chilense* H7 (Fig. 4c) was also detected in *H. chilense* H1 (Fig. 4e), while in *H. vulgare* signals were not detected in any chromosome (data not shown). In *H. vulgare*, the satellite HvuSat15-1590 (GR33) is (peri)centromeric in all chromosomes (Fig. 4d). Although we were unable to isolate a homologous satellite in *H. chilense*, in situ hybridization with the HvuSat15-1590 satellite in *H. chilense* accessions revealed its presence in the peri(centromeric) region of all chromosomes except in chromosome 5 in both *H. chilense* accessions, confirming that this satellite is conserved in both *H. vulgare* and *H. chilense* species (Fig. 4f, g).

In addition to these satDNAs, HvuSat02-444 (GR25) is a satellite that has multiple locations along the chromosomes and resembles the hybridization pattern of the GAA sequence (Dennis et al. 1980; Pedersen et al. 1996) although the pattern is more diffuse and a multitude of additional loci were observed scattered throughout the chromosomes of *H. vulgare* (Fig. 4h; Table 5).

Few satellites showed a dispersed pattern (HchH1Sat09-1236, HchH1Sat23-4077, HchH7Sat11-505, HchH7Sat12-728, HchH7Sat23-692, HvuSat05-5500 and HvuSat06-4925) or were not visualized by in situ hybridization (HchH1Sat24-1932, HchH7Sat25-82, HchH7Sat27-410 and HvuSat16-483) (data not shown).

**Fig. 5:**
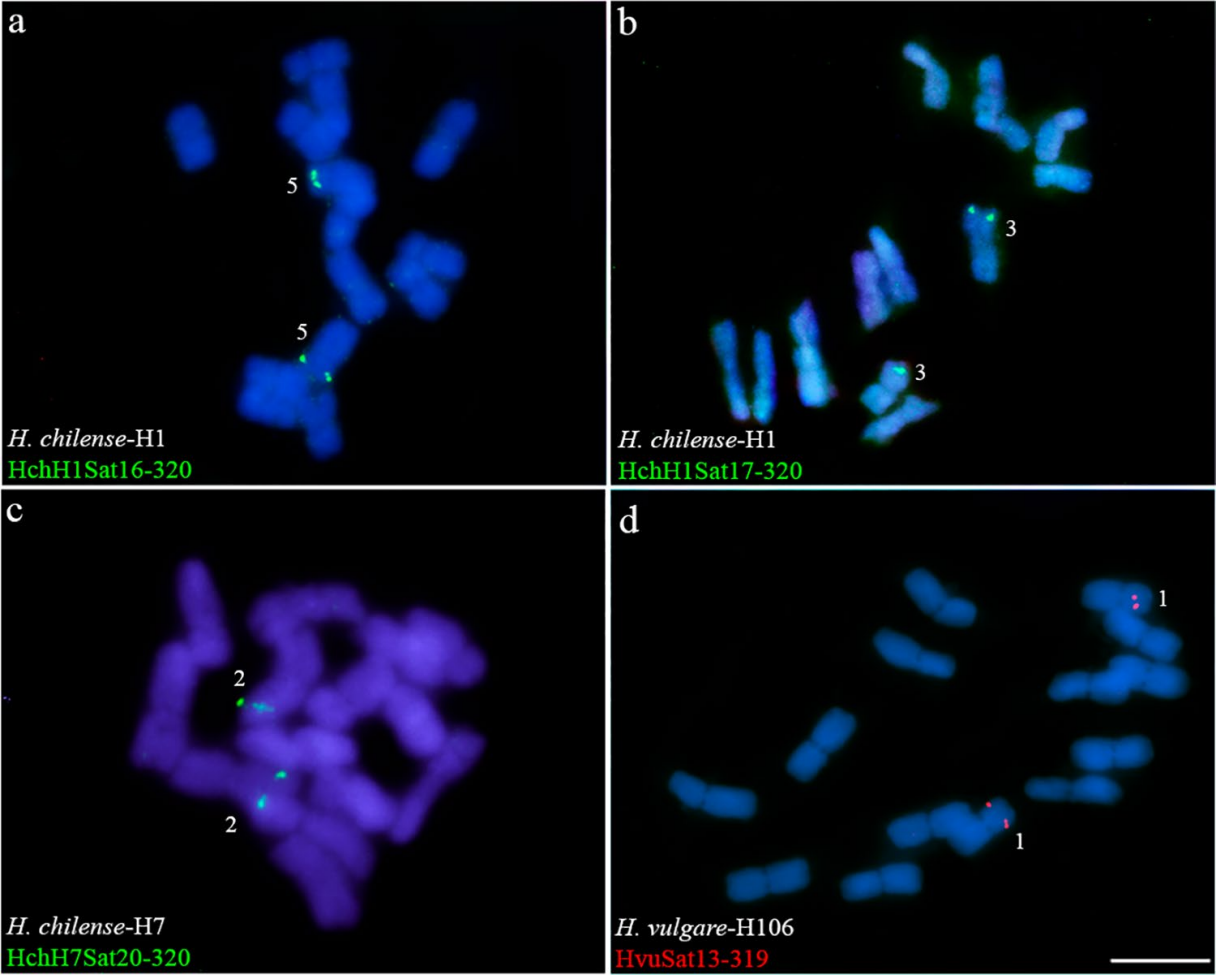
Cytogenetic visualization by fluorescence in situ hybridization of satDNAs included in GR12 in metaphase chromosomes. DNA was counterstained with DAPI (blue). SatDNAs from *H. chilense* and *H. vulgare* were visualized in green and red using antidigoxigenin-FITC and streptavidin-Cy3, respectively. **a** HchH1Sat16-320, **b** HchH1Sat17-320, **c** HchH7Sat20-320, **d** HvuSat13-319. Scale bar = 10 µm

Table 5 summarizes the FISH data of different homologous satDNAs among the accessions studied. This summary highlights how different satellites have different distribution patterns in different accessions. For example, homologous satellites of GR1 have a different hybridization pattern on chromosomes 1, 4, 5 and 6 when comparing *H. chilense* with *H. vulgare* (Table 5). In the case of homologous satellites of GR2, the hybridization signals differentiate chromosomes 2, 5 and 6 of the two accessions of *H. chilense* (Table 5). Chromosomes 2, 3, 6 and 7 have different hybridization patterns between *H. vulgare* and *H. chilense* (and between the two accessions of this species) for the homologous satellites of GR3 (Table 5). The hybridization patterns of GR4 satellites are very different on the 7 chromosomes of the two accessions (H1 and H7) of *H. chilense* (Table 5). And so, we could continue with most of the satellites studied (Figs. 2–4 and Table 5). This can be seen in Fig. 5 as an additional comparative example between the three accessions. Figure 5 shows four out of five homologous satellites of GR 12 (H1Sat16, H1Sat17, H7Sat20 and HvuSat13) that have FISH patterns specific for each accession analyzed and could be used as chromosome markers.

### BLAST search of satDNAs to the genome of *H. vulgare*

Taking advantage of the reference genome of *H. vulgare* (https://www.ncbi.nlm.nih.gov/datasets/genome/GCF_904849725.1/), we have traced the genome assembly with the satDNAs isolated from this species. The number of hits found for each satellite was proportional to the estimated abundance for each satellite, although not in all cases (Table S4). However, an important number of repetitive units from each of these satellites have been omitted from the total assembly analysis if we compare the number of hits revealed with the expected number of repeats (based on the proportion of each satellite and a genome size of ~ 5 Gb) (Table S5). Moreover, many hits appeared at the unplaced contigs, particularly for the most abundant satDNAs (Table S4). On the other hand, a coincidence between the positions of BLAST hits in the assembled chromosomes and FISH signals was found for most satDNAs. However, in addition to the regions detected by FISH, some satellites in the genome assembly using BLAST alignments were additionally scattered in other regions and different chromosomes. In some cases, especially when the satellites are long, the alignments revealed that an important part of the matches are incomplete sequences in the assembly. This is particularly the case for HvuSat05-5500 and HvuSat06-4925, which showed a dispersed pattern and homology to LTR retrotransposons and a scattered BLAST pattern in the assembly of *H. vulgare* genome. However, the case of HvuSat16-483, which was not located by in situ hybridization, is different: BLAST pattern in the assembly of *H. vulgare* genome is also scattered but most of the matches aligned completely or almost completely repetitive units and, in addition, some contiguous repeat units were detected, usually two repetitive units in a row, but in some other cases three or even six in a row (Table S4).

We also checked for the presence of repeats of the different satDNAs identified in *H. chilense* in the *H. vulgare* genome. Table S6 shows the number of hits of each of these H1 and H7 satDNAs in the *H. vulgare* genome assembly. The results are compatible with the results presented in Table 4. The numbers of hits of the satellites of the GR1, GR3 and GR12 groups were the highest in *H. vulgare,* which were also the ones detected by RE2 in the two species. The satellites of groups GR4 and GR11, which were not detected by RE2 in *H. vulgare* but tracked in the raw reads, also present an important number of hits, although much more discrete (Table 4). Of the remaining groups, most satellites were very poorly represented in the *H. vulgare* genome (Tables 4 and S6). Interestingly, (peri)centromeric satellites from *H. chilense* yielded very few (HchH1Sat07-493/HchH7Sat07-484) or no hits (HchH7Sat14-2790) in *H. vulgare* (Table S6) according to the search performed among the raw reads, that only revealed traces of these satellites in the *H. vulgare* genome (Table 4). *Hordeum chilense* satellites that showed a dispersed FISH pattern (HchH1Sat09-1236, HchH1Sat23-4077, HchH7Sat11-505, HchH7Sat12-728 and HchH7Sat23-692) or that did not give a signal in the hybridization experiments (HchH1Sat24-1932, HchH7Sat25-82, HchH7Sat27-410) displayed compatible BLAST results in the *H. vulgare* genome, which allows us to ensure that these sequences show a dispersed pattern in both species, in some cases sufficiently accumulated in *H. chilense* (but not in *H. vulgare*) to give FISH signals (dispersed) or not, explaining the absence of in situ hybridization signals. In addition, HchH7Sat27-410 sequences were not present in the genome *H. vulgare* as the result of the screening of the raw reads (Table 4).

## Discussion

### The contrasting satellitomes of the different barley accessions

Both species analyzed in this article have an undoubted interest in agriculture. One, *H. chilense*, is a wild species with the potential to add value to crops of other grass species, and the other, *H. vulgare*, is one of the most important cereal crops in the world together with wheat, maize, rice, or millet. In addition, sets of both cultivated (*H. vulgare*) and wild (*H. chilense*) barley addition lines in a hexaploid wheat background were developed several decades ago (Islam et al. 1978, 1981; Miller et al. 1982), which have an enormous potential to transfer desirable traits into wheat but also in plant meiosis studies (Calderón et al. 2012, 2014, 2018). Although both congeneric species belong to lineages that were separated no more than 9–12 million years ago (mya) (Blattner 2009, 2018), the profile of their satellitomes is very different. For example, they share a satDNA family composed of repeats of 337–338 bp (HchH1Sat01-337/HchH7Sat01-337 and HvuSat01-338 satellites of the GR1), which is the most abundant in both species, between four and five times more abundant in *H. chilense* than in *H. vulgare* (Table 4), and widely conserved in other grasses being known as the Afa family (Nagaki et al. 1995), closely related to the pAs1 sequence (Rayburn and Gill 1986). Furthermore, both wild and cultivated barley species share three different but related satDNA families composed of repeat units of 118 bp (included in GR3), which are among the most abundant in both species and some of them are also conserved in other Poaceae (Table S2). As an example, the abundance of HvuSat03-118 (*H. vulgare*) is twice the abundance of their *H. chilense* counterparts (Table 4). However, in addition to the important quantitative differences found in both species for these satellites, large quantitative differences for the rest of satDNAs analyzed in this paper have been shown. Only traces of most of the satDNAs of *H. chilense* remain in *H. vulgare* and vice versa in such a way that species-specific dominant satDNAs in one species are characterized by the presence of low-copy counterparts in the other species. This entails that, in most cases, our satDNA mining failed to isolate these counterparts. However, we revealed these traces by tracking the species-specific satDNA databases with the raw sequencing reads of every species (Table 4). Therefore, after all, both species share all satDNAs of their satellitomes except two low-represented satellite families of *H. chilense* that were not found in *H. vulgare*. As predicted by the "library hypothesis" (Fry and Salser 1977), differential amplifications of each satDNA family have occurred in the two species analyzed from the same common ancestral satellite library. In this case, moreover, it appears that the divergent paths of wildlife and domestication have led to an accelerated divergence in satDNA profiles of barley species. Important changes in satDNA content between wild and cultivated species have been associated with domestication in maize (Bilinski et al. 2015). Interestingly, the differences between the satellitomes of the two accessions studied of *H. chilense* were found remarkable (Tables 4 and 5; Figs. 1–6). It has been proposed that *H. chilense* consists of different morphologically and genetically distinct groups and our results contribute to support this view given the differences in abundance and location of most satDNAs between H1 and H7 accessions (Tables 4 and 5; Figs. 1–6). In addition, our results support the fact that H1 and H7 *H. chilense* accessions were selected to generate a *H. chilense* F2 population due to their morphological and ecophysiological differences (Hernández et al. 2001).

Although it is not easy to explain what has happened for each satDNA in each lineage for whether the reduction in the number of copies in one lineage and increase in the other lineage has occurred or vice versa, our results suggest that most of the differences are due to amplifications of some satellites versus others in each lineage. Although the library hypothesis does not foresee the emergence of new satDNAs, our data support the emergence of some new satDNAs in one or both accessions of *H. chilense* either from new random sequences (HchH7Sat22-364) or from pre-existing satDNAs (HchH1Sat02-92, HchH1Sat14-88, HchH1Sat21-332 and HchH1Sat24-1932) as occurred in other plant and animal species (Navajas-Pérez et al. 2005; Ruiz-Ruano et al. 2019; Sales-Oliveira et al. 2024).

The second most abundant satellite in *H. chilense* H1 is indeed one of those. HchH1Sat02-92 (GR2) is a 92 bp satellite. The homologous to this satellite in H7 accession (HchH7Sat06-46, GR2) is just half as long (46 bp), as a satellite of the same group found in H1 (HchH1Sat18-46). Interestingly, HchH1Sat02-92 is represented 30 times more in the H1 genome than HchH1Sat18-46 and up to 3 times more than HchH7Sat06-46 in H7. In addition, hardly any of its relics remain in *H. vulgare* (Table 4). These results suggest an enormous amplification in H1 of a new satellite that has emerged from the duplication and divergence of a shorter ancestral satellite. This is in agreement with longer-length satellites originated from different duplication and divergence cycles of shorter satellites of diverse random origin (Navajas-Pérez et al. 2005; Ruiz-Ruano et al. 2019; Sales-Oliveira et al. 2024), probably by unequal crossing-over (Smith 1976). However, it contrasts with the origin of satellites from other types of tandemly repetitive sequences such as ribosomal DNA (Stupar et al. 2002; Neumann et al. 2003; Jo et al. 2009; Almeida et al. 2012) or dispersed repetitive sequences such as the different types of transposable elements of eukaryotic genomes (Meštrović et al. 2015; Šatović-Vukšić and Plohl 2023), as we have found for several barley (Table S3) and wheat (Gálvez-Galván et al. 2024) satDNAs. As can be seen in Table 4, the longer satellite of GR2 group is practically replacing the shorter satellite in H1, the same as occurs between the 44 and 88-bp satellites of the GR10 group, although there the amplification of the longer satellite has not been so dramatic.

On the other side, the second most abundant satellite in *H. vulgare* (HvuSat02-444, GR25) is poorly represented in *H. chilense* and, in fact, was not detected in this species using the satDNA mining protocol. This satellite is very curious because its repetitive sequence has a structure that resembles the structure of a MITE (Miniature Inverted-repeat Transposable Element) since a sequence of 174 bp (in this case a non-coding sequence of unknown origin) is flanked by two imperfect inverted repeats (Figure S4). However, in this case, the flanking sequences, which in DNA transposons and MITEs are called TIR (Terminal Inverted Repeats), are composed of the repetition of perfect and degenerated GAA (TTC) trinucleotides, which distinguishes it from transposable elements. Notwithstanding, this structure suggests an amplification mechanism of this satellite that would mimic the amplification mechanism of other satellites from MITEs and DNA transposons (Meštrović et al. 2015). Indeed, our search using RepeatMasker revealed its homology with EnSpm elements (Table S3). A set of clones composed of repetitive sequences similar to HvuSat02-44 was identified by (Kato 2011) (Table S2): the sequence of clone pHv-1966, for example, consisting of two partial units of the HvSat02-444 sequence (not shown). The (GAA)n region of HvuSat02-444 is similar to the GAA satellite sequence, a 334-bp composed of perfect and degenerated GAA trinucleotides (Dennis et al. 1980; Pedersen et al. 1996), that accumulates in different loci at a certain distance in both sides of the centromere on all *H. vulgare* chromosomes (Szakács et al. 2013). It is possible that FISH probes of this satellite cross-hybridize with (GAA)n-rich sites and, vice versa. What is remarkable is that Repeat Landscapes plots of each of the two parts of this satellite support that the 174 bp non-repetitive part is an ancient satellite that in *H. vulgare*, but not in *H. chilense*, has been recently amplified after its assemblage between the (TTC)n/(GAA)n inverted repeats (Figure S5).

### Barley satellitomes in the framework of breeding

The correct identification of each chromosomal pair in species of agronomic interest has been an important objective in recent years. Thus, in the case of barley, several markers, either derived from its genome or other grasses, have been used for this purpose. Several satDNAs such as pSc119.2 from rye (Bedbrook et al. 1980), the Afa family from wheat (Nagaki et al. 1995) or HvT01 from barley (Belostotsky and Ananiev 1990) are routinely used for such identification (Prieto et al. 2004b; Rey et al. 2018; Jouve et al. 2018) among other repetitive markers (Kato 2011). But, above all, it is the GAA sequence (Dennis et al. 1980; Pedersen et al. 1996) that has been most used for such identification and is the one we have used in this study (Figure S6). In barley, different types of microsatellites tend to accumulate in different regions, especially heterochromatin, forming loci visible by FISH, which tend to have different patterns between chromosomes and between different barley accessions, making them useful markers for chromosomal differentiation within and between *Hordeum* species (Cuadrado and Jouve 2007; Carmona et al. 2013). In this article, we provide the genomic information of the satellitome of *H. vulgare* and *H. chilense* with the analysis of 70 satellites from three different accessions that have revealed the existence of a total of 46 satDNA families, most of which had not been previously described and that provide a large number of new markers that differentiate chromosomes both within and between species as well as between the two accessions of *H. chilense*.

In this context, satDNAs are located in regions important for the correct homologous chromosome pairing during meiosis, most in the subtelomeric and peri(centromeric) regions. Centromeres have been extensively studied in barley (Houben et al. 2007). The functional centromere region of barley chromosomes is composed of an LTR/Gypsy-like retrotransposon conserved in several cereals, the centromeric retrotransposon (CR) *cereba* (Presting et al. 1998; Hudakova et al. 2001) and a short 6-bp species-specific GC-rich satellite (Hudakova et al. 2001; Nasuda et al. 2005) that we have not identified in this analysis. Only a fraction of the centromeric DNA is utilized in the kinetochore assembly (Houben et al. 2007). Thus, the pericentromeric region would also be composed of *cereba* elements and of the 6 bp GC-rich satellite as well as probably other sequences that we have found in this study. We have isolated a satellite, HvuSat15-1590, present in the (peri)centromeric region of all the chromosomes of *H. vulgare* (also present in six of the seven chromosomes of the two accessions of *H. chilense* in in situ hybridization experiments) which is not homologous to *cereba* but to retrotransposons of the LTR/Copia type (Table S3). The satellite HchH7Sat14-2790, related to LTR/Gypsy retrotransposons (Table S3), was identified in accession H7 of *H. chilense* and it also hybridizes (peri)centromeric to all chromosomes of both accessions (H1 and H7) of *H. chilense*. However, this satellite was not detected by FISH signal in *H. vulgare* chromosomes. In potato, several very long chromosome-specific satellites have been amplified from retrotransposon-related sequences (Gong et al. 2012; Zhang et al. 2014). Indeed, an association has been found between transposable elements and newly emerged (peri)centromeric satDNAs in several plant species (Gong et al. 2012; Sharma et al. 2013; Zhang et al. 2014; Meštrović et al. 2015; Vondrak et al. 2020). It has been proposed that centromere-specific satellites have originated during evolution by nested transposition (Sanmiguel and Bennetzen 1998; Hudakova et al. 2001) and that there would be a tendency to replace CRs by satellites (Langdon et al. 2000; Hudakova et al. 2001). In this context, HvuSat15-1590 and HchH7Sat14-2790 might provide new repetitive elements that could emerge as new dominant centromeric satellites in the future. The different organization of the HchH7Sat14-2790 satellite in *H. vulgare* and *H. chilense* (probably scattered in a few locations *vs.* accumulated (peri)centromerically) is very relevant in this regard. So is the emergence of HchH1Sat07-493/HchH7Sat07-484 homologs in the (peri)centromeric region of a chromosome pair of the two accessions of *H. chilense* but not in *H. vulgare*. These reflect the enormous plasticity and dynamic capacity of the (peri)centromeric region of barley and the set of satDNAs found in this study would provide the substrate for future DNA sequence replacements at centromeres.

Although some subtelomeric satellites such as those of GR1 are located in all chromosomes, in the two barley species there is great variability at the subtelomeric level. Such diversity is reflected in specific patterns of different satellites for each chromosome of both species which, as we have suggested for wheat (Calderón et al. 2014; Aguilar and Prieto 2020; Gálvez-Galván et al. 2024) and for barley itself (Serrano-León et al. 2023), may be very relevant for the correct pairing of chromosomes during meiosis which is also of interest in these species since in breeding the use of addition and substitution lines is very frequent. Cytogenetic studies of homologous pairing between a pair of *H. chilense* chromosomes lacking the subtelomeric region on one chromosome arm have demonstrated that the subtelomeric region is important for the process of homologous chromosome recognition and pairing (Calderón et al. 2014). In this work, we have identified several satDNAs in different barley subtelomeric chromosome regions that might suggest conserved functions of these DNA sequences in early meiosis. These subtelomeric satDNAs can contribute to shedding light on the putative role of these subtelomeric regions during chromosome recognition and pairing stages in barley meiosis.

## Supplementary Information

Below is the link to the electronic supplementary material.

Supplementary file1 (XLSX 98 KB)

Supplementary file2 (DOCX 54 KB)

Supplementary file3 (DOCX 17 KB)

Supplementary file4 (DOCX 18 KB)

Supplementary file5 (DOCX 21 KB)

Supplementary file6 (XLSX 155 KB)

Supplementary file7 (XLSX 45 KB)

Supplementary file8 (XLSX 57 KB)

Supplementary file9 (XLSX 7977 KB)

Supplementary file10 (XLSX 19 KB)

Supplementary file11 (TIF 11115 KB)

Supplementary file12 (TIF 4723 KB)

## Data Availability

All raw data included in this work are available in the article.
