# Supplementary material for: Highly divergent satellitomes of two barley species of agronomic importance, Hordeum chilense and H. vulgare
Source: Plant Mol Biol. 2024 Oct 2;114(5):108. doi: 10.1007/s11103-024-01501-5 (PMC11447152; doi:10.1007/s11103-024-01501-5)
Supplement: Supplementary file 2 — Supplementary file2 (DOCX 54 KB) [file 11103_2024_1501_MOESM2_ESM.docx]

Additional file. Sequence in fasta format for each satDNA family of *Hordeum chilense* (H1).

>HchH1Sat01-337

AGTAAATGCATGAAAAATAGCAAATGATGTCAGAAACGGTGAAATTTGAGGATGTGGCTTTGAATGGTGCATATTGAATGCACAAAAAGTCTGGAGTTGAAATAAGTTTAAAAAAATGAAATGCCTTTGTAACAGACGAGTTTTCGTCTGAAACCCTCATACTTCGAAAGAGATTGTCCATTTTGTACACGAAGTGCATCCAGTTTTTGCCGTAACCCTCTCAACTTTTTAGCACATGCTATGTGGGTGAAATGATGATACCATGCCAACTTTCAACATTTTCAGAGTTCATTTGTAGTGCTTTTCAATTTTAGGGTCATTTTAGCTCAAAAAAATC

>HchH1Sat02-92

TCGTCACTCCGCCGGACGCTCCATCGTCACTCGGCGAGACACGCCTTCGTCACTCCGCCGGACGCTCCATCGTCACTCGGCGAGACACGCCT

>HchH1Sat03-118

CGGAGTGATTTCCACGATTGACGAACCCCGGGGTGCGTTTACGTGTCGGTCATCAACACTCGCGGTTTTGGCCGATTCTGGCCCGTTTCGTGGACTATTACTCACTGTTTTGGGGTCC

>HchH1Sat04-118

CCCGCTGCGATCCGAACGTTCGGGGAACCCCGGGGTCCGGTTACGGGGAACTCGTCAAAACTCGCAGATTTGGCCTATTCTGGCCAGTTTTCTATACTATTACTCACTGATTTTGGGT

>HchH1Sat05-334

TGCCGCGAGGTCTTGCGCCGGGCCCTCTTACACGTGTGGAAGAGTGGTGACGGGTGTAAGCACCCGGCACGCGGCTCCGTAGCGTGACCCCCCTCACCGACGTCGCTGCCGCCGTCACACGGAGGGTGGAAACGGCCACGTCGGGTAGGCACGGAGGGAATCTTGCGGTGGGTTGGGCCCGGACCTTGATCCGAAGTGTACCTATGGGCTGGCCTGTCACAGCCAGGTGGAAATGTCCCTCGGTCAAACCCCGTCCGGTGAAAGTCAAAGGGGTAGATCCGTCCGGTCAACAGTGTCAGGGTTTGACGGGACGGGGGACTTTGGGGGGTAGCTA

>HchH1Sat06-352

GACAGCCGTTTCGGGGGGGGAATGACCCCCGGAGCACCGGAATGCCACCAAATCTTGCAAGTGGACCTAGGATATGTGTGAAAGTGTGTTGGAATGTTAGATTCACCAAATGGTGCAAGGCATGGCTCCCCGGTGTGACCTTCCTCACCTTCGGGCGATAACACTTAAGAGATTCCTCGACGTCTAGGGTGAAGTGTCCCGCTGCGGCTATACCGGAGGCTAGAATGTGCCAAAGGGTGCCACACCTGGATTTCCATGTGTCCATGGACTAAACAAACCTAAACCTATGAGAAAGTACATTGGTGGAACCTCGCGCGGAGAAATCTAGGGGGTAGACCACCCGGGGCCCACA

>HchH1Sat07-493

AGCAAGGCCAAGTGAGGCAGCGACTAGCGGTTCGTCATCAACCGTGTCCCCCGTTCCCTCTCAGAACCACGAGCTAGCCTTCATGCGATGCTCCGGTTTGTTACATGTACGTGTCAAAGCTTATCTGAAACAAAGCCATAATTTTTACCACGACATCTTGGTGCCATGTCATGAAATCATGCAAATTTACATGTATTTCAGAGCATTTTTTATTTTCTAATTTTAAAACCTAGATTCCTAACGTTTGTGTCCAAAGAGTGACTCCAAGATGTTTGGAATTCCTTCCCATTTATACCATGGGGCATAGTAAGGACACAAATATGAATTTTCCAACAACTTTTGCCTCATCGTGACGTGTTCATGTATTTCAAATTTGAATTATGGCCATTACATGGCTAGAAACTCACTTGTGGTCTTGAAAAGGCCAAATGAACACCGAAAATGTGCAAATCTTAGCATAATGGTTGTATTCGTGCGTTATAAATGAAAAAAA

>HchH1Sat08-570

ACCAAAACCCCAAAAAATGAAGTTTTGCATGCTCTATCACCCATCCCGCTGCCATCCGCATGTTTGGGCAAGAACATGCATCGTTCTTGGACGAAAATGTGCATAGCAGCTCTTCTGTGCTCTTATCATTTACACGCAAAATAATAGGTCATTCTGAGTGGTGAAAGATGGCTCCCCGACGCGATCCACTTACCTGGGAAGCTCGACTCACGGTCTGCCCCGGTGCTGAAATCCATTTAGGTTGTATCCGTAGAACGGGTGTAATCTGGTCATACGAACTCCGTTTGAGCTCCACGACCTGCCAAAATGACCGCAACGAAAATTCGTATACCCCGGTGGCCCACTTCATCGGCTTTGGCAATGTTTCGCAGGCCAAAAATGGCTTGCAAAATTGAGTTCCTGATGATTTAATCACTGGCGTGCACACTCCTAGCCTCTACCATCGTACCGTGAGCTCACCCACAGACACATATTGGGACAATAACACATAGAATAATGCGATATGATTCTATTTGTGCACCAACAAGGCCTACATGTCAGTCTGGATAGAAGGAGGAGGCTCCTCGGTGC

>HchH1Sat09-1236

CATTGCACAATGACGAAAATCACGCAAAAGTGACACACAAATGATTGTGTAAGAGATTCTTTGCCAGCATCCACACGTAATTCATGTTGGGATATTTTATTTATAAGAACTAATTAACATGGTAAATACAAAAGGATGACTTAGATATAACAAACGAGCCATGTAATCCACCAGAAGATCAATAATAGGAGTACAATGATGTTTGCATACCATGGGAAAAATCGAGGCGAAAGGATGAGGTTGACATAGATTGGAGAGTCCAAAATGCCAACTCTACATTTGGAAACTATGATCCTTCGTGTGAGATGCCTTGGTTTTCAAAGGGCAGTACATCACAACTTCCGCCAGACATTACCAATTTTTTTCCACGGGGTATTCACATCATGCCACGGTGACACACCCATTTTTGTGATTTTCGGAATACATCTGCATTTCCTCCAATTAAATTACTAACAAGCCCAATGTTGGTGGAAAAAATGTCTTAAAATTAGCAAACAAGTCATGAAATGCGGCCGAAAATCAATACAGGGGTTAATAGTTGATTGTCTGCCGATGAAAATAATTCGAGGCGAAATGATGAAGTCACAAATTCATTTGAAATGAAAATATGATTGTTGTGCATAAACAAATAGCACACGGTTGGTTCACTAAAACCGTGTGCTTTCTAAACCGTGGACGATGACACCTAATGCGCGCAAAGTGAGTGGTGCGTTCTGATTGGACAAAACCCAAACCACACGGATCGTACGTCTGCACCGTAGGATTCACCCAGATCGAACGGCAGTCCTTATACCTCTCGTCAACACACGTTGTTGAAGCTGGGATTTGATTTATATGCACTAAATGCCCCGTAAATGATAAAAAATGCTGAAAAAATATCAACCGAGCCCATAAATGCGCCAGAAAATCAAACCTGGCGTATAATGATGATTGAGTACCGTGGGATAAAAGTTGGAGGAGAGGCGATGAAGACACCATCGATTGGAGTTCGATTTGCCACTTCTCCCTTTGGAACTATGATCCTTCTACTCAGATGCTATGGTTTCCAAGGAGCGATCATTGTAGCTTGTGCCAAAATTAACCAAATTTTTTCCACGGTGTCTCGAGATCACGCCATGATGACACACCGATTTTCGTGATTTTCTTGAGTCCATTTGCATTTCCTGTAATTAAATTACTGACATTTATTTAGGTGGACACATCTCCCATCCGAAATGCTTGACAATTCTTCTCATT

>HchH1Sat10-652

CGACAAGTTCGACCGGTTTTTGAAAACACCGTAAAAAATTCAAAAAAAGGGAGACCTCCGCGTCACATCATCAAATGTGCCCTACCAACTAGTAAAAATATTAAACTTGGAATACCGATGTTTTCTTGAAAAAGTGTTCTCAAAAACGACCTACCATGAACGAAGATTCATGGCTTTCAAGCCAAACGAGCAATGATATGGCCACATTCGTTGAATAGTTTGTGATAATATGCCCAAATTTGGCGCATGCCTCCATCTTGTGATGGCAAACAATGTTGCCGAAGAGAGGTTCCAACTTGTTTCACAAAAAAAACCATTTTTCATTTTTTGAATGCCAAAAACACGTGTTTTTCGTGAAGCGGCTACAAATAGAGACATTCCAAACGGCGCCATTCCATGTCTATCTCAAAGTAGACCCTATTTTACGGACGGTCGCCAAAAATCATGCATTTCCGACCCTCGTAGCTACTCCCGGCCATTCAGACAACCATCGACCGATTCAGCTGGAACCGGCTGGAATTTGAACTGCGGGTCCTCCATAGCTTGCCCGTTATTTTTGCTAAAAATCATTTTTAGCTTCACAGGTAGGCATTTCATCATAGAATCACCAACAGATATGCATGATCAAACCCCTAGCCACGGACGGCCGACG

>HchH1Sat11-336

CACCCCGGGGCCCCAAGACGGGCGTCTACGCATGCCACGTCACTTTCACACATGCAAGAGGGCCCGGTGCGGTGTTTCTGTGGCATTGCTACCCCCGGAAGCCCCACCGACCGGACTAACCCTAGCCCCGTTGAACACGAGATCTAGCCCTTTGACTTTCGCCGGACGGGCTTTGACCAGTTGACTCTTCCACCTGGTTGCGATAGGTCAGCCCATAGGAACAACCCCGAACACGGTCTGGACCCAACCAACCACTAGATTCCCTCCGGTTCTACCCGACGCGCACGATTCCCCCCTCCAGGTGACGGCGGCACTTCCGTCCGTGGAGGGGGGGCA

>HchH1Sat12-118

GCACACTGTTTTGGGTTCCGGGGACGATTTTCGGGGCCCGTGACCCCCGGTACACGGTTTCGGGGGCGACGTCAAAACTCGTCGTTTTCGCGTTTTCTGGCCGTTTTCGTGGGCTATA

>HchH1Sat13-344

CCAAACCTGACCCGAACGAAGATAAAAGTCCATTGGTCAACCCTCGTACGAAGAAATCTAGGGGGGGTAGATCTGCGGGGCCCACGGGCTAGGGTTTGGCCGGAAACCGAGCACCGGAGCGCCGGGATGCCTCCAAATGTTGCATGTGTCCCTGGGATATGCGCAAAAGTGTGGTGGGATGTTTAGAATTGCCATATCTAGCCCCCCGGGCGCGAGGCTGTTCGGAAGCGTGCGATGACACTTGTGAAGGTTCTGCACCTGTAGCGGAGAACTCCCACCACGGTCAACCGGAGGGAAAACCCGTGGAATCTTGGGGTAAAACTTGTGCTACCAAGTTGCTGTGA

>HchH1Sat14-88

ATTTGTACTAGGTTATAGAGCTAGTTCAACTCTAGTGTAAAATTATTTGTACTAGGTTATAGAGCTAGTTCAACTCTAGTGTAAAATT

>HchH1Sat15-503

GAAAATGGTACGATCCCAGAAATCCTCAGGATCAGGCACGCCGTCATCACATGGCCCCTGTAGGGTGTGGTAAAAGTTTGGGCGCGTTCCCGGTGGGCCTCGCCCGAGGCCGCTTGTAAACTGCACCATCTTCGAGGTAGTCTCTTGGTATCGAGAGGGAACGTGTCAGGTTTGTGAAGGAAGTGTTGGTCATGTTGCTCCGTTGGCATCGAAACTCCTCGTGCTCTCAAAGGAGGCCATCGAATGACACGTGTCAGGCCCCCGCATTTTTCTGACCCGCCTACAATATTTGAGACATTTATCGCACTTCTAGGGTTTCAGCGTCGGAAATTGCAGATCCGCAAGGCCACACATGAAATCATGCCCAGAAGCAGCATGGAAAATCCTATGTGATGTATTTGTGACATGGGCATGCCTTGTGCAGGCGTGGGAGGGGCAGGCCCAGCCATCGGGGGTGCGAAATCACCTGGGCGACATGCCTGATCCAACCGTTTGAAGCCTCG

>HchH1Sat16-320

GGGTATTTTCCTCTCGAAAACCCTAGAAAATGCATGCAACGTGGAAAATTGTTGTAAATTGGCATGCATGCTTGTGAATGCCATGCCTGGGTGTGGAAAAAGTTTTGGGTCATTCTGTGGAGTTGAAAACAAAAACGTCCTTCGGAGGTGGCCTCCGGTAGACCCGAACAGTGCTGGTTACACATGGTGTTGGTGATTGCATGCATGAAAGTTTACAAAAACTTGGCACCATGTCTGCATGACCGAAGAAGGGCCCCACGCAAGATTTCGACGAATCCGGACACAAAACGCACGTTGCGTCACGTCCGGGCGTTGTTTTC

>HchH1Sat17-320

ATGCAAAAACAGGCAAAAACAGATGCCGGACGTGACGCAACGTGCGTTTGGTCTTCGATTTCGTTCAAATTTTGCGTGGCACAATTGGATGTGCATTCTCAACTGCTGGCAAAAGTTGGGTACATTTCATGCATGCATGCATGTACATCATGTGCAACCCACCGGTGTCGGTATGGGCGGAAGGGGTGTTACAATGCGATCTTGCTACTCCCCTTCATCAAACGGACCCAATTTTTTTCCACGCTTCCTAATGAACTTCACAAGGAGGCACGTCAATTTTTTTTATTTTCTGACAATCCGTGCCTTTTCTGTGAATTTCC

>HchH1Sat18-46

GCTCCATCGTCACTCGGCGAGACACGCCTTCGTCACTCCGCCGGAC

>HchH1Sat19-917

AAGGCCGTCGGCATAGATCCTCTGCCACGTCATCGATCAGGTGATTGATCCGGATCCCGTCAATAGTTCGGTAATTGTCCCTTTGATGAAGAAGTTAGGATGATGGGAACCCATCGAAATGAATAAATAGCATGCATAAATGTCCATGCTTTTGTCTTGGAAGTTCCAGCAAAACATTTGTACTAAATATCTGTCGGTTACCACTTATGCAGATCATCCCTGGTGTCTCCAAGGAACTTCTCAGCAAGTGCAGATGACCCATTCAGTCCAAGTTCTCTTCCTGCAATTGATCTGATGGCAGCCTCCAGACTACCTTCAGCTTCAACAACCAAACAGTTGATGTTGGCATAGGGCATTGGCAAGATTTTGTGAAAGAGAGGGGAAGAGATCTGAAGAGCTCCGAAAGGGACGTCTCTGTTCCCATCTTACAGGAACTGTACCATAATAAGGTTGAATCTGCCATAAAGCTGCGCGGATTACTTTCTCCATTCGGACTCACTGGAATTGATGCTATTGATCTCAATCAGTCCTACAAGGACAGAATTGGTGGATTTCCGAAGTATATGACTACACTTGATCTTATGGTTATACTGCATAACATGTCCGAAGTCCACTGGTCCATTTACCTGGATAATCTCTGTCGCATAAAGGTACTTGTTGTAGAAGATTGGAGCACAGGTCTTGGAATTGCTTCATCCCCCCTGTTTCACGACAACGGCATGATCTTTGCATCTCCTGAGGGAGATACAAGACTACGTTTTAGGGGCGAGTGTGACGCTTCAGCAATAGGGGAACTGGCCAGTTTGTCTTTCTTTATTTGGCTGGGGCCAGAGGTGTTCAAGGCACAGGTATATCATGACATTCCTTGTGCGAAAAATGATTCAGTCACTACCAGAATAACTGCAGCTGCCGACGGC

>HchH1Sat20-245

TGAATACCCCATTCCACCAATAAGATCAAAGGACAGTGAATATCCCAGTGTTTGTGAAACTTCACGCAACAAAATCTTAAATATCATGAAACAAATGAGCAATTGCGAGAAACTCTTGGACATCAAAACAATCTAGCTTGATCGGATGGCTCGGGATGCGGTTGACGCCAGCCAAAGATCAATCGGTGGAAACAAAGAGTTTTTGACCCATCCCATTAGTTTGTGTGAGTTTCCTCCCATGTTGG

>HchH1Sat21-332

GCGCAAGATTTGGTGGCATAGCTACCCCCCGAAGGCCCCCGGCCACACTAGTAGACATGCGAAGAACAATCCGCACAGTTGAAATTTTTCAGATACTTCTTCATCTAAAAAAACTTCTGCAACTGCTTCACATCGGCCATCACATGTGCCAGCACTTCAGAACAAGGTCTGGGACAAACCACCCTCCAGATTCCCTTCAAGTGGGACCGAAACCTGTGTTTCCCCACTCCAAGTGTCGGCGGCAGCGCGGTCCGTGAGGGGGGTCACACTCCGGAGACGCGTGCGGGGTGTTTGCACCTGCCACCACACTTCCAGACATGTATGAGGGCCCA

>HchH1Sat22-1044

GGGGAAAAAGATCATCAACAGGCTCATATTCTATCTACAAACTGAACATCAGGGAAGAAGAACAATCAGAAATTCAGAAACAAAAACCGCAAGCTACCTGTTCAGTCCAAGAGTGAGGAGGAGTTTTAGATCGTAGCTGCTTGGGAAATACCCTTTTGCCAACCGGATGAACTCTGTTCGTGATGGTGTCAGAACACAGGAATTCTGGATTGCCCTGATCAGGTAGCCAAAATCTGAATAACCTTGGAAAGTGATCCATATAATGTTTCTGTTTCCCAATATGCCCAGGCTATTGATACCATCCATGAAAGTTCTGAGGTTGATACCTCTAGTGGCATGATCAACAAGGTTCAGCTTCGCCACATCTCTTAGGAATCTGATATTTGTGCTGGTAGAGCTCCTTGCCGTGTAGTCAAAGCAGATATTGAACTGAAAAACCAAGAAAGAGTCCGTTTCAAGCCCGAACCTGGAGTCGGCGATCGCAAAACCTACTTGAACCAGGTCACCCTCATCGAAAATCCTTCGGGCCTGCTCATAGTGAGCATCTGCAGATTGGGGCCCTCCCTGAGGACTGACTACCTCGTCCTTCATGCAAAATTCAGTATCAACTGCCACGTACCACTTGCAGTTTTCATGTCCTAGCAATTCCTTCATTCGTTGATATTCTGTATCAAAGGTGTCTGCCCAAACGTCCATGAAACTTTCTGGCTGCTGCTGCACTTCCAGAGCTGACTCTGTCTCTGTGTCTGTCGGGCGGTCTGCGTCCAACAAAAACCAAGCAATGTCAGATCAAACACAAAACAAGAGTCTTATTCAGCAAGCACTTTAATTCAATTTGGGCCACGACCAGCATACCATCGCCATGACCAGCATACACAATGTCGATCTTGTTACGTGCGCCTTGCGCTTCTTCCTGCGACGCTGGTTCTTTCTGTAGGCTCGGTTCCATGGCGGTGCTCGGTCCCTGGCTTCCATTGCCTGCAAAACGGCACCAAATTATGTACACATGAAGAGTCAAACTCGGCACATAATAAACGAATTAAA

>HchH1Sat23-4077

GGACGACACCATCGGGAGGCCGCGTAGCCGCCGTCTTCGCCGGAGTGGGGTCGCACCCGACGGAACCCGGCTTGGTCGGGAACGCTGGCCTGGCAACAACGACTCTGTGTGGCTGGGTATCCAACCATCGGCAACAGGTCCTCCCCAGCATCGCCTCCGGTGAGCCGCCGGCGGAGAGATTGGGGTTGAGAAGGAGCGCAGCAGCGGCGGTGTAGGAAAGAGAGAGGAGGGAGCGGAGGGCGGGGGGTAAGAGCACAAGTTGCTTGCTGGTACATACACTGTTCCTAACTTAGCAGCTGAGTTATCCAGGTTAAGTTAACTAAGTACCATGCTTTTGGCTTGCTTTGAAGCCCATCCAGTACCACTGGTGAGTGGTGACTGATACCAAGTATTACAGATTCAATTAACTCAGTCCATTGTGGTTTCCTTGTTAAGCATTGAGAGGCTGAGATGGTGGTTGGAACAGAATGCACCTACATTGAGTGGATTTTAGTTTGAACAGAGGATAGACAGACAATGGCCATAAGGTTGCAGTTACAGTTGGATGGGGGCATGTTTGTCTCCCGGTGAAGATGTGAGGGAGGAGGATTCTATCGTGAAAGAACAAGAGGAAAAGGAGCAAGTTCGTGAACAAATAAGCTCATCTTTAAAGGAAAAGAAGACACTGGTTTGGAACATAGGGAGAATGCTGCAGATATTGCCTTTGTCAAACATGTGAAGTATTCAAAAGCAGTTGTTTGATACTCACCAGATACTGGTTCAGAAGGTTCTTCGTTTTTCTGTTCTTCCATGACTTGCAAGCCCTTTTGGTTAGCATTTGTGTCTACACAGGAAAAATACATGTTAGCTTAAACCTCTCACACCTCCACATTTATTGTTGTAGTTAGGATGTTGAAATGAACTCACATCTGTGGAACGATACATAACTAACTGGGCTGAACATCTAGATGTTCAGTTTATTCATGAGCGCCCTTTCGTTTGGTGTTGACAACACCACCACGAAAATTTCTTGATCGTCTATGAGGCAATCCCTTGCATCTGCTGCGTTGAAGAAAACAAACAGCGGGACCGTTGGCTCAATATAAGAATGCAGTGCTGGCCTTGGTACTTTCAAGAAGTACTGTCGCCCATCCTTCTAGTATATGTCACAAGACCCATAGATTTGGATGAACTGCTGTTCCAGCTCAGGTCTTGATTCTAAGTCGCTGACAATCAAGTGAGTGTCGTTGGCCCCAAGAGCTGACATTATATGAAATAGAGCAGATAGCTTATCGCTATCTTTCTCAACCTCGCAGTAACAGCTGTTTCGCAACTCTTCCAAGATAATCCTATTACTTGCTGCAATTGAAGAGTTGAGAACACACTAAGTTTTGTTAGCATTTAGTTTTGCAACAGGAGAGATAATCATAAGAGTGGAGAGATTTGTACCTAGTTTCTTTATCGCTTGAGGCGGATGTGTGCCAACCATGAATATCAACGATCCTCTCAATGTCGGCGCCCACCTCTCCAACATTCCTTTTACATACTTAAGTGTCAACTCTGGCTGTACTTCCGTCTTTAGAAGACTATCAGCAAAAGCAAGTTTGTCTCTAGTTTCTAATCTTGGTATGAGCACATCGTATTTCGTGCTCAAATACCGCAGTAGCATGAAGGATTCATAGTTTGCATCCAAAGGAGCGACTTTCTTCATTCCTTTAATTGTGTTAATTACAGCTTCACAATCTGATACAACTTCCATCCTCTTATATTTAGCTCCTATCTTCAGAAGATACGACATGGCAAGGGCCTCTGCATGCACAGAATCTCTACAGGGTATGTCATAGCAAACAGCAGAGCAGACTATTTGATTGTCCAGCCATACTATTGCGGATATTTTTGCCAACCCTGTGAATCGGTTGTATGAGGCATCGCATTCCCCACGGTAGTACCCTTTTCCTCTATCCGGCCCAACTCGTAGACCATAGTCCTGTACGGTGTACCCCGAGGACGTGTAGAATATGTTCTTCCAATCTTCAATCACATAAATGTGCTTCTTACTTAAATCATCAAGCAATGGTATCCATTTGTTTGTTTCCGTTGTCAACACATCTATCATGATATCGAAACTGGTCATATATATTGGGTATTGCTGGGTGAGCTTTTTGAACCCATTGTGTAAAAGCTGCGTTCTATGATCATGGAGTTGGTATGGCAATACCATGCCTCTGGGCAGTAGTGCTGGTCTGACCTTTTGATGATACATATTCTCAAGAGAGCTAGATCCCAATATATGTGTAACAACTGCGTTATCCACCTTCACAGCATCAGTGAAATCCTCTGCAGGAACAACTCCAATATAAAAAGGTTCTGTCTCAGACTCGGTAAAGGGCCGCACTACCTCCAATAGATTATCAGGAGGGGGCAGAGGTGGTGATATTGGGGTTTTGTGATCATCTGGGCCTGTTGAGAAACACCTTGTTGGTATGCAATATGCTATCATCTTGCTTCCCCAGTTCTGAACCATTTTTGGTCTCATACTTCCATCAACTTGCTGAGCTGAGAATATCTTGCTCGAACACTTCTGCATCGGAGCTTGATCTGTACCAACTTCTGAGGACAGCATGCTGGCTCTGTCTTCAGCATAGAAGTGCCCAGATAAGATGTCCTGGGAGGGATCACTGGAAGAAGTACACTTCGAGGAGAGTGTGGATGGCTGCTGGGCAGAAATTAAGTCCATACCATGCATGGCAAACCGCTTCATTTGTCCGTGAACACTGGTATTGTATGGCCCTTGGTTATCATCAAATATGTCATCATTTGCAAGAGGAATCATCTGTAGAGTGTTATTCTTTCTGCAACAATCAACATAACGAGATCATTATCATGAAGACACCGCAAAGAAAATACAACGAGTTCGAAACTACAATGCTTTTGCTGTAGAGATATGTGACATTTGAGAACAGAAATAGCATAACATATGTTGCAACTTTGCATCATCCACACAATGTTGTGAACGAGGTTGTTAAGCCTGAACTTCCAAAGAATTCTTGTAGCATAAATACAGCACATAACAAATCTACTAAAGTGAATCAATTACAAATAACGCAAATTTGACAGATTGAACACAAACGACAAACAACAAACAACATTGTGCACACCTTTTGAGCACTAATAAAACAAATTTCCCACTGAAGTGAGACCGGTTGAGCACAAACGAGAAACGATGGAAGTTCATTACCTTTTTTGATCTGAAGGATGAGAAAAGGCAGTGTCTTTTGCTGGCGGGAGGTGACCCTTTTCCTTCAAGCACAAACATTCGCCAGGGAGGGGAATGTCGCCACTGGTAGGAGAAGTGACACATTCGCTCAACCTGGATGGAAGCACACCAAGAGAGCATGTAGGCTTCCGGACAAAAGTGAAGTGCATACCATGCACCCCAAACGGCTTCATTTGGCCATTGTATGGCCCTGGGTGTTCATCAAATGTGTCAAGATTCGCAAGAGGAATCAGCAGAGGAGTGTTCTTGTTCCTGCAACAACCAACATACATAAAGAGATCATTATCATGATGGCACCGCAGAAAATACGACGATTTCGAAAATGCAATGCTTTTGCTAGCTGTGCCTATATGGCACATTTGGGGACAGAAACAACAGAACAACTGAGCACAGACAGAATCAATCTTGGAAAAAAATTGTATCATTTTTCCATTACAAATAACACAGATTTCCTAGTGAGATGAAATCGACTGAGCACGAACGAGAGGCGATGAAAGTGCAGTACCTTTGAATCGGAGGATGAGAAAAGGTTGTGTCTTTTCCTGGCGGGAGCGGGACTTCCTTTTCCTTCAAGCAGCCTGCGAGGGAGATGTCGCCGCTGGTACTGGTAGGCGCAGTGCAGCCTTGGTCGCTCCACCTAGATGGAGGCACGGCAGCAAGCGAGGGAGGCCTGAGGCAGGAACCCCTGACGTGGACGTCGACATCGAGGTCGCACTTCAGAGGTGGAGTGGCCAAGGTGGAAGGCTTGAAGATCGGGCCACGAAAGCAGGTCGCCTTCTACATGAGGCTTGGAGGGATCTTCGCGCTCGCGGCTTCGACGA

>HchH1Sat24-1932

AATAATTCAATCGTCTAGGTAAAGGTGTACAACATAATTTTCAAGAAAAATTGAAACCTGACACGTGCACATGCAATTTGCCAACAAGTGCACTTGTGTACAAAAAGATAGCATGTGCACGTATCGCACCCTGCCTCCTATGATTTTGTTCCATGTTTTATCGCCACATATCTTTGGAAGGAAAAAAAGGAAGGTGATTTTCCCATCATAAACAGTTTTGGAGAGATATTTGCAACCAATCAAAAATAGCTACCGCGTGGAAGTTGGCCCCCGTGGCGAGATTGGATCGGCCTCTCCTAGATTATAGGGCGAGGCCATCGAACGTGATGGTGAGAGAGTCAAGAAGGATTCTTTTTTTTGAGGGAAAGGAATTTTGTCGCTTTCCAAAAAATGCACCCATTTTTGAGGGACAAGAAATGCACCTTATAATGTGTAATTAATGAAATTATTATTTAGCAGAAACCTTGCTATTTTGAAGCAATTAAAAAACATCTTACTTGTTTTGGCAAAAAAAATGTTGATAATGGGATAGTCCATTTTGTGGACACTTACGTGTAGTGGCGCATGTTGTGGGCCCAGGTAGCTCTAGGTGCATGCAGTGTGTCATGCTGGCTATGGGTGCACATTGTAGTAACCTCGGAAATACAAGGTCTAGACCGCGGCCACATGAAATCATAGCTGGGAATCGGGTGAAAGTCTTGTGCTAAGCAATTTTGCGGGGTGCGACATATGAATCTATATGTGTGGATGCTTGGCTTGGGGCTGGCGTAAGTGGTGTGGGTCCAGTGCCTGAGTGGTGCACCATGTGGTTCTGGACGGCCTCGGTGCATGCTTTGGATCTAGCTATCGTGAGGGAACGCGTCCGGTATGCGCAGTGAGTTCGTGGGCCGTTCCTCCGTTTCCGTCGGTGCTTTCTCTAGGTCAACGGAGAACCATCCAAAGGTGCATGCCACACCCGGACCTTCCTAGGGGCCCGTGTCCCATATTCGGGACATTTGCCGCTTTTCTAGGGTAGTATCCTCAAAAATTCAGGATCTAGACCGAGGCCACATGCAATCATAGCAGGGAATGGGGTGACAGTCCCATGCTAAGCTCTTTGGTCCATATATGGGCCATGTGGTACCATACGACCCAATGCTAAGCTCACGGGACCTGCCGCGGTGTTATCATACACCCATGGAAGGTGTGGTCAAACATCGAGAGCGGCACGTGTAAGCCTTCAATAGGTTGTGTGTAAACGGGACCAAATCGCATCCCTAAGCTATCCATCGAGAGGGGCACGATCCGGCCTGTGAAGGGAATGCATGGCACGTCCCTTCTTCCGCTTCAGAACTCAGTGCCGCAAGATGTGGGCCCTAGCCACCACGGCCCGTCTCGTGCGCCACACGCCAACTGGACCCACACCATGCATACACGCGTGGCCACGTAGACACCCACCGGTCAACCTTGACAATGTACACATGACGCATGCGCACTCATTAGCCATCTAGCCCCAAGCCATGCACACCTAGCCACATGAACTCAAACCGTGCACCGTTAGCCAAGTTGACCCACACCATCCATCCACACTTGATCCCACCTCGGTTCGTTGGACCTCCGACGAGTCTGTTCCCATGCCACGCATGTGAAAGCGACATGCGGTTACACATATTGCACACCGAGATCCGACGTCATTCACTAGTGCCAGCAAACGTGTCCACCTGAGCATCTCGGCACATGGTCGACGCTAGCGCATGGTTTGGATCAAGAAGTTTAGCGGCGCATGTTGTCGGCCCAGTTGGCTATGGGAGCAGGATATGGGTCCAGATGAAACGCATGGCATCGCGTGGATCCACATTCCCCTCCCCGGTACCGGACAACGGGCATATGGCAGACGAATCCGTGCGGTCGCCGACGGAGCTTGGATGCCATGTGTTAGGTGCATGATAAAAC

>HchH1Sat25-44

AAAATTATTTGTACTAGGTTATAGAGCTAGTTCAACTCTAGTGT
