# Supplementary material for: Highly divergent satellitomes of two barley species of agronomic importance, Hordeum chilense and H. vulgare
Source: Plant Mol Biol. 2024 Oct 2;114(5):108. doi: 10.1007/s11103-024-01501-5 (PMC11447152; doi:10.1007/s11103-024-01501-5)
Supplement: Supplementary file 3 — Supplementary file3 (DOCX 17 KB) [file 11103_2024_1501_MOESM3_ESM.docx]

Additional file. Sequence in fasta format for each satDNA family of *Hordeum chilense* (H7).

>HchH7Sat01-337

AGTAAATGCATGAAAAATAGCAAATGAAGTCAGAAAGGGTTGAAAATTGATGACGTGGCTTCGAATGGTGCATACTGAACACACAAAAAGTCTGGAGTTGAAATAAGTTTAAAAAAATGAAATGCCTTTGTAACAGACGAGTTTTCGTCTGAAACCCTGATACTTCGAAAGAGATTGTCCATTTTGTACACGAAGTGCATCCAGTTTTTGCCGTAACCCTCTCAACTTTTTAGCACATGCTATGTGGGTGAAATGATGATACCATGCCAACTTTCAACCTTTTCAGAGTTCATTTGTAGTGCTTTTCAATTTCAGGGTCATTTAGCTCAAAAAAATC

>HchH7Sat02-118

ACTGGCCAGAATAGGCCAAATCTGCGAGTTTTGACGAGTTCCCCGTAATCGGACCCCGGGGTTCCCCGAACGTTCGGATCGCAGCGGGACCCAAAATCAGTGAGTAATAGTATAGAAA

>HchH7Sat03-355

GGAATGACCCCCGGAGCACCGGAATGCCACCAAATCTTGCAAGCGGACCTAGGATATGTGTGAAAGTGTGTTGGAATGGTAGATTCACCAAATGGTGCAAGGCATGGCTCCCCGGTGTGACCTTCCTCACCTTCGGGCGATAACACTTAAGAGATTCCTCGACGTCTAGGGTGAAGTGTCCCGCTGCGGCTATACCGGAGGCTAGAATGTGCCAAAGGGTGCCACACCTGGATTTCCATGTGTCCATGGACTAAACAAACCTAAACCTATGAGAAAGTACATTGGTGGAACCTCGCGCGGAGAAATCTAGGGGGTAGACCACCCGGGGCCCACAGACAGCCGTTTCGGGGGGGGG

>HchH7Sat04-334

ATGCCGCGAGGTCTTGCGCCGGGCCCTCTTACACGTGTGGAAGAGTGGTGACGGGTGTAAGCACCCGGCACGCGGCTCCGTGGGGTGACCCCCCTCACCGACGTCGCTGCCGCCGTCACACGGAGGGTGGAAACGGCCACGTCGGGTAGGCACGGAGGGAATCTTGCGGTGGGTTGGGCCCGGACCTTGATCCGAAGTGTACCTATGGGCTGGCCCGTCACAGCCAGGTGGAAATGTCCCTCGGTCTAACCCCGTCCGGTGAAAGTCAAAGGGGTAGATCCGTCCGGTCAACGGTGTCAGGGTTTGACGGGACGGGGGACTTTGGGGGGTAGCT

>HchH7Sat05-118

TGGCCCGTTTCGTGGACTATTACTCACTGTTTTGGGGTCCCGGAGTGATTTCCACGATTGACGAACCCCGGGGTGCGTTTACGTGTCGGTCATCAACACTCGCAATTTTGGCCGATTC

>HchH7Sat06-46

GGCGAGACACGCCTTCGTCACTCCGCCGGACGCTCCATCGTCACTC

>HchH7Sat07-484

TTCGGTGTTCATTTGGCCTTTTCAAGACAACAAGTGAGTTTCTAGCCATGTAATGGCCATAATTCAAATTTGAAATACATGAACACGTCACGATGAGGCAAAAGTTGTTGGAAAATTCATATTTGTGTCCTTACTATGCCCCATGGTATAAATGGGAAGGAATTCCAAACATCTTGGAGTCACTCTTTGGACACAAACGTTAGGAATCTAGGTTTTAAAATTAGAAAATAAAAAATGCTCTGAAATACATGTAAATTTGCATGATTTCATGACATGGCACCAAGATGTCGTGGTAAAAATTATGGCTTTGTTTCAGATAAGCTTTGACACGTACATGTAACAAACCGGAGCATCGCATGAAGGCTAGCTCGTGGTTCTGAGAGGGAACGGGGGACACGGTTGATGACGAACCACTAGTCGCTGCCTCACTTGGCCTTGCTTTTTTTCACTTATAACGCACGAATTATGCTAAGATTTGCACATT

>HchH7Sat08-336

GAAAGTCAAAGGGCTAGATCTCGTGTTCAACGGGGCTAGGGTTAGTCCGGTCGGTGGGGCTTCCGGGGGTAGCAATGCCACAGAAACACCGCACCGGGCCCTCTTGCATGCGTGAAAGCGACGTGGCATGCGTAGACGCCCGTCTTGGGTCCCCGGGGTGTGCCCCCCCTCCACGGACGCAAGTGCCGCCGTCACCTGGAGGGGGGAATCGTGCGCGTCGGGTAGAACCGGAGGGAATCTAGTGGTTGGTTGGGTCCAGACCGTGTTCGGGGTTGTTCCTATGGGCTGACCTATCGCAACCAGGTGGAAGAGTCAACTGGTCAAAGCCCGTCCGGC

>HchH7Sat09-344

ATCCCAGGGACACATGCAACATTTGGAGGCATCCCGGCGCTCCGGTGCTCGGTTTCCGGCCAAACCCTAGCCCGTGGGCCCCGCAGATCTACCCCCCCTAGATTTCTTCGTACGAGGGTTGACCAATGGACTTTTATCTTCGTTCGGGTCAGGTTTGGTCACAGCAACTTGGTAGCACAACTTTGACCCCAAGATTCCACGGGTTTTCCCTCCGGTTGACCGTGGTGGGAGTTCTCCGCTACAGGTGCAGAACCTTCACAAGTGTCATCGCACGCTTCCGAACAGCCTCGCGCCCGGGGGGCTAGATATGGCAATTCTAAACATCCCACCACACTTTTGCGCAT

>HchH7Sat10-662

ATGATGAAATGCCTACCTGTGAAGCTAAAAATGATTTTTAGCAAAAATAACGGGCAAGCTATGGAGGACCCGCAGTTCAAATTCCAGCCGGTTCCAGCTGAATCGGTCGATGGTTGTCTGAATGGCCGGGAGTAGCTACGAGGGTCGGAAATGCATGATTTTTGGCGACCGTCCGTAAAATAGGGTCTACTTTGAGATAGACATGGAATGGCGCCGTTTGGAATGTCTCTATTTGTAGCCGCTTCACGAAAAACACGTGTTTTTGGCATTCAAAAAATGAAAAATGGTTTTTTTTGTGAAACAAGTTGGAACCTCTCTTTGGCAACATTGTTTGCCATCACAAGATGGAGGCATGCGCCAAATTTGGGCATATTATCACAAACTATTCAACGAATGTGGCCATATCATTGCTCGTTTGGCTTGAAAGCCATGAATCTTCGTTCATGGTAGGTCGTTTTTGAGAACACTTTTTCAAGAAAACATCGGTATTCCAAGTTTAATATTTTTACTAGTTGGTAGGGCACATTTGATGATGTGACGCGGAGGTCTCCCTTTTTTTGAATTTTTTTGAATTTTTTACGGTGTTTTCAAAAACCGGTCGAACTTGTCGCGTCGGCCGTCCGTGGCTAGGGGTTTGATCATGCATATCTGTTGGTGATTCT

>HchH7Sat11-505

TCGCCATGTTTTCAAGGGGAACCCATATTTCCTTGGTCCTTTCAACTTGATTTATTTTCTACGATCAACATACCCACTAGAATGCACCATGTCCAAATTTCACAATTTTCAGGCTCGTTTATCGTATTTGGTCCCTTTTGTGTACTTTCTAGGTCTTTTGTGCTTATAATTCAGGTTAGAACGTTCTGTACATGAAAAGGCGTTCAAAAAGGACTCAAAAATTCGTAATTGTGTCGTTGAATAACTTTTCATGTATTTTATAGGAAACTAATAGAATTTTAAAGGATATGTGTGGGAAGAGGCAACCAGAGATGTGTCGTTTATTTGTTTTGTAACTCTAGAAAAATGGAAAACATGCCTGAAGAACATGTTATCTCGTGTGGTGCCTAGGCATGACCTCAATATGATTCGGTAAAAATTTGAGGAGGTATGGATAATGTCGTGATAGACACTCTTCATATACCGGACCATCACCTATGGAGTTTCATGGTTCGGGGAGCGAAAA

>HchH7Sat12-728

GGTATTCACATCATGCCACGGTGACACACCCATTTTCGTGATTTTCGGAATACATCTGCATTTCCTCCAATTCAATTACTAACAAGCCCCATGTTGGTGGAAAAAATGTCTTAAAATTAGCAAACAAGTCATGAAATGCGGCCGAAAATCAATACAGGGGTTAATAGGTGATTGTCTGCGGATGAAAATAATTCGAGGCGAAATGATGAAGTCACAAATTCATTTGAAATGAAAATATGACTGTTGTGCATAAACAAATAGCACACGGTTGGTTCACTAAAACCGTGTGCTTTCTAAACCGCGGCCTATGAAACCTAATGCGCGCAAAGTGAGTCGTGCGTTCTGATTGGCCAAAACCCAAACCACACGGATCGTACGTCTGCACCGTAGGATTCACCCAGATCGAACGGCGGTCCTTATACCTCTCGTCAACACACGTTGTTGAAGTTGGGATTTGATTTATATGCAATAAATGCCCCGTAAATGATAAAAAATGCTGAAAAATATAGCAGGCGAGCCCAGAAATGCGCCAGAAAATCAAACCTGGCGTATAATTATGATTGACTACCATGGGATAAAATTTGGAGGAGAGGCGATGAAGACACCGTCGATTGGAGTTCGATTTGCCACTTCTCCCTTTGGAACTATGATCCTTCGTGTGAGATGCCTTGGTTTTCAAAGGGCCGTACATCACAACTTCCGCCAGACATTACCAATTTTTTCCATGG

>HchH7Sat13-320

GACATGGTGCCAAGTTTTCGTAAACTTTCATGCATGCAATCACCAACACCATGTGTAACCAGCACTGTTCGGGTCTACCGGAGGCCACCTCCGAAGGACGTTTTTGTTTTCAACTCCACAGAATGACCCAAAACTTTTTCCACACCCAGGCATGGCATTCACAAGCATGCATGCCAATTTACAACAATTTTCCACGTTGCTTGCATTTTCTAGGGTTTTCGAGAGGAAAATACCCGAAAACAACGCCCGGACGTGACGCAACGTGCGTTTTGTGTCCGGATTCGTCGAAATCTTGCGTGGGGCCCTTCTTCGGTCATGCA

>HchH7Sat14-2790

GGGGGGGAGGATGATATGGGCACGACGGCGGAGGTGCAGCCCAATTTCAGGACTCCACCAAGTTAGAATAATTTGTATCAGTTAAATTTTAATAATCAGTTTTAATGAATATTAGAGTTGAGTTTGGGCCTGTCCAACTCAGCTGCGTGGGGCCTCTAGGGCTGGCGCCCCACCAGGGATGGCGCCCCACTAGGGGAGGCGCCCCAGCCCTCTCATATACCCCTTGGGGCGCTGCACTATAGAGATTAGATTGAGATTAGTTTTCAGATTGCAGATTACTGCTTGTGTGCTTGGTGAAGCATCCCTCCGGGGACGGCGTAGCCGTGTATCAGATTTTAATACAGATTGCTCGGAGGTTCTTGTGTTCATCAAGGATTGTCGTGCTCTTGGTTTGAGGCGCGGTGAAATTCATCGTGTTGCTTGCTGGATTCATCCTCTTCTTCAGGTTTGCGTTCATCGCGTAATTGGGAGAATTTCTATCTGTGGTATTCGCTGTGAAAGATCGGGCAACAATCCTAGGATCGATCCTCTTGATCCTCATCAGTGGGTTGATCCTTTATCATGTGGTATCAGATTACTAGGTTGATCACAGCGAGGTTGTGTTGATCGATCTTACCTAGATCGGTTGACTGGGAGGCAGATTGTGCAGATCGGTTGATCTGTTGGAGGAGATTGATTCTTGCTAGATTGATTTGTGTGGAGGCTGCTCGAGGATTCAAGGAAGGAGAACTGAGTATTGCTGCGTGGATCAGATTGAACTCAGATTGGAATCACCAGGAAAGGCAAGTGTGATCCGGATTGCTTATTGTGCAGAATCTGCGGATTGTCAGATCTATTCTGGGCGTGACTTGTCCTGGACACAGAGGTTGGTAGGAGTCGAACCAGGAGCTGTGCGAGTACTCATTGGAAAGGGCACCAAATTCTCTTTCCAAAGAGTCCTTATTTGCTACGTTTGGATCTCGTATGAAGGAGCTAGGTCCGTTTTAAATTCCGGACTTCTGTCAGTCCGAACCGAATTTCTGCAATCGTGTTGGCCCAGGACTCTTCTTCTTGGTATTTTGCAGTTCGGTTTTGGAGTCTATCTCTGTGCCTATAGCTGAATCTTTGGTGCACTAGCAGCATTACAGTCCATTGATACAAGTTGGTTCAAGGTTGGCCACCGTTTCCTTGGTGCAAATCGTGTGGGCAGTGGACTTTTGTGCAGAACATATTGGCTGCAGATTGCAGATTGCAAATCTTGATCAAGCAATGCAGCAGCACATGGCAAAGGTTACTTTGAATTTAGGATTGGAGTGTGTTCCTTCTGCACATGATGTCTATCAAGATTACTTAGACAGGCTGAACGGTGTCCCTTGGGATCCTACTAAGCTGAAGTGGAGACTTGTACTCACTGCATCACCGACAGAATATAAGTTATGGGAAGATGCAATGGAGCGTGGTTTTGAGTTGTGTCATCTCAAGCCTACTGATATAAGGATTCCTACACTTGTCCTGGGGCGCATTCATTCCCGAGTCTACAGTTGGTGGGGTACTGAAACGCACTATGAAAGAGCAGGTTCTTGGGCAGATATTCGCAAGCTATTCCGTGCTAAATTTCTGCCACCACCTAAACTAGAAACTCAGCAGCCTGATGTTGCAAAGGTTGTTGTTTATGCTGAAACTGCAGTGGCAGAGGCTGAACCTTTGAGTGGGCTGAATATGCAACTCACGAAGGTTCCTGTTGCAGCTTGCAAAATAGTTGACAAGGGTCAGCGATGGAGTTTATTTCAGACTCAGTGCATAATAAAAGGCAAAGCGTGCAAGCTGATGATTGATGGCGGAAGCTGTACAAATGGCATAAGCAAGGCATTGGTGGCATCATTGGGATTGTCTACTTGGCGTATTCCTGAACCTAAGCATATTCAGTGGTTGAATAGCTGCGGTATGCTGAAGGTTACACACAAGGTACGTGTGCCATTTACAGTTGGTGATTATGTTGATGAGGTTGAGTGCGATGTATTGCCATTGGAGGTGTGTGGATTGTTACTTGGCCGTCCATGGCAGTATGATCGTAATGTTACACATGCTGGGAGAGCAAATATATATTCTTTTGTGCATGATGGTAAGCAGCGGACTTTGAGGCCTATGAGCGATGATCAAATCAAGTCCGATGTGGTGCTGGTGATCCAGAAAGAGAGGTTGCGCAAGGCTGAACCACCTCGGTTAGCTAAGTTGCAGCAAGAGGAGCATGATGCACGGAGCGTTCCTGTTGATATTACTTCAGCTATGCCTGTAGATGACAAGCCAGTACTTGTTAGTGACACGCCAGTTGAAGTACAGCCTCTTACTGATGAGAGGAAGGATGTTGCAGCATGTGTTACAAAGCCAGTTTGTGTTGACACAGGTGTTCAGACTGATGAGAGTTGTGCTGATCATGTCTCGGTGCATAGGGTGCCGCGGGTGGATGATCGTCGCAGTTATATGAGCACACCTGTGAGGCGTTTTGCTGGAGCAGCAGTACGTATGCATACAGGCAAAGATGGACGTGTTCGTCAGCTTTGTGGACCAGGCATTACTCATATTTTGCAGGGGCGTGCAAAGCAGGTTCATGTTCAGCAATACAAGGTTCCAGCAAAGATGGAGAAGAAGACGAAGATGGTGGCGCCTATGCCCAGGCGTGTGTGGAGAAGAAAGGCGCCCACTGCTGAAGCAAATCAAGACTTGAAGATGGCGAAGATGCGTGATGCTGTTGTTCATATCACGCCACCTTTTTCAGCAGACCCTCAAGCGTTGGGGACAACGCTTCTTGAA

>HchH7Sat15-44

TTCAACTCTAGTGTAAAATTATTTGTACTAGGTTATAGAGCTAG

>HchH7Sat16-503

GTGGGCCTCGCCCGAGGCCGCTTGTAAACTGCACCATCTTCGAGGTAGTCTCTTGGTATCGAGAGGGAACGTGTCAGGTTTGTGAAGGAAGTGTTGGTCATGTTGCTCCGTTGGCATCGAAACTCCTCGTGCTCTCAAAGGAGGCCATCGAATGACACGTGTCAGGCCCCCGCATTTTTCTGACCCGCCTACAATATTTGAGACATTTATCGCACTTCTAGGGTTTCAGCGTCGGAAATTGCAGATCTGCAAGGCGACACATGAAATCATGCCCAGAAGCAGCATGGAAAATCCTATGTGATGTATTTGTGACATGGGTATGCCTTGTGCAGGCGTGGGAGGGGCAGGCCCAGCCATCGGGGGTGCGAAATCACCTGGGCGACATGCCTGATCCAACCGTTTGAAGCCTCGGAAAATGGTACGATGCCAGAAATCCTCAGGATCAGGCACGGCGTCATCACATGGCCCCTGTAGGGTGTGGTAAAAGTTTGGGCGCGTTCCCG

>HchH7Sat17-1262

TTAGTGTCATTGGCCAGGCCTATGCCGACGGCCCCCGTTTGCCCCGTCGGCGTAGGCCTCACACCGTCAAACGGCCGTCGCCGCCCGGGTAGCCTGGCCCACCTCTATGCCGACGGCTTGACCTATGCCGACGGCCACCGTAGGCATAGAGCGAGCGTTGCCGACAGCCGATCTATGCCGACGGCCCCGTCGGCATAGGTGGACATATGCCGACGGCCTGTCTACGCCTACGGCTCGACCTAGGCCGTCGGGATACATACATCTATGCCGACGGGGGCCGTCGGCATAGATTAGGCCGTCGGCAGCTGCAGTTATTCTGGTAGTGACTGAATCATTTTTCGCACAAGGAATGTCATGATATACCTGTGCCTTGAACACCTCTGGCCCCAGCCAAATAAAGAAAGACAAACTGGCCAGTTCCCCTATTGCTGAAGCGTCACACTCGCCCCTAAAACGTAGTCTTGTATCTCCCTCAGGAGATGCAAAGATCATGCCGTTGTCGTGAAACAGGGGGGATGAAGCAATTCCAAGACCTGTGCTCCAATCTTCTACAACAAGTACCTTTATGCGACAGAGATTATCCAGGTAACTGGACCAGTGGACTTCGGACATGTTATGCAGTATAACCATAAGATCAAGTGTAGTCATATACTTCGGAAATCCACCAATTCTGTCCTTGTAGGACTGATTGAGATCAATAGCATCAATTCCAGTGAGTCCGAATGGAGAAAGTAATCCGCGCAGCTTTATGGCAGATTCAACCTTATTATGGTACAGTTCCTGTAAGATGGGAACAGAGACGTCCCTTTCGGAGCTCTTCAGATCTCTTTCCCTCTCTTTCACAAAATCTTGCCAATGCCCTATGCCAACATCAACTGTTTGGTTGTTGAAGCTGAAGGTAGTCTGGAGGCTGCCATCAGATCAATTGCAGGAAGAGAACTTGGACTGAATGGGTCATCTGCACTTGCTGAGAAGTTCCTTGGAGACACCAGGGATGATCTGCATAAGTGGTAACCGACAGATATTCAGTACAAATGTTTTGCTGGAACTTCCAAGACAAAAGCATGGACATTTATGCATGCTATTTATTCATTTGGATGGGTTCCCATCATCCTAACTTCTTCATCAAAGGGACAATTACCGAACTATTGACGGGATCCGGATCAATCACCTGATCGATGACGTGGCATAAGATGTGGATCGATGACGTGGCAGAGGATCTATGCCGACGGCTTTGCCGTCGGCGTAGTTTTTTTATTTTT

>HchH7Sat18-46

GCGCGGGGCGTCCGAGTGATGTAGACGCATCCCGCGGAGTGGCGAC

>HchH7Sat19-518

AACCCCAAAAAATGAAGTTTTGCATGCTCTATCACCCATCCCGCTGCCATCCGCATGTTTGGGCAAGAACATGCGTCGTTCTTGGACGAAAATGTGCATAGCAGCTCTTCTGCGCTCTTATCATTTACACGCAAAATAATAGGTCATTCTGAGTGGTGAAAGATGGCTCCCCGACGCGATCCACTTACCTTGGAAGCTCGACTCACGGTCTGCCCCGGTGCTGAAATCCAATTAGGTTGTATCCGTAGAACGGGTGTAATCTGGTCATACGAACTCCGTTTGAGCTCCACGACCTGCCAAAACGACCGCAACGAAAAGTCGTATACCCCAGTGGCCCACTTCGTCGGCTTCAGCAATGTTTCGGAGGCCAAAAATGGCTTGCAAAATTGAGTTCCTGATGATTTAATCGCTGGCGTGCACACTCCTAGCCTCTACCATCGTTCCGTGAGCTCACCCACGGACACATATTGGGACAATAACATATAGAATAATGCGATATGATTCTATTTGTGCACCAA

>HchH7Sat20-320

TGCGTCACGTCCGGCATCTGTTTTTGCCTGTTTTTGCATGGAAATTCACAGAAAAGGCACGGATTGTCAGAAAATAAAAAAAATTGACGTGCCTCCTTGTGAAGTTCATTAGGAAGCGTGGAAAAAAATTGGGTCCGTTTGATGAAGGGGAGTAGCAAGATCGCATTGTAACACCCCTTCCGCCCATACCGACACCGGTGGGTTGCACATGATGTACATGCATGCATGCATGAAATGTACCCAACTTTTGCCAGCAGTTGAGAATGCACATCCAATTGTGCCACGCAAAATTTGAACGAAATCGAAGACCAAACGCACGT

>HchH7Sat21-118

CTATAGCACACTGTTTTGGGGTCCGGGGACGATTTTCAGGGCCCGTGACCCCCGGTACACGGTTTCAGGGGCGTCGTCAAAACTCGTCGTTTTCGTGTTTTCTGGCCGTTTTCGTGGG

>HchH7Sat22-364

GAAATATTTTCGGCTGCATTTCGCCGCCTTCTGCCCCCGTTTAGGAACCAAACCGCGCAGTTGGATGTTGGGTTGGCTTCCATCCCAAGGTGCACCCCCTATCGAAGATGCTAGGCAACATCTGGCACGTCAACTGTGTGGATAGGCTATTGGTCACGCCCTAACTTGTGGTCTGGGGAGACATGTGGCGAGCGTACAACTTCATTCTCTTGATGGGATGTGTATTTCTACTATGCATCTTCTTCTAATTTCAGATTCTCTAATTTCACCTTGGATGGTGTAGCTACTATGATTCTGCACCATTTGGAACAAACCGCGCTCAAAACAGGGCCCAGATGAGGAAATTATTGCATTTTCAAGTTTG

>HchH7Sat23-692

GGTGGTCGCCGGAGAGGAGGAGGAGGCCACCGGAGAGGAGTGGGAGGAGGTCGCCGGAGAGAAGGCCGCCGGAAAGGAGGAGGGCAGTATGGTGGAGGAGAGGAGGAGATGGAGTGGAGTGGAGGAGATGAGGAGAAGATGGAGTGGAGGAGAGGAGGAGAAAAATGAAGAGGTAAGGATGAGAGGAGGTCGGCCAGCCAATATATACGGCATAGTAATGGCGCACCTATACAGGAGTGCGCCATTACTAACTTTTTTTTTCCAAATATTTTTGCCTCCAAATCTTAAAAGCACCGTAACTTTTATTCTGTTAGGTTTTTGAGGATTCTAAAAATGTTCAACGGGGTTCCCTTATGAGATAACTTGGGTTTCTTGGCAACACGGGCCCCTTCAGGTTCAACTTGCCACATGTTCACTGACGTAGCAAGTGTTGAGGGTGTGCCGGCATCCCAATCCACCTGCCAATAAACAAACACAATACACTACTGCTGAATCAAGCTTTTTCATGAAAATCCATAGCAAATACTGCAACAACCGTAGTTAGTACAACTTGTAAGAAGAGTCTGAACACATCTGAACCTGAAACTACATCTGAACACATAGCAAAACTACATCTGAACCTGAACACATCTGAACACATCTGAACCTGAAACACAATAACTTCACCAGAGCCTGGAACGGATTGAGGAGGA

>HchH7Sat24-245

CTGGCGTCAACCGCATCCCGAGCCATCCGATCAAGCTAGATTGTTTTGATGTCCAAGAGTTTCTCGCAATTGCTCATTTGTTTCATGATATTTAAGATTTTGTTGCGTGAAGTTTCACAAACACTGGGATATTCACTGTCCTTTGATCTTATTGGTGGAATGGGGTATTCACCAACATGGGAGGAAACTCACACAAACTAATGGGATGGGTCAAAAACTCTTTGTTTCCACCGATTGATCTTTGG

>HchH7Sat25-82

CCTTAAGAAACTTCTTACGAAAATGCGTCGGAAGAAACTTTTCTTAAGAAACTTCTTAAGAAAGTGCTTCCTAAGAAAATTT

>HchH7Sat26-4341

TATGTACCATGGCGCTTTGAAAACATAGAGGTTGAAGTGCATATTATATTGATGTATATGTTGATATTAAGTGTCAGTTAAACTAATAAGTACAAACCGCCCAACATCTCAATATATAAATTAGTTGGTCAAGTGCACCAACATTAATATTTCATTAGAGTTGGTTTAGCTGGTGAAGGTCAAGTACTCACCTTCTAAGATATGCACACCAACGAGTAAAAAGCTTATAACGTTGGCTTCAATGGTGGACACACCTTTAAAGTTTTGAGAGCTTAACTGCCACTGAAATACTCCAATGAGTTGTATTACAACATGTGTGTTTAACTGATGCTAACAAAATCCCTATCTTTGAAGATTTTTTGTTGTTCTTTGTTTGGAATTTTACCATTTTGATAATTTAACTTCAAGCGACTTTAGAATAACCCATTTTGACTGAGATTAACTATTGTGCACACCAAATTTAATGGATTCATTTGATCAAGTTGCTAACTTTTGAATCCCCCTCCTCCGACGTATTAGCGTGGTCTCTACAAAGTTTGATAACACCTAATTACTCCAAGTTTCCTTCCTCCTCGTGAAACCTGCTGGGACTATATGTGTCCTCAGGTTTCAAGGTAAGCCAAGAGGACCATGACTGCCTTTTGAAAAAAAAATTATGGGGCCTCCTTTTACCCATTTACCAATGAGCAACAAGATAAATCATTTCCTACACTCACAAAGGTCAATAGTAACATCCGAGAGTTTTCATTTAATCATCAAACCCACATTGATGCCTTGATGCCTATAGTGAAGGAGTGGACCAGGGTGCCTAGGTGCTGCCTTTGCTATCACGGCTAGTCCCGAGGCATACGGAAGGAAATGGAACATGTAACCATGCAGCCATCCATGGCGAGGGTGTTTATTAGTTGATAAAGCACAAGTTGTCACACTAGGCGTTGAGATGGATTGTGATGGCCATGGGCAAAGTCATCCAACAGATGCAGCACACCAACAATTTTTAGATGGGTCGACAATGCAATTAGATTTTATATTCAGTCTTTAATTTGTTTACGTATATACATACAAAATTTCCAAACATGAAGCAATATTCAACACTCCCCTGTAGTTTTTATTCTAAGCAATGGCATGTGGCAGGGAGCATATGGTGATCTACAAGTCTCAATTATTGTTGCCCTAAGAGTTGGTCACATTGCAATCTAGCACCATTGTTCGGAGTATTACTGGTTCGCATATTTTGTTTATGTTTCAATTCACCATTTTTTACATGGTGTAAAATAGGGCACCTCAACAATGACACTAAATCTGTCAAATTTGCATGCATTTATTACAGAAAAAAGCATGCACGCACGCACACACGCATAATTCAACGTCCAAAATATGGCCGTCTGACCTAGTTAATGCCTAATGGCCTTCCGTTATAAACAAGAATTGCGGAAAGGCATTGCTTGGATTGGCGTGCGCGATCATACACCACCCATTCCAATTGCTAAATTTGGTGCAAAGGTATCTATATAAGAAACAAGAATAGAATGGGATGAGGAAGGAGAGGGGATCATCGCATCAATCATGGGGGTGATGAGTAAACAGGCATTCCTATTGTTTCTTCTCCTGGTGTTGCTCAAGCTCGGGTCGCTGCCGTTACCATTGTGCCAGGAGGTCAGTTTTGGTGAGTGGTCGAAGAGTGTGCCAACTAACGTAGGCAAAGATGCAGGGTGCGCGGCGGAGGATGCTGCACTCTCATCCGTCGATTCTACGTTTAAGGTAAACAATTTTATTGACCCTACAACTCAAAGGAACCCAGAGGATGGAGAATACAAGACAATAAGCGAGTCCATCGCCAACATCCCCGATGGTAGCACAAAACGCTACATCATCACTCTCAAGGCTGGCACCGTGTTTCGTGAGAAGGTGTTCCTTAATAAAAGCAAGCCATTCGTCACTATAAGGTCCGATGACCCCAACAATCCCGCCATCATCGTCTGGAATGACACCGCGACCACCCCAGACAAGAATGGCAAGCCCCTTGGTGTGGATGGCAGTAGCACTGTGACAATAGAGTCTGACTACTTCGTGGCCTATGGCGTCGTATTCAAGAATGATGCACCGCTACCGAAGCCAAGGGAAAACAAAGGAGAGGCCCCAGCATTGCGAGTGCTAGGAACAAAGGCTACTTTCTATAACTGCACCATTGAAGGCGGCCAAGGCTCCTTGTATGACCAAAAGGGGCTGCACTACTTCAAGTCATGCACCATCAAGGGCACTATCGACTTCATATTTGGCTTTGCCAAATCATTTTACGAGGATTGCTACATCCTTGCCATGAACAAGGAAGCCTTCGATTTGCCAATGGTGCCGTTGCAAGATCATGTAGATAAGAAAGGCTTTTTTATAACATATCCCATCAAGGCTGCCCCAGGTGAGGGTGGGTTCTCTTTCAAGACATGCACCATCAAGGGGGACGGAGAATCTATCTACTTGGGCCGGCTAGGCTCACCTATCATCTACTCCTTCACTGAGATCGACAAGAAGATTGTGCCGATACTCTCCGATGAACGGAACGTCCTAAAGACTGAGAGGTACAAATCATCTACAACTTGTACATGTTCCTTCCTACATTTATTCTTGACGTTTGGATTTTGTTCTTGAATTTTTTTCATCCATGAGCGAAATCAATGAAGTTCCTGAAATTTGGGGCGAGGTTTCACCAAAAAAATTTAGGGCCTTCACCAAAGTATATGATTTTCTAAAAGCATTATTGAAAGAATGATTATCATGTTTAGGGCTGTACACCTCTTGAATAGAGGAATATATGACACAAAATTGTGAAAATGAGCATAGAGTAATATGGTACTATATTACTAACTTTTGGAATTTTCTTTCTCTCACCACAAGAATGAACGAAATTCAAGAAATGTCTGTGAAAATTCACTGAAATTTGTACCTCTGCCTTTATCAGTAAAATGAAACACTCTTGATCCTCAATATACACGCAATGATGTCTATTTGTTAATGTTGTGCTACATTCTTAATTTGCTGTAGAGGGATCTTCAGCGCCACATTCAAGTGCTATGGACCTGGGGTTGCTGCATCGACAACCTACGCTATGGAATACGCTCAGGCCAAACCATTTATCGGCACACACTATATCTCAGGAGACTCGTGGATCCCACTCCTACCACCCGTTGAGTAAAGGAGAACCATGCATTGACTAGGTGTCCCAAAGTTACCTAAGTCCAAGTTGTACCACCTGTTTTTGTTACAATACCTATGTTTGATATTTAAATAAACCATGCAGGACCTGTAAGCATGCTTGCAAATTGAAAGCAATATAATCAAAACTACATTGATCCGCCACTACTATGATTCGATGATATGTATGGGGATACATTCATTTTAGAATCCTACAAGTTAGATAAACTATTTCATCCTGAACTCTTGGATTTTTATCCATTTGATATGCTATTCTTAGTTATATATTGATATAACTAATGTGGCCAACACCCATCATAAAAAGTTACCAATCACCGACAGTTAGAACATTGAGGATGTGCATATGTAGAGAGCAGTAGACCACCCCTACATGTGGATCACCTAAAAGGTTCCACACATATCCATAGCCATAGTGCGATGAATCATTGTCGTTTTAGTGTGCATACATATACACTTGCGTTGAGAAAGAGATCCTCTGCTGTACGCACTGGTTGCAGACATGTACGTCACTGACCCAACTGCAAGGGCAGTACTGCAAAGGATCACCACATTGGTTTGCAGGTACTATGCAGATAATTTTTCTTTATACATGTACACATCTATGCCGCTTGGCCGCTACCCTGCTGCATATGCTCTCAGCCGTCGCATGGGTTCAATTCATGGACCGGAACTTGGAGCTGGACGGCAGTGCTGCCTTCCAACTTAATTATTACAGTATATAAAATGAGAGACCGTACATCCACTTATACTATTCTCACTCGAAGTTTCATTCTCCACAAAATGAGGTGCCACAATAGTAAATATGGTGACATGGCGATGGAGATACGGGGAGAGAAAATGAGTTTATCGTTTTCAATTTGGAAACTTCAATTTAATTAATCTTTGTTTCTATTTTGGTCAAGTTGGAGGCAAGAGCTTGAATTGCTGAAGATGTTTGAAGGCCTGTCGTATGCATGTTCTTGTACTACTTCCAATGGCTTATCAAATTGTGCCACTTGAGAGAGAACGGGCAAAAAAAACAAGATGCGTGTCTACAAGACATCGCCGTGTGGTGAAGCAAAAAGTTTACAAGCAGGAAGGGAACTATGGATAATGGAGAGAGAATGTGCATGCAGTTGTAGTTATCTGATG

>HchH7Sat27-410

CAGCTGCGGATGTTAAAGTCCTGCAGACCAATCTACTGAGTTCCAGTCCCTGACACGGCTAGGCCTGATCTGTGGGGCGACATGGTCATGTCTATCACAACATTGAAGAAGAATGACGCCAAAGACCGGACGGAGGACTACAAAGATTATGTTGCCTTTCTGGTAGACCTCATGTTCTCAGGGTTCGCTGGCAAAGCATCTTATGCCCCCCTTACTCGGGGGCAGCAATCTTCTAGCAGATGCAACTCAAACCAGTTGGGTGCTTTTGAAGGTAATTCAGTCTTATCCTGCCCTGATGCCATTGCAGAACAAGGCTGTGTTCTACCGCCGTGTGTACAACATTGTGCATGAGAGGATGGAACTGGTCGTTCGCCACAACCTGCCTGCGGCGGGCTTGGTGGCCGACGGTA

>HchH7Sat28-28

CCGGTAGCCTCCTCCCGGCTGGCTACTC
