# Supplementary material for: Highly divergent satellitomes of two barley species of agronomic importance, Hordeum chilense and H. vulgare
Source: Plant Mol Biol. 2024 Oct 2;114(5):108. doi: 10.1007/s11103-024-01501-5 (PMC11447152; doi:10.1007/s11103-024-01501-5)
Supplement: Supplementary file 4 — Supplementary file4 (DOCX 18 KB) [file 11103_2024_1501_MOESM4_ESM.docx]

Additional file. Sequence in fasta format for each satDNA family of *Hordeum vulgare* (H106).

>HvuSat01-338

AAGTAAATGCACGAAAAATACCAAATGAAGTCAGAAATTGTTGAAATTTTGTGATGTGCCTTTGAATGGTGCATTTTGAACACACAAAAAGTATGGAGTTCAAATAAGTTCAAAAAAATGAAATCCCTTTGTAAGAGATGAGTTCTCGTTCGAAACCCTGATACTTCGAGAGAGATTGTCCGTTTTGTACACGAAGTGCATCCAGTTTTTGTCGTAGCCCTCTCAACTTTTTAACACATGCTATGTGGGTGAAATGATGATAGCATGCCAACTTTCAACATTTTCAGAGTTCATTTGTAGTGCTTTTCAATTTCAGGGTCAACTAGCTCAAAAAAAAT

>HvuSat02-444

AAGAAGGAGAAGGAGCTCCTCCTTCTCCTTCTTCTTCCTCCTTCTTCCTCCTTCTCCTTCTCCTTCTTCTTTTCTTCTTCTTCTCCTCTTCCTTCTCCTTCTCCTCCTCCTCCTCCTCCTCCTTCTTTTACTTCTTCTTCTCTTTCTCTTCTTCTACGTAGCTTAGACTCATCCAAGAAAGGCTAAATTGGCTAATTATCCTACGAAATGACATAATTAGCTTAGTTAGCTCCAAAATGGCCTATTTAATAAAACATGACCTATACTTAGCTAATTATCCTCGAAATGACATAATTAGCTCCAAAAAGGCATTCTTAGCCAATTTAGCCCGAAGAAGGGGACAAGAGGAGAAGAAAGAGGAAAAATGAAAGGAAGGAAGAAGAAGAAGAGGAGAAGAAGAAGAAGGAGAGGGAGGAGGATAAGAAGAAGGAGAAGAAGGAGGAG

>HvuSat03-118

ACTCACTGATTTTGGGTCCCGCTGCGATCCGAACGTTTCGGGAACCCCGGGGTCCGGTTACGGGGAACTCGTCAAAACTCGCAGTTTTGGCCTATTCTGGCCAGTTTTGTATGCTATT

>HvuSat04-118

GCCAGAATAGACCAAATCTGCGATTTTTCACGAGGTCACCGTAACCGGACCCTGAAGTTCCCCGAACGTTTGTAGCGCCCCGGGACCCAGAATCAGTGAGTAATAGCATAGAAAACTG

>HvuSat05-5500

TGACAAGTCACTTTTGGAGACTAGGAAGAGATGATCTTCCCAAGATTCCAGGCCATAGAGCCACATAAAAGATAGTTCAACTAAATTCCAGGACTCAGTGGAAGCACAAGGAGGCATTTTATTCTACAACTAATTTCCACACATACCAATTCATTTCAAATATACACGCAATATGCAAGATATGTGTAGATACAACATGAAATCATAACATAACTGTACAACAAAATATATATTTAGAAGGCTCCGAAGAGCCAATGTAGTATAATGTTACAAGCAGGGGGTCACATGGCCCAACATTCGGACGATACAAACATCAAGCAGAAGTTACATGTGTGGGTACAGAAATCTACTATAAATGGATGGAAGAGCCTGGAAAGCTACAACGGCCCAACCAAAGGAGCCACACCACTATCTGGGTTACCAAGCTAATCCTTCGATGTCAATATCAATATGAAAACATAAACTAAACAACGTCTATCTCTGCAAAAATGTAAATAACCAACAGTTGAATCATGTTGTCCTAATGCATTGAAATGAGAGGAAGAGCGAGATAGTGGGATTGTATCGAAATGTTCAAGGGGGTTGCTTTCTTGACAGTTCCGAAGATAACCATGAGTCTTCAATAGTGTCAACGATCACAATGTCGGAACCATCACCTACCAACAGGGAACAACACCGGCAAACAGAAAAAGAACAAAATTTACTTCACGGAAATGCAACACTATGATGTATGAACATGACATGGCAATATGATGTTGTTTGCGCTAATGCATCTAGCAACAAGATTAAATGAAGTTGGTTCGAACCAAGGTTCAAATTCAAACTCCATATGTGATTATTTAAATGTCATTTATTTGAATGTCCTAAAAAGAGGACATACGTTGTTCTAACATGCATGAAAATGCCATAAACAGATTCATTGGAATTTTCAAAGTTTATAACATTTTCTGAAATGTAATATTGTGTTCATTTCTATTGTAATTTCTTAGACTCACATGTGACCCCGCTAGTCATTAAATCCATCCCAATTAACTTTCAACTGTAACAGGGTTGACTATTTGTGCCACATCATCCTCACATATGAACCCTCCTTCTCATAAACGCATTTAAAATGCATGAACTAGTCACTAACGGGAAGCCGTAGCTATTTGTAACTACTTATTCCAAAAGCGATAGATTTTTATTAAGTCATGTGAAGCATATTTACAAACTAACAAGATCATCAATGTATCATGAGAATCATCAAAATATATAACATAGTTCAAAATATAGGCCTAAGTTACATACGTAAAAATGAAATAGTAACTCACCTCGTAATAAACAGAAAGCAGTACTGAATTTTTGGCTTTCCTTTCAGCAGAGAGTCATTAACATGCAATGGTACAAATGAGATCGAGTGAAAAATATAATATTTATACCATGAAAAGCATCAAAACAAAAATCTACTAAAAAGATAAGAAAACATAAGGTATCGACTGAAAGAGTGGCATGAGTCAGTGTTAATAATTGATCCGAAACATTTTGGTTATCACGATAGAAAAGGTTAAGCATCTTGGTTGTCTTATGCTTAGATTATTTGTTCTTTGTTTAGATGTTGATGATGATGTACACATTAATATTGTTTTGGTTTTATGTACGCGAGAATAATCTATGTGTCTAAGTGTACGTTATTGTATGTCGCATTTTTTCATTATTATTTTCAATATGGTAAGGTCGTAAATAATTGGCCCCATCACCTAACCATACATGGGTGGTTGCTAGCCGGCCCCTTACATGCACGTATGTAGTACAACAAACAAGTGCTGGTTAATAAATGAAATATCATCCTTCGATCGGTAGGTACTATATGTCTATCATGTTACGACGCTATACAAGAGATGGTCCTAAACTAACTCAACACCCAAATGAAGTAATAGATTAATTGGTAGTATGCCAAAGAGGTCACACCAATCTGATTCATATTTTTTCACGCGTACATTGATGGGGATGCTAGAAATATACAATTAATGAACGGAAACTTGTTATGTTGAATCATGTAGTTTTGAATATATGGAGTGGTATGTGTCAATAAGTTAGTGGAATAAAACATCTATACCTACTATGAAAATAAATACGTACGTACGCATCGTTCCACTTGGTTCTACCGTCACGCTATAATTTTAAATATAGATGCGCGTGGCTGAAAAAAAATCGCTTGCGTGGTACTCGAATACACGAGCCCCCATGAGGGATGCCAAAGTAATGCTCGAGTGACCAAGTGATACATGACATGTTTTTGATTACAAGACATGATTTCATTGATACTATTAGAAGTGTAGGACGTATACCCTGATAAAAAGAAACACAAGGGACTCATTGAATTTCGCAGCTATAGTCCCATCTAGGTTGCATATAGGGAATCCCACCAATTCAAATATTCTTTGCAAGTTGTTTCGAACATCAAGAGGCCACTAAGGTAATCAAAGAATAGAAAAAAGAGAAGAGGAGATGCCTCGCAAGCCGCACCATTGAATTCAATTAACACCGAATCTTGATTACTACAAAGAAATAAACTCTTGATTGATGGATACTCGGGCATCCATGGATGGTGACTTGGCAATTAGATCGCAATAAAGGAGGGGGATTTGTAGGCTCTTGGACTTTCATAGAGAACTATTAAAGGGGAAGATGTTCTATCCCATCTAAAGGAAAATAAAGAAAACATGGACAACGTGTCCATGACTAGTTACAAAATACCAACACATTGCAATATGGATTTGATTAGAACCATTTAGAGCTTTGCCTACATCCATATATGTACAAGATGTTACATGCAAAATATTTTTGGATAATTACAATGGTGTTATAATGTTAATATATTTAGGTATGTTGTAACTTTTGGGACAACTGATATTTTCTTGTGCTAAAATTTCTATCCTTCTGATTCATATTAATCATTGCAGCTTTAGTACAACTTCAGTAATTAATATGTACTAGAGAGAGTATTATCTGAAACCATTTGGTGTTCATGATAGAAAAGGTTAGGCATCTTGGGTCTCTTATGGTTATATTTTTTGTTTGTTTTATACATGTTGGTGATGATGTACGCGATAATAATGTACGACAGAATATTCTATGTGACTATGTGTAATTTCTTGGCTCTCAATTTTTTTCATTAGTATTTTTTATTATCTAAGCACACAATTTATCGGCCCCAACACCTAAGCATGCATGTGCGGTTGTTAGACGACCCCTTTCATACACGTATGTAGTAGAACAAACAAGTGGTGGTTAACAAATCAAATATCATCGTACGATTGTTAGATAATGTTTATCTCTATGTTGTTACGATGCTATATACGAGTTGGTCCTAAACGAACCCAACAACCCAAATGAAATAACAGATTAATAGATAGTATGTTTCACAGGTTACACAAATCCAACTCATAATTTCTCGTGCATACATTGATGGGTATGCTCAAAAAATACAGTTAATGAGAAAAAACTTGTTATATTCGCTCATGCTGTTTTAAATATGTGCAGTCATATGTGTCGATAAGTCGGTTGAAGATAACATAAATAATAATCAAATTTGAGTCGATGGTAGAATCATAAAAATTATCTAGAGTACACCAAGTTTTAGGTGAAGTACTCTACATGGAACATATACTAGTAGTCATCCCAGATTCAATCAAACTTGTGCATTGATTCTATTCATTATGAATCTCATGAATCAAGGATAGAAGATAAGCACAAAGCTTCTTTAATCTACTTATTGATCCAACATGTACGATAGGTCATGCATTATAATAAGTGTTGTGACATGATCACTCTTTCCTCTAGGCCAGCCTGGTGTATGAGTGTGACGGGCATGCATTAAGGTCGTAAACAATGTTAATGACAATAATTGCACGTCCATATGAGATGCCTGCACCGCCATTACTAGTTAATTATTCGTGTTTGTGTGCTGGAAGTAAAACCAGACCACAATAATAAATAATTGTTCATTCAATACCTTCGTGCAATGTGGCATGTCTGGTGTCACCATGGAATACTTCCTATAAATAGAGATCATCCTAGACTAGATTCATCAGTTGGTTTTCTGATAAATACATACAAGGTGAATATGGAAACCATAAGTCTTAAGAGTATCATTATTGGAGTACTCATGCTAGGACTAGTCCTTCAACAGACCCATATAGAGGCCAAGAGTTGTTGTTGTTCCACCACGACAAGAAACTGCTATAATGTATGTCGTGTCACGGGTTCCTCTCGGCCTACATGTGCAAGCTTATGTGGTTGCAAGATTTTGGAAAAATGTGTGCCTCCATGTGACCGCTTCAACCTTGTCACGGACGCAGGTAAATTCTGAATCCATCCTTCTCGTTATCGTTCATATCATGAGTAAAAATTTCCATTTACATACATATTTGTTTTATAGACGAAGCAAAATCCATTGAGTTCTGCAAGTTGGAATGCATGTCCTCATTGTGCGGGAACATCAACGCTGGTAAGCTAAATACATTATGGGCCATTCGTTGCATATTATTATTCACTTCGAGTTAGTGATTGTTTCTAATGGAAACAATTCTCCACTCAGTTGTTGCGAGTCAAGAAGTGAACGATGTCAAAGATCATTGCAAAACTGGATGCTACCATCTCTGCACTAAGGATTATGAATTTGGCGAAGTCCTTGCTTAAGAAAGTGTGACAGTCGACATTTCATGTTGAATATACGGGAGTGTCAATGTTATATTGAATAACTGGATAACATGTTCAATCCAGTGTGTCAATTTATTTCTGCTTGTAAGTGTCTCGGTGATGCGATATTATTGATAATGTGCTCATCGTTTTGTAAGAAACGTATGTCATATCCAATATAATGTGATGTATTATTATAATGTGTGGTTGTGTGGTAAATGATAATTTAATTATTTTCTCCTTTTCCATTTATATTACTAGTCATCAACCAGTGCGACTGCACGACCTCGTCATTCATATTTCAAATCATTTGGAGGAGTGTTATTATTTGAGCATCAGAAGGTCATATCAAAATTTGAAATCATTAGGACATGCCTAGACAGTACCTCCTTCATAAAGTTCCTTTCTGGACAAACACTTTGGAAATTAGCTGAGAAATATTTAATAGGCAAATGATGCTGAATTTTGACATGATGCCCTAATATGGATAGGAAAGAGTCCACAAAATTTTTGACGGAAAACACGAATGTAGAAATGACACTTCCTTCACAAAGTGTTGTTCATGGCAGTATAGGAAAAGGCATATCCTTGTATTATTTTTGAACTAGACAAGGAAATATTTTGACATACGTGATGAATATATGACCCAAACAATTTACGAGAATTATTTGGGAATGTGGGAGAGACAAAAATATAGTTTGCTTCAAAACTAAGAGCAAATTGGATTTATTCCTTTATTAAAAAAAGGAATATTACCATCAAAAAGAATTTGGCTATGGGATAAGATGGAAATGACATGATATTGGGATGCATTTGAGAA

>HvuSat06-4925

ATGTGACACTCAAAGTGCTACCGCCTTGCCAAGAATTCAAGCTATCACTCTCGAACCAAGCACATTGATGTGAGGTATCATTGGATTCGAGATGTTGTGAGTTCCAAGTTGCTGAAACTTGAGAAGATCCATACCGACGACAATGGTTCGGATATGATGACCAAGATATTGCCAAATGAGAAGCTACAAGCATGTTGCAAGGTAGCGGGCATGGCGGTGCCCCCCTCATGAGTCGGGGGGAGATTTTTTGGGATATCCTCCTCATGTGGGTTGTGAGGAGATGACCATTTGAGGCCTTTTAGGCAGCCCAAAAGAGGTGCAGAAGCCCACTACCCATTAGGGTTATGACCTAGGGTCATTTTGGTCTTTGCACGTGAGTGGATGGGGATGCTTTACCCTCCATCCAGCAGCCACCACCTAGTGTGACGAAAATCAGTTCAGCCTCCATTGGAGAAGAAGAGAGAAAACCAAGTGAGAAAGGGAAGAAGAGAAAGATTGAAGGAAGAAGCAAAGGGAGCTCCTCCCCAAGGTTGTGATGATCCAGATCCACTACCTTGTCTCCTTCAAGCTGTGGTTCCACCATCTTTGGTGAGATTGTTCCAATCCCTAGCTCTTGAGCCCCAAATCTTGTTGTGTTCATCCAAGATTCAGAAATCTTGATGTATGAGATCCTCTAGTGCTATCTAGAGAAGAAACTTGTTGTATCCCACATTTGATAATAGTGGAAGAGGATTTGGGTGGCTTCGGCCCGTGGTTTTTCCCCTCAAGTTGAGGGGTTTTCCACGTAAAAATCTGGTGTCTCTTTTGTTGATGCTTGTTGCTGTCCAGAAAGTTACTCCTGCCACAAGACACTTGTCTGAGGAGCGTCTTGTCTGCTGAGTAACATTTTCTCCTCCATATATGTTGCTGCATATGTTTCCTTCCGTGCTAAGCAACGATCCTTGAGTTAGTACATGATGTGGTGCTGAGATTACTCTGTTTCCGTTGCAGTTTTCAGTTAACCACAAGTGCATTTGTGTGCTAATTCCCAACAGAGTGGCATCAGAGCCTTGGTTGCTTAGAAGGTTGCTAATCCCAGTTGCTTGATTGTTGCTGGAAGTGCTGCTCACAATGGCTTTGGAAGAAAGCGCTACTACAAGTGGCATGATAAAACTTGATTCTTCCAATTACTCATTATGGAAGCCCATGATGGAAGACATCCTTTATTGCAAGGATTTGTATGAGCCAATTGTGAAAGACAAGATACCCACAGGAGTCACGGAAGAAGAATGGAGAGTGTTGCACAGAAAGGCAGTAGGTATGATTCGGTTGTACATCAACCACAATATATTTCACCATGTTGCAAATGACACAAATGCATATGAGATGTGGCAGAAGTTGGAGTCTATGTATGAGAGGAAGACATCAATGAACAAGGCCTCGGTGATCAAAAGACTTGCAAAGCTTGAGTACCGAGATGGCACAAGTGTGATTGAGCACTTGAATGTCTTCCAATGCCATATAAATCAACTTTCAGCCATGAAGATCAACTTTGAGGATGAGGTGCAAGCATTGTTGCTGCTGAGTTCCATGCCTGATAGTTGGAACACGCTTGTTGTGTCACTTAGCAATTCAGCTCCGGATGGGAAGCTGACTTTGGAGATGGTGAAGAACAACATGCTGAATGAAGAAGCCAGAAAGAAGGAAAAGGGTGATGCCTCTTCTTCTCATGCGTATGTGGCTGAAACCCATGGGAAGAATGAGAACCGTGGACGTGGCCACACTAGATTTCAGCAAGGCATAAATATTTCCAGGGGAAGATCAAAGTGAGAACAGAGAAAAGATATTACTTGCTTTCATTGTGGCAATCCGAGACACATAAAGAGTGAGTGTAGGAAGTACAAAAGAGAGCTTGCAGAGGGGAAACTTGCAAAGAAAACTAAGCAAAAGGCTAAGAGCAGCTCAGCCATGACTGCAACAGATGAGGACTATCCAGTCATTGAAGATGATGAATGCTATAGTGTTGTTCATGATGATGCCATGAGTTGGGTTGTGGATTCAGGTGCATCCTTCCACATCACATCACACAAAGAGTATTTCACATCTTACACTAGTGGTGTCACTCGCCAAGTAAGGATGGGAAATAGTGGTTCGTCAACAATTGTTGGCAAGGGCTCTATATGCATTGAAACAAATACAGGTTGCAGGTTGGTGCTCGATGATGTGAGGCATGTTCCCGACATAAGGCTGAATTTATTGTCAGTAGGCAAGCTTGATGATATCAAACATCCTAGTTATTTTGGTGAAGGAAAGTGGAAGCTCACCAAAGGCTCTTTGATTGTGGCTCGAGGAAGTAGGCAAGGTAATCTCTATGTGACTAAAACCAAGTTGTGCAATGAGGTGTTGAACATTGCTGAAAAAGACACTTCGGTGGAATTATGGCACAAGAGGCTTGGGCATATGAGTGAGAAGGGCATGCATGCTCTTGCTCGCATGGAGTACCTCCCTGAGTTGAAAGGTATATCCTTGAAACCCTGTGCACATTGTTTTGCTGGCAAACAACATAGGGTTGCATTTCGTACACTCCCTCCACACCGTGCAGAAAATGTTCTTGATATCGTGCACACTGATGTTTGCTCCATGACTGAAAAATCTCATGGTGGAGCATTATATTTTGTGACTTTTATTGATGACCATTCCATAAAGGTTTTTGTGTATGTGCTGAAACACAAATACCAGACGCTTGAAGCCTTCAAGGAGTTCCATGCCAAAGTTGAGAGAGAAACTGGCAGGAAATTGAAGTGTGTGAGATCAGACAATGGTGGTGAATACCGAGGTCCTTTTGAAAGGTATTGCAGGAAGTTTGGCATCAGGCTTGAGAAGAGTCCACCAAAGACACCTCAGCTCAATGGTCTTGCAGAGAGAATGAACAGGACACTCACAGAGAGGGTCACAGCTATGCTCTCTCATGCTCATTTACCTAATTCTTTTTGGGATGAAGCATTGATGAATGCTATGTATGTGGTTAACCTATCTCCCTCAGTTCCTCTTGCAGGTGATATTCCTCAGAGAGTTTGGTCAGGGAAGGAGGTATCATACAAGCACCTGAAGGTCTTTGGTTGCAGGGCATTTGTACATGTCCCAAGGGACGAGAGATCCAAGCTTGATAGCAAGACAAAGCAGTGCATCTACCTCAGCCAACCAAGTGAAGAGTTCGGCTACAGGTTGTGGGATCCAGCCAACAGAAAAATTGTGAGAAGCCGGGATGTGGTGTTCATTGAAGATGAGACAATAAAAGATATTGGGAAACCAGAGAAGCCAATGACCAACACACCTCAGGTTGACATGGATCCAATCCGTCCTCCTCTCGTGCATGACAATCATGGGGGAGATGATGACACTGAAGACAGTGGAGATGCAACTGACCAAGGCAGTACAAGTCAAAGTGACGAGCAGCCATCAAGTGAGGATGATGAAGAAGTTGGTAATAATGATGCCAACAAAAATCCACCTGATTCACCACCAGTGCAGCAACAAAGAAGAAGTGATAGAGGTCGCATTCCTTCTTCTAAGTATCCAACACATCAATATGTGTTGATGACTGATGCAGGTGAGCCCTCATGCTATGAGGAGGCAATGTCTGATGAGAATAAGGAAGAATGGTCAGAAGCCATGCAGGATGAGATGAAATCCCTGTATGAGAATGATACTTTTGAGTTGGTGAATCTGCCAAAGGGCAAGAAAGCACTCAAGAACAAGTGGGTGTACAGAGTGAAGACTGAAGAAAACACCTCACACCCAAGGTACAAGGCCAGATTGGTTGTGAAAGGTTTCAGTCAGAAAAAGGGCATTGATTATGATGAGATATTCTCTCCGGTGGTCAAGATGTCTTCAATCCGAGTTGTGCTTGGCATGGCAGCCACCATGGACTTGGAAATTGAACAACTTGATGTGAAGACTGCATTCTTGCATGGTGACCTAGAGGAGGAGATATACATGGAGCAGCCAGAAGGATTCATGGTTGCAGGCAAGGAGCACTTAGTTTGCAAATTGAAGAAGAGCTTGTATGGCTTGAAGCAAGCTCCTCGGCAGTGGTACAAGAAGTTTGAGTCTTTTATGACTGGGCTTGGTTACCATAAAGCACAACCTGATCATTGTGTCTTTATGAAGAGGTACGCCGAGGGTGACTTCATTATTCTCTTGTTGTATGTTGATGATATGCTGATTGTTGGAAATGGCACAAAGAGGATTGCTCTCCTCAAGAAGGCATTGAGTAAATCATTTGCCATGAAGGATTTAGGACCAGCTAAGCAAATACTTGGCATGAAGATCTCCCGTGATAGATCAAAGAAGCTGCTTTGGCTCTCACAGGAAAGATACATTGAGAAGGTACTTGAAAGGTTCAATATGAAAGATGCAAAATCTGTTATCTCTCCGCTTGCAGGCCATCACAAACTGAATTCAAAACAGTGTCCTACAAGTAAGAAGGAGAAAGAAGAAATGAGGAAAGTGCCTTACCAATCTGTTGTGGGCAGTTTGATGTATGCCATGGTATGCACTAGGCCTGATATTGCCTATGCAGTTGGAGTTGTTAGCCGGTTCATGACAAATCCAGGTAAAGCTCACTGGGAAGCAGTGAAGTGGATTCTCAGGTATCTCAAGGGAACTTCTACATCTTGTTTATGCTTTGGAAGTGGTGATCCTGTATTGCAGGGCTATACAGATGCAGATTATGCAGGTGACAAGGATCGTAGGAAGTCCACATCTGGATACCTGATGACCTATGCAGGGGGAGCAGTGTCATGGCAATCAAGATTGCAGAAATGTGTTTCTACATCAACTACAGAAGCTGAGTACATAGCAGCAATTGATGCTGGCAAGGAAGTTTTGTGGATGAAGAACTTCTTGCAAGAGCTCGGCATGAAGCAAGAGAAGTATGTTCTGTT

>HvuSat07-118

CATCAAAAATCGACCCGGGACCCCAAAACAGTGCGCTATAGCCCACGAAACTGGCCCGAAACGACAAAAACAACGAGTTTTTGCGACGTCTCGATAGCCGTGCACAGGGTTCATGGAG

>HvuSat08-263

CAACACTTGCAGTTGCATATCAGGGTACAGCATCTGCAGTTACATGTCAGGTTACCAAACTTGCAGTTGCGCGTCACGTTGCAACACTTATAGTTGCGCGTTGGGGCATAACACTTGGAGTCGTATTAAATCAGTTTCTGTTGCGCGTCGGGTTACAACTCTTGAAGTTGCGTGTCGGGTTACAACACTTGCAGTTGCATGTCGAGTTATAACACTCGCAGTTGCGCGTCAAGTTAAAACACTTGCAGTTGCGCGCCGGGTTA

>HvuSat09-900

TTGTTGCTGTCAAAGTTGTCTCTGCAGCAGTATGTAGCTCCTCTTATATAGGAATCATACATCTTTAGCACCATTTTCGTACCCTCTCGATTCATCACTCTAGATTTTCACTTCATGTGAAAATGAAAACATATTTACATAGCATCTCGACATATTCCACTCGCCTTTCCTTCATTTCAATCTTCAAGAGTAGTTATCGATGCATTATATACCTCACACAATGACTTACATGTGGAAATCGGACAATTTGTAATATTATTCGTATTGTTTCGGTCCATCATGGTATATTTTCTCTTATGTGAAAATAAAAAGTTGTTTAAATAGACAAATCGACATATGCTAGTAGTCCCGCTTCATTTCTTGCTTCCGAAGTTATCTCTACAGCAATATGTAGCTCCTCCAACAACTTATATGTGGAAATGATATAATTTCAACACTTTTCTCGTAACCGTTTTATTCATTACTATAGATTTTCTCTTCCCGGGGAAGTGGAATATTTTTCAAATAGGGTAGTCGCCATATTACGACATCATTTTCTTCATTTCATAGTTTGCCACTACTCTGTGCAGCAGTATGTGGCTCCGCCTTATGTAGGAATTATACATCATTAGCATCATTTTCGCATCATCTTAATCTGTCACTTTAGATTTCCGTTCGTCTAAAACTGAAAATTATATTATATGGCATCGTCGACAGATAACAGTAGCCTTTCGACCAATTCTATATTACATGATAGTTTTTGTTGCATGATGTACCTCAAACAACAACTTACATGTAGAAATCAGACAATTCGTAATATGTTTTGTACCATTTCAATCCATCATGGTATATTTTCTCTTGTGTGAAACTGAAAAGTTGCTTAAATAGCAAAATTTACATATGCTATCAGTCCTGCTTCAT

>HvuSat10-5985

CACTTTTCAGTATTCTCGGCAATATTTTATAGGCCTCATATTTGCCCTACGATCGATATTTTGGAAATATTATGATTCTATGTAGCCATTCAACACCCACCGTAGTTGGCGGCGTGCATGGATGAACGTTATGTGTAATTTGAATTTATTTTTTGTCCGAGTGTGTCATTTTAGTTAGGACAAATAATTCATGGTGATTTCTATTCGTTTTGCAAACTTTACAAATATCGGCTCGTCTACTAATTTACTTCTTGTGCCTGCTATTTTATACTGAGCGGTGACAATCCATCATGGTCGGGCGGATTTCATCATTTTCACCATTTGATAATTTATTCGTTTAATTACATTATCTTTTTCTTCGGCCAAAACTAGTGTCAACAGATGCCCCTCGAGGTCTGGGTTCGAAGAGATATTTTGATTCGGATCCAGAGCTCGAAACTTGACAATCCATTTTATGATCGCAGTATTTTCAAGTCCACATGGACTCTTGCTTAATTTTAATATTCCCCTTTTGAAGTGTCCGACTTTTATTCAAATGTTCGCCACCTATACGTGGATGGGTAGTCTTTTTTTTCCTACACGATACATTCTTTTTAATTTCAATTTAGGCTTGGATTTTTTTAGTGCAGTTGAACTTAACACTTCGGAGTTTAAACCCATGAAGGTTGTTTCAGCTTAACATTTTTAGACCATGCTTACATTTTAGGCTCATATAGTATTGCAGCATTTTGTATGTGTTGGTTTTGGCTCTCGAAGTATTACAGCACTTATAGACATATGCTTATTCATTTGATGTTAGGAGCATTTACTCCTATACTCATACGTGTGAACTTCATTTTCTCAGTGAATATTTAATTTCAATTGATTGGCTGCACCATAGTTTATACTCTTAAGATGTTGGGCCATTATTTTATGTGCCGTAATATATACTCATAAGGGTATGGAGTATTTTTTTAAGCATAGTAACGCTTCAAAAATTTCCTATTTATCGGTGGCGGATAGTACTCGCCGTCTAGGGAAATTATCCTGTAAGCACCACTAGAGTAGACTTCTTTTACGATGTAGGGACCCTCCCATTTTGGCTCAAACTTTCCTCCCATTCGTCGTGTAACATTCATGGGATGGATGATGGTGAGCACTAGGTCTCCTTGCTTGACTGATCTTGTTTTAACGCGCTTGTTGAAAGCGCGGGACATTCTAGCCTGATGTGCTTCCAAATTATGTAGCGCCTGAAGGCGTTTACCCTCAAGTAATTCAAGCTCATCGAGGCGTAATTTTATGGCTTCTTCTTCAGAAAGTTTCTCCTGAATAGAGACTGTGAGCGAAGTAATTTGTACTTCTAATGGTAGAACCGCTTCTCCACCAAACACAAGGGCGTAAGGTGTCATTCCAGTTGGTGTACGTGTCGTGGTCCTGTAAGCCCACAAGGCCTCAAGGAGTTTCTCATGCCACTCACGCTTATTCCTTGACACGACTTTCTTTAACAATTTGCATAGGGTTTTATTAAAAGCTTCTGCTTGTCCATTAGCTGCCGGGTTATAAGCCGTAGAATAATGATAACTTATCTTAAATTTTGAACATAACTTATCAACATGTTTGTTTTTGAATGGTCCTCCGTCATCGGACATGATACGGGAAGGAACTCCGAAGCGATATATAAGTTGTGTTCTTATAAAGTGTTCCACATATTCTCCCTTATCCTCTTTTAATGGTATAAGCTCAGCCCATTTGGAGAAATAATCGATGGCAGCCAGGATAAATTTGTGGCCCTTTGAAGATGGCGGATTAATAGGTCCCACTATATCCATTCCCCACATTTCAAACGACCATACCATATTTGTGGGATGCAACGATTCATGAGGTTGATGTATGAAACTACCATGTATCTGGCACTCATGACACCTTTTTGCAAATTTCATGGCTTCGTCGAACATGGTAGGCCAATAATAACCAAGATGTCGGAGTTGATAATATAACTTTGGGCCCGATTGATGTGCCCCACACACCCCAGCGTGCACTTCATTTAAGGCTTCCAAAATTTCATTCCCCGCCAGACAACGCAGGAGGATGCCGTCGAAAGAACGCCTATACAGGGTGTCATTTATACATGTATAGTGGACTGACCTTTTTTGAACTCTAGATCTTTCTGCAGAGTCTAAAGGTAAATATCCGTAGAGCAAGTATTCCACGAAGTGGCTTCGCCAATATCCGACTTCAATCCTGTAGATGTGTGTAGTAATTGCAAGTGATGTCGTCGTCTCTTCCTCTTCGACGAGTATAGGTAGGATTCTGCATTCTTCAACACGAACGTCCATCATTTGATGTGAACTTAATGACATACTAGCAGCTAAACCTGCTAATGCATCGGCCTTTTCGTTTTGACTTCTTATAACATGCTGAATATTTAGATCAACAAACATGGACATGAGCGTTTTGGCTCGTTGGAAGTAAGGCATTAATTCCTCTTTCTCTACCATGTAAATTCCCATTAATTGGTTGATCACTAACTGAGAGTCACCATATACCGTGAGTGAATCTAACCCCATATTCAGAGCCAATTCTAGCCCAATTATCAAAGCCTCATATTCAACTGAATTATTTGAAACTCCTGACGTTAGCGTAAAGGAGTACGGGATTATTCCTTCACTTGGTGTGACAAACACAATTCCCGCACCTGCCCCGGATCGCCGACATGCGCCATCGAAGTACATTTGACATGACGAACTAGCAACCGATGTAGCGAACACATCTTCATCAGGCAAGTCATCATCTATTGGGAAAGCATCGGGTATTGGATGCGTGGCTAGGAAGTTAGCTAATGTGTGTCCCTTCATGGCCTTTTGTGGCACAAATTTTATATCAAACTGTGACAAAAACATCGCCAACTTAGCTAATATACCTGAAAGCACCATTTTGTTTAAGATATATATGAGAGGGTCTGCTCTTGACACAAGGTGTACAGTGTGTTCTAGCATGTATTAAAGGAATTTTTGCGCTGCAAACACTAATGCCAGACATATTTTGTCCATGTCCGGATAATTGCACTCAGCCCCCACAAGGGTGCGACTAAGATAATATAGGGACATTTCTTTTCCGGACTCATTATTTTGTGCCAAAAGAGCACCCAATGACATAGGCATGGCCGCTGTATATAGGATCAAAGGGCGTTCCCTAACTGGTGCACCCAGAATAGGTGGATATATTAGATGTTTTTTAATATCGAAGAATGCATTCTGGCATGCTTTATCCCACTCAAGTGTAACACCCTTTTTCATGAGACATGAGAAAGGTTTGCATCTTCCCGATAAGTTCGAGATAAACCTCCTAATATATGCTAGGTGACCTTGGAAACTTTTTAATTCGTGTAGGTTTGTGGGTGGTGGCATCTTAACAATCGCTTGTACTTTATCTGGGTCGACTTGTATGCCATTCTTTGTAACCACAAAACCGAGAAATTTCCCGAAAGAAACTCCAAATGCACATTTCATGGGATTCATTTTCAAGTTATGTTCCCTCAAACGCTCGAAAACAATTTCAAGATGTTTTATATGGTCTTCCTCACGTTTTGATTTTACCACTATGTCATCTATGTAACATTCAACGATAACATATAATAATCCATCTAAAACTATAGGCATAGCTCGTTGATATGTTGCCCCGACGTTTTTTAGTCCAAATGGCATGACTTTATAACAGAATATGCCTTTGGCGTCCTAAAAGCCATTAACTCTTCATCCTCTGGGGCCATTTTTATTTGATTATATCCGGAATACCCATTCATAAAAGAAAACATTTCATGGTGCATGGTAGCATCAACGAGTAATTCAGATATCGGTATCGGAAAATCATCCTTAGGGCACGCTTTATTTAAGTCCCTAAAATCTACACATACACGAATTTGACCATTTTTCTTTTTAACTGGAACAATATTGGAAAGCCACTTAGGATATTTAACCTCTCTAATGAATCCAGCAGCTATGAGTTTGTCAACCTCGGCTTCTATTTTGGGAAGTAGCTCGGGTCGAAATCTCCTTTGTGCTTGTTTTACGGGCCGAATATCATCTTGCACGGCAAGTCGGTGCACGACCACCCCTGGATCTAGGCCGGGCATTTCCTCGTAAGTCCAAGCAAACACATCCCTATTTTCTGAAAGGAACTTACGATATTCCTCTTTATTTTGTTTGGACAAATGAGCGCCAATAAAAATTGGTCGAGGCTCCTCAAGTGTCCCAATATTTAGTTCCTCCAAGTCATCAATAGTTGGCTGATTACCATCCTCCATTTGCGGGGATGCCTCAGTTGGCTCTATAAATTCGGTCGAGGTCATGCAAACCTCGTAGCCTAGCCCATGTCTTTTTTCTTTCAAAGGATGGCCTTTGTGTAGAGCTTCGAGTTGTTCTGGTGACAACGCCTTTTCAAATGGCCCTCGCTGTCCCCGGCCATCGCATAAAGATGGGCCACTAAGAGTGTCATAGCCCATTTTCTGCATCATATACAAAACTCGGTTATCATACAAAGCAATAGGCAATGAATCATCATCTTTATTCTGCAAGGAGATATGCACCACATTATTATTTTTATTTTCCCAATTAGGCGACTCATCTTTATCTTGTTCAATTTTATGTTGAACCAAAGGAGGCAAGATAATAGGCATTTTGGTACCTCTTTTGATGGATGATTTATTGATGACGCGAAATATTGGGTCACCTTCTTTTCTTTGGTCACTTGGTATATACTGGTAAATCTTTCGTCCGGATGAGGATTCTGCAATATTTTTCTCTACCACATTTGACTCAGGTACTACTACAGACTTTGGTTTGTATACTTTTTCAACTAGCTCAAAATAGAATTTTGCATCTGCATAGAAGGATTCAGCCACGGTGAATGGCCTATTATCTGCAAATATTCTCATTGTTTCCCCAGACTGCTCTTTATACTTGATACACTGATGAAGCGTGGATGGTACAACTTGATTTTCATGTAGCCATGGTCGGCCTAGCAGTGCATTGTATGAAGTAGCTGCATCAATCACGTGGAATTTCACGTATGTGGACAATGCTTCCAACTTCAATACCAATGAGATGGTACCCATGGCCTCCTGACCTGATTGATTAAAGCCATGAATAACCACATTTGACTGGCTCAGGTCCTTAGGTGAATACCCTATCCTTTTCAAAGTACGTAGGGGCAACAAATTTATGGCGGATCCACCATCTATCATAATGCGATTCGTTGGCAAGTCATCAATCTCACCAAACATCAGGAGAGGTCTGTTATGCTTCTTTGAGCCTAACAACATGTCTTCATCCGAGAATGTAATATTTAACACATTACTTAGTTCAGTTTCTATCTGCGAGACTTCCACTCTATAATCTTCAGGAAATGACACTGCAGTAACTAGTGCCTTACGTAAATCCGAAGAAAGAGACAAAGCATCATACACAGAAAGCATAGCTGGTATTTTCTTCAAATGCGAGATTACATCATACTTGACCGACACCTTAGAGGGCGGGTCCATTGTCTCATTCTGAGCTACATCTTTGTTTCTAGGCTCCTTTCTATGTGGAGGATGGGGGTCAACTAACTGGCGACCTGATCTAAGGTGGACCTGATCCACTTCTGGCGCATCTTCTTCGGGGAAATGAAGAGACTCGTCATCATCCTCAAACACATCGATAGTGAGAACGTTGAATTCTTCACTTGTAGGAACCTTCATATGCGTCAGAGTCCGGGCGACACAGAAGTGTACGGCACCGGGGCATCGAGACTCCGTGGCTCTTCTTCGCTCGTCCACGACATTTAGAAAGATGGGCATTTACAAAGAGGTTGAGAGGAACGGACACGAGCGCCGTCACATTTTATAATTTCTTGGCACTTTTCAGTATCCTCGGCAATATATCATATGCCTATTTTTTGCTCGTCCTCATTTTGCACGGTTAACTATATTACTTTCAATTTCTTATTTTTAAAATATATATATCAAGG

>HvuSat11-366

TTGGTTCTGAATCAATTTTGAGTAGTGTGAACTAATACAGAGATATCTGCCCCATACAGAGTCCATTTTTCCTCAACGAGTACCCGAAATACTCATGAAAACCAAACAATTCACATGAAACTTTTGTAGGAAACATTTTGAACATGTTTCTTAAGGAGTTCGTTCACAAACTGCATTGGAACTTTCTTAATGAATGCTAGACGAACGCGTTCAGAGAACATACACCAAGTCCCAGCTTGTGCCCCAGTACATCTTCAGACTATGGTCTCTCGCGTATCCCTAGACCTAATTGATGTCATATTGCGATTTTTAGGGAAAAATGAGAGGAAAAATCGGATCCAACAAGACAAGAACTGCTCTATAAAC

>HvuSat12-541

TAATATGTTTTGTACCATTTCGATCCATCATGGTATATTTTCTCTTATGTGAAAATAAAAAGTTGTTTAAATAGACAAATCGACATATGCTAGTAGTCCTGCTTCATTTCTTGCTTCCAAAGTTGTCTCTACAGCAATATGTAGCTCCTCCAAAAACTTATTTGTACAAATGATATAATTTTAGCACATTTTCATAACCTTTTCATTCATTACTGTAAATTTCCCTACCGTGTAAACTGAAACATTTTTCAAATAGGATAGTCGTCATATTACAACAACATTTTCTTCATTTCATAGTTTGCCACTACTCTGTGCAGCAGTATGTGGCTCCTCTTATATAGGAATCATACATCTTTAGCACCATTTTCGCATCATCTTAATCTGTCACTTTAGATTTTCACTTCATGTGAAAATGAAAACTAATTTATATGGCATCGTCGACAGATAACAGTAGCCTTTCGACCATTTCAATATTACAGGGTAGTTTTTGTTGCATTATGTACCTCAAACAACAACTTACATGTGGAAATCGGACAATTTG

>HvuSat13-319

CACGGATTGTCAGAAAATTCTAAAATTTGACGTGCCGCCTTGTGAAGTTCATCGTGAAGCGTGGAAAAAAATGAAAAAATTTCATGAAGGGTAGTTGCAAGATGGCGTTGTAGCACCCCTTCCGTCCATACCGACACCCATGGGTTGCACATGAAGTACATGCATGCATTCATGAAATTCACCCAACTTTTGCCGGCACCTGAGAATGCACATGCAATGATGCCACGCAAAATTTCAACGAAATCGGAGACCAAACGCACGTTGCGTCACGTCCGGCATCTGTTTGTGCCGGTTTTTGAATGGAAATTCATAAAAAAAG

>HvuSat14-2330

ATATAATATTTTTCCGTGTTCAGTTAAGTAATGTCCCTTACGGGAAACAGAATTCATAGACAATTCCCTTCGACCCGACGAGGGTCGAAGCCACTGATCGAGGGAAGGTGCCCTTCCCATAGAGCTCTAAAATTGGAAAAAAGTCAAAATTTTTTCAATTTTTTCGGAACCTTTCTCACGAAAGGAACTACTCTATCCCGAACAACGGTCGGTCGGTTTGGAAACCTCGACCTCCCTCTCTTCACACATTGTGCTCGTGGTTTTATCTGCCTCTTGTTCTTTAAGAAACAAACGGCAGAAAAACCACCAATGTGTGCTTGACACCCCCGGTAGGCCCCCTCAAGGGGGACGGACACCAACTGGAATGGAAGGTCAGTCAAAGGAATAAGTCTATAGAATTCTGTTTAGTCCCTTCCATATCCAGGTGGTGAGGAGGAGAGGAGGCACACCCTCGCCCATACGAGGGGGGAGGTAATTAGATTGTTCTGTTTCACGACGGAGCAATAAGGATATTGCAAGGTAGTGAAACATGCAATAACAAGCCTCATGCGTAAAGGGAGCCCACCTGTAAGGGTGGCCCCTTTACAACAGAACATAGGAAGTAGAGGCCCTACTTATCTTACCCCCTACGAGTCCGGTCTCCGGGGGCGCTAATTCCAGGTACCGATTCCAAAGGAAAGGTACCGATCAGAACAGAACAGAACAGAACAGAAGCAAGGTACCGATTCATTCCTGAGGTACCGATCTTGCTTTGCTATTTCCAATGATGTGATGCTTTGCTATTCAGGGGAATTCCTCCTAGCGTCGGAAACTCTATTTATTTAGGATCGGTCTTCCTCGTTCGTTCCCTCAAGTCTTCGGTCACTCCTCGGCGACCTTGTTTTGTTAACAAAAGTATTGCACTGCTAGATTATTCCAATCAAACTATATCGACTAACCTATATGAACTCACTCACTGATGAGTGAGAAAAGGAAAGAAAAGAAAAGGCTATTTCACTTAAAAAAGGAAGCCTATTTGCCCGATTTACGAGACCAGACGTATGATCCTGTACGTGCCTACCCAAAGTCCCTGTTTTTAACGCATTTGACTATGAAATCAGGCCTCCCCGGCTGTGTCATGTGACCCCAAACATGCATACCATGGAAATACAATCTTGGTATTCAGGGGAATTCCTCCTAACGTCGGAAACTTTATGTTTCCCCTAGCTTTGCACCAAGCAACTTTTCCGAATAAAACCAATATTTCCTTGCTCAAAAAAGCACTGGAAACGGTGGCTTTTACCGAGTTACCGATCCTATCTGTGGTCGAACAGCTTGATTTCCTAAAATTTAGGAAGCCCAAAAAAAAGCAAAACCAAAGGAGATACTTATTTTACGAGCCAGAACGAGGGGGGCCTCTTCGAGACCCTACCTATCGAGGACTTGTTTTTACCCCTGAAATATTTGGTACCACAAGGCCTGTTTTCTTAAAGGAATCTGCCTTCAAACGCGTTCCACAAAAAAACAAGTAGGACCCTATTGCAAGTGCCTTGCACTTTAACCGAAAAAAAGGAAAGGCACTTTTCCTATCTTTTTCCCTTAACGCGGAGCAGTCAGGTAAAGAACTGGAAACCATACTTTTTTAAAAACTGGGAAAAGGCATATTATGACCGTTGAACCATTTGATAGCGAAGAGTTGTTACAGAAGTCTCTCGGTTTTCTTGAAACTGGGTTACCATGATTTGGTTCTGCGGAACCAAAAAACAGGGACCCAGGATAAGAGAAATACCAATTTTTTTCATGATCAGGGCGCCGTGGAACTGAGCAGCCTATAGTCGAAAGGCCAGAAAATTCTATTCCGACTAAGTGCGTCACAGGAATGACCTATTCCTTTATTCATGAGCCAGATGATGGACATGAAGGGGAACATGGGCTTGTGGCCCGGACTCTAGGTATCTGAGCCTTTTCTGATGAACGATGCACCATGCGAACTGATTTATGAGGAAGTTGGAGTCAACTTGAGCGAGAAACCGCTCCAAAACCATCCCCTCGGGCAAATGGCAAACCCTACAGTTCCAGTTTTACTGAATTGGAGGGACAGTCCCGGGGAGGCAGATGTAATAAACTCACTTCATACAAGCTACAAATGACAAGAAGGCAATGGTTGAAGGATCGCCCGTGCTTAATGTTGGGTACGAGAGCGTCCTTCGGGTCCGTTTGGTACCGGACCCGCGTGAACGATACAGATACCGGCTCCATACTGGATACGCTGGGGTAGGATCATTACAATGTAATGATCTTATCTCGGTGTTGTTATGGACGGAACTGCATTATGCACCTGACCACGGAAA

>HvuSat15-1590

CTAAAGACACGACTCTTTTTTTTTTTTTTTCCCCGCAACTTCTTCATCAAAGCAACCCTGGGCCGTCATCCACGTCAACTTTAGCTAGTTGCAGAATCTCGTGCTCCTTCTTTGCCTCCTTCCGCGGATTAGGACACTCTCGAGAATAGTGTCCATAGTCCTGACAATTGTAGCAACGAACTTTAGATTTGTCGAACTTCCGGTACTTCTGCTTATCTTTCTCCTTATTGCGCGAGAACATCAACTGCTCCTCTTCTTGGTCGCGTTCCCGTCCTTTCAACGTCAACTCATAGGTCTTTAGCCGCCCGATCACCTCCATCACCGACATCTTCGTCACATCGCCCCACTGCTCGATGGTGCCAACGATCGGTAAAAACTTGTCGGGAACGGCTCGTAGTAATTTCTCCACAACCGCGATGTCTTCCATCTTTGATCCGAGAGCTCGCATCTCATTAACTAGGGAGGTTAGTTTTAACGCAAATTCACCAACCCCTTCTGACGCCTTCATGCTCATCCTCTCGAACTCCCTCCTTAGAGCTTGAACACGTGCCTTCTTGACACGATCATCTCCAGCATGCATCTCCTTGAGCGCTTGCCAAGCTTCCTTTGCCGTCTCTTTCTCCGATATAGACATGAAAACAGCATCAGACACACCTTGGGCTATGATGGCGAAAGCCTCTTGGTCCTTTGTCTCTTCGACCGCGCCTTCACCTTCCACCGCTGCCCAAACTCCACGCGCACGCATGAAGATCTTCATCTTCGCAGCCCACACCCCATAATTGTCACCATCAAGCATGGGGTACTGGATGGTCACACCACCACCACTCGCCTTCGAAGTCGACATGATCCTCTTCTTCGATTTTCCGTCGTCCGTGACACAACTTGACGGCGAAGCCTCCACACCTTGCTCTAGATACCAATTGTTGGCTTTTCGCCCTCAGGATCGAACACACGATCACCCTGCAGCTCGACTAGTCGTGTACGTGCAACTCGGCTCAACGTATACGAACTAGTTGTAGCACTCGATATCTCACCGGCGCTTACGCAAACACCGGGACCAGAACACACGCACGCGAACGCAGCAACACGAAGATGGCCAGAGGCACGTGTCGTGCCTCGTAATAATCTCTTTATTGCTTTTCTCGTTTTTCTGGTGTTACAAAACTGAGCGTATACACGCATATATATATGCCTCAACCTAGCCCTACGACTAACAAACCGACTCAACCTGGACTTTCAAATACAATACGGACACACACCTAAAAAACCTACCAGGACACGGACTACTACTCTTGCCTAAGCAAAACTTACTAAGACTAGGGCTAGCACTCCTATCCGGACACAACATGCAAGACTCCAATAATGTTTGGATTACCCAACATTCACCCCCTAATCTAAACATCGACTTGTTGACTTGGAACCAACACATCCTGGATTACCAGTCCACGCCGTCACATCTTAGCGTGCTACATGCAGACTAAACACATAGCCTCTCATGCAGGAACTCAACTTAACTGGTGCGCATCCATCTTCACGTTGGCCTTCGTCTTCTCGCCGAGACACACTAACGACTCAGACACAAACAAAAAA

>HvuSat16-483

CTCCTGCTCCCATTGCCTTGCCAAATGCAAAAAAGTTTTTTTTAAAATGGGTGTGAGACATAGCCTAATGGAGAGGAAGAAGAGACAGAGCGTACTGGCATCGTCGTCGTCCTGGTAGCGCCTCCGACACATAGTCGTGTCGAACACCTTCACGGCGAACGTGGCGTTGCCGTCGTACCTGAAGACAAGGAAGTACCCGTGCCGCAGGTCGTAGGCACGGACGAACTGCTTCCAGCCACGATCTAGGTACATGCTTCCATCGGTGTCGAACACCACCTCCACGTCCCACAGCTTGCGAAGCCCGTTGCCGGCCTGCCTCAGCTTCATGTTCTGTGGCCGATGGCCGTCCAACATCTTCACAAAATTGTCCGGCAGCACCTGCCTGCTGGAGGATTTCTCAAGTATAATCGTGAAGAACTCCATCTCTGAAAAGCAGCAGAAGAGACCAATTACTACCCTTTACAACATTGTCAGTACTCGCTG

>HvuSat17-632

TATCACTTCTGCATCTAAATGAGCACAAACACATAAGAGCGAGACAGCTACTCCAACAAGACACATACCACTAAAATTCCCATCTATTCTAATTAATCCAAATCATCCTCCCATAATGATCCTTTACTCATAACATTCTTGGGGAGGGCTAGCAAAATCATCAAAGCCTACCAACTACGATCCATGTGTACCATCTACATGCTCATATCCTGAAGCACATACAAGCCACCAAGCTCAGGTCAAAATATCACAAATCCAACTGCCCAACCCCAAGTTTATCTCCGGTCTGGAATCCAAGTAGTTATACCATCTAACTTGACGCAAACATCATTTCTATTACTCAAAGATCTTTGTTTTCTTCCTACTAATTCAATCCAAGATGATTTAGTAGCCCATCAATTTCCACAACTATGTTGATCATATTACACCCATATAAATCCAATATATCTTCGACTGATCATACTACACCCTAAAGTTCTCTAGTTTCAATCTCCAAATGAGCGAATCTAACACAAATCATAACCATGATCCAACTAAGCCAAGTATCATGGTTATCTAAGTCCAATATAAAACAAGCTACAAGTGGATGACTATAGATCCAAAAATTATATGTTATTTATCCACCAATATAA

>HvuSat18-2988

GGGCAGCAGGCTTACTAAGCGATGAGATATATTCATTGAAGACATAAGTGAGAGAGATGTAGATACACGCAAGATAGCACACTAGGGGTTGGGCCATGCCGCTGTGGTCGGGACGTTAGAGGATTCTACGTATTTTCCACAAGCATGATCACTACCTATTCTTTAAAAAGAACTGACGTTGATACTTGAGTATGACTTCAATTCTTTCGGAAAACGCACATTGATACTTGAGTATGACATGGGGCTAATATTATGAGGCATACAAGATAAAGCGATCCACTCATTGAAAAAGAATAAAACACATAATTCATTATTCCATTGTCAAATAGTACACTCATTGAAATATTGGTAGATTCATCACACTAATACATATATGAGTAAACAATATAATTCAGGCAGCCCACATAGACAACATTGTATATGGCGATCGATATGACACTCGCTCACTTTATTCAATCGATTCTAGCCTTTAATAATAAAGGCACAAAAGATAAAGCTAGCCATGTAAGGAAGGCTAGCTAGCCAACGTATGGTGCAGGAGCAACAACCACCCATGTATTAGGCACCGGGCCGACGTCGTTGTAAATGATAACGCGTTCTTGATCTTGTGGCGGTGGGGATGGTTGCTCGCCTGGAGGCAGTACTCTGGCAATTCTGATATCAGGCCTCTCCTGATGAATGATCTGAACTGCTGCATTCAGTTCAGTACCAATCAGACCCGGCCATGATTCAGGCACTGGACCAGGCATGTCTACCCTTACCACGCACTATCCCTAGCCTTCCTCCCTTTTGAAACTGTTAATTAACAACAAGCATCAATAATTGAGAAATAAATAGGTAATAAAGAGAAACAAGCATGGTGTTTCGTTTTTATAATAACAACAATATAACAATAATATGCCATAAATTCTCTGGTATTAACTGATAATGGGATAAGAAACATGCTCTAAGCATAGGAAGATGTCATAATCGAAAGAATTATCTCCTAACTAAACAAGTAATTATTCTACTAAAATATTTAATAGAAAATGCATGCAATAATAAAAAAATACATGCCAACTATTAGGGAATAAGAGGCTGTTATACCTTAGAGAACCTTAGCTTTTCTGGTGGCGATGATCTTGTGTTCAAGGTATGTGTGTGTATAAATACAAATGTATGAGTGCACTCCCATTAGCGTAGCTTACGCCATGACAATTATAATAATGCATGCATTTTGTTCACAATTAATTGATAAGAAGCATTGGCATATGCTCTACTTGTTGTCGATGATAACAATGCATTCATTTGTCTTCATTAATTAAGGAACATCAGCATATCCACTGATTGTCGAGATTAACAATGTCGATGACAAGAATCCTTGCATTTTAGTTGATTCATTAAGAAACATCATCTCATATCCCCCTTTGGTTGAGAGTAACGATGCCGATGACACAATAATGCATGCATAAGCGTGTAAGTTGAAATAGCATGTACACATTTGATCTTAGATGTATGTATGCGGAGCATCTCCACGTAGAAACCTGTGTGTTTAAAGTGCATGTTATTGCAACGTATTTTGCTCAATTTATTTTTTCCAGCAAAGATTATTATCCCATTTATACAGAACAGTCATCACTACAACTTGCTTTGGATCTGCGAGTTATATAGTGGTATCACTTTAGATTCATAAGAAATAATGCATCAATCGTGCATATTTAGAAAGAATTGAAGTCATACTGAAGTATCAATGTCAGTTCTTTTTAAAGAAATGGTAGTGACCAGTCTTGAGGAAAATACGTAGAATCCTTTGATGTCCTGACCGTAGCTGCATGTCCCAACCCCTTGGGTGCTATCTTGCCTATAACTACATCTCTCTCACCTATGTCCTCAATGGATATCTATCATCACTTAGTAAGCCCGCTGCCTCTGCCCATAAAAAATACTCCACGAAAAGCAAAAGGAAATTTGACAGACGTACATCACAATTCCTCCTTTGATCATCACACAACTACATATAGTAGAGTAATTTACGACAACAATTGTTCAACTTAAGAGGTCAACTTCACAATTATTGGTAGCACCAGATTACAATCTTACAAGACAGATCTAGAAAGATTGCACCTTTGCTGAATCTAGACCAATAATGATGCACCATTGCACTATTGGAAGCATCCGGAACAGATAATGGACATTTGAGGAGGGAACGATACATTTATCAAATTCAACCGCTACGGTGCTAATGACGTTATTAGCATGCTACACGGCGCTATAACATCAACCAACCATCATCCGGTGGTTTCTATTTTAAGTCCGTGAGCAAACGAGCAGAATGTGCCATAGAGAGTCCAGGTGCTTTCTGCTCATCCAGAATGCCCTTTCCCTTCACTAGATCATATTCATGGTTTTGTGAGATGTTAAGAATCGAGGAAACACACTTGCAAAGAAACATTCTGGATACATCAATAGGTGGTGCAGCCTTCCCATGAACAATCTCATTACGTACGTACCATATTCTCCAAAACAACATTAGTATATTGAGCCTCATAATCTGTGTCATGTCACAGTAAATATGGAAAATCATTTCCGGTCTTGTGTTCACAAGTGTGTGTAAACTGATGGGTTGTTCCGTGATTACACTACAAAGTACAGAGGGCAGTATATATTGGGCACCGGGTGCAACCGCGACAGGAACGTGGGGGCAGATCGTAAAGGTGCAGGAGCGGGCCAAGTCCACGTTCTAACACCTCCCGTGGTGTCAGGGGCGACATCGGCGACGTTGAGACTAGAGCGAATGTCGAGGAAGGCTGTTGTTGGAAGCCCTTTGGTGAAGCGTGTGCAAATTGTGCCGAAGTAGGGACATGCAGAACAAGAACCTCACCCAAAGCTACCTTCTCCCTAACAAAATGAATGTCGATCTCTATGTGCTTTGTTCTACGATCTCTATGACATATTTCCTTTAGCTTTTCGTGGTCTATTATTTTTCTTCTTTTCTTTTTTGATGGGGAG
