# Supplementary material for: Highly divergent satellitomes of two barley species of agronomic importance, Hordeum chilense and H. vulgare
Source: Plant Mol Biol. 2024 Oct 2;114(5):108. doi: 10.1007/s11103-024-01501-5 (PMC11447152; doi:10.1007/s11103-024-01501-5)
Supplement: Supplementary file 5 — Supplementary file5 (DOCX 21 KB) [file 11103_2024_1501_MOESM5_ESM.docx]

**SUPPLEMENTARY INFORMATION**

Figure S1. The sequence for each satDNA family of *Hordeum chilense* (H1) in fasta format.

Figure S2. The sequence for each satDNA family of *Hordeum chilense* (H7) in fasta format.

Figure S3. The sequence for each satDNA family of *Hordeum vulgare* (H106) in fasta format.

Figure S4. HvuSat02-444 satellite Terminal Inverted Repeats (TIR) alignment. TTCs = terminal repeat composed of intact and degenerate TTC repeats. The sequence depicted is the complementary reverse to be aligned with GAAs = terminal repeat composed of intact and degenerate GAA repeats.

Figure S5. Curve profiles in the Repeat Landscapes (RL) of HvuSat02-444 satDNA based on the relative abundance (Y-axis) and divergence (X-axis) of the different parts composing the repeat unit: the interstitial segment (non GAA) and the Terminal Inverted Repeats, TIR (GAA). satDNA evolution would be mainly marked by amplification and homogenization processes (both decreasing divergence) and point mutations (increasing divergence). Therefore, the profiles of repeat landscapes would inform that peaks at lower divergence values are the product of recent amplification or homogenization events, whereas those at higher divergence values are probably older variants degenerated by the accumulation of mutations. Following this argument, the graphs show that the two parts composing this satellite existed in the common ancestor of both species but, unlike in *H. chilense*, in *H. vulgare* the non GAA part has had two major amplification peaks, an ancestral one coincident with the modest amplification peaks occurring to the non GAA part in *H. chilense* and the GAA part in both species, and a more recent one. The latter has only occurred in *H. vulgare* and coincides with an also remarkable amplification peak of the non GAA part.

Figure S6. Chromosome identification and orientation from the species analysis in this work *H. vulgare* (H106) and *H. chilense* (H1/H7) were confirmed by reprobing of the chromosome spreads with the GAA probe (panels a’-c’). DNA was counterstained with DAPI (blue). SatDNAs were indistinctly labelled in red or green. a) HvuSat01-338, b) HchH1Sat04-118 and c) HchH7Sat16-503. Scale bar= 10 µm.

Table S1. Primer designed in this study to amplify each satDNA family in *Hordeum chilense* in both ecotypes (H1; H7) and *Hordeum vulgare* (H106) studied in this work.

Table S2. BLAST results with the sequences of the satDNAs identified in the different species analysed in this work. The table contains the description, the scientific name of the species where it has been described, percentage identity (%), accession (NCBI) and bibliography. Sheets are ordered by satDNA and species (*H. chilense*-H1; *H. chilense*-H7; *H. vulgare*-H106).

Table S3. Homology of satDNAs with transposable elements. Different columns indicate the following data: Satellite Group (GR), complexity adjusted (SW score); percentage of substitutions in matching region compared to the consensus (Div); percentage of bases opposite a gap in the query sequence (deleted bp); percentage of bases opposite a gap in the repeat consensus (inserted bp); position in query (begin-end); strand (transcript strand (+); complementary strand (C); matching repeat; repeat class/family; coverage (%) and genome distribution (FISH signal).

Table S4. BLAST result of each satDNAs to *Hordeum vulgare* subsp*.* vulgare (MorexV3). Hits were ordered from the short to the long arm end for each chromosome. The alignment length (corresponding to the single repeat unit size), alignment start and end sites and the relative position of the alignment within the chromosome were displayed for all the satDNAs.

Table_S5. Copy number of each *H. vulgare*-H106 satDNA in the genome of the species *Hordeum vulgare* subsp*.* vulgare (MorexV3: https://0-www-ncbi-nlm-nih-gov.brum.beds.ac.uk/datasets/genome/GCF_904849725.1/). Genome size: ~5 Gb (Doležel et al. 2018).

Table S6. BLAST results of *H. chilense* (H1/H7) against the reference genome of *H. vulgare* (*Hordeum vulgare* subsp. vulgare (MorexV3: https://0-www-ncbi-nlm-nih-gov.brum.beds.ac.uk/datasets/genome/GCF_904849725.1/).
