# Supplementary figures and images for: Highly divergent satellitomes of two barley species of agronomic importance, Hordeum chilense and H. vulgare
Source: Plant Mol Biol. 2024 Oct 2;114(5):108. doi: 10.1007/s11103-024-01501-5 (PMC11447152; doi:10.1007/s11103-024-01501-5)

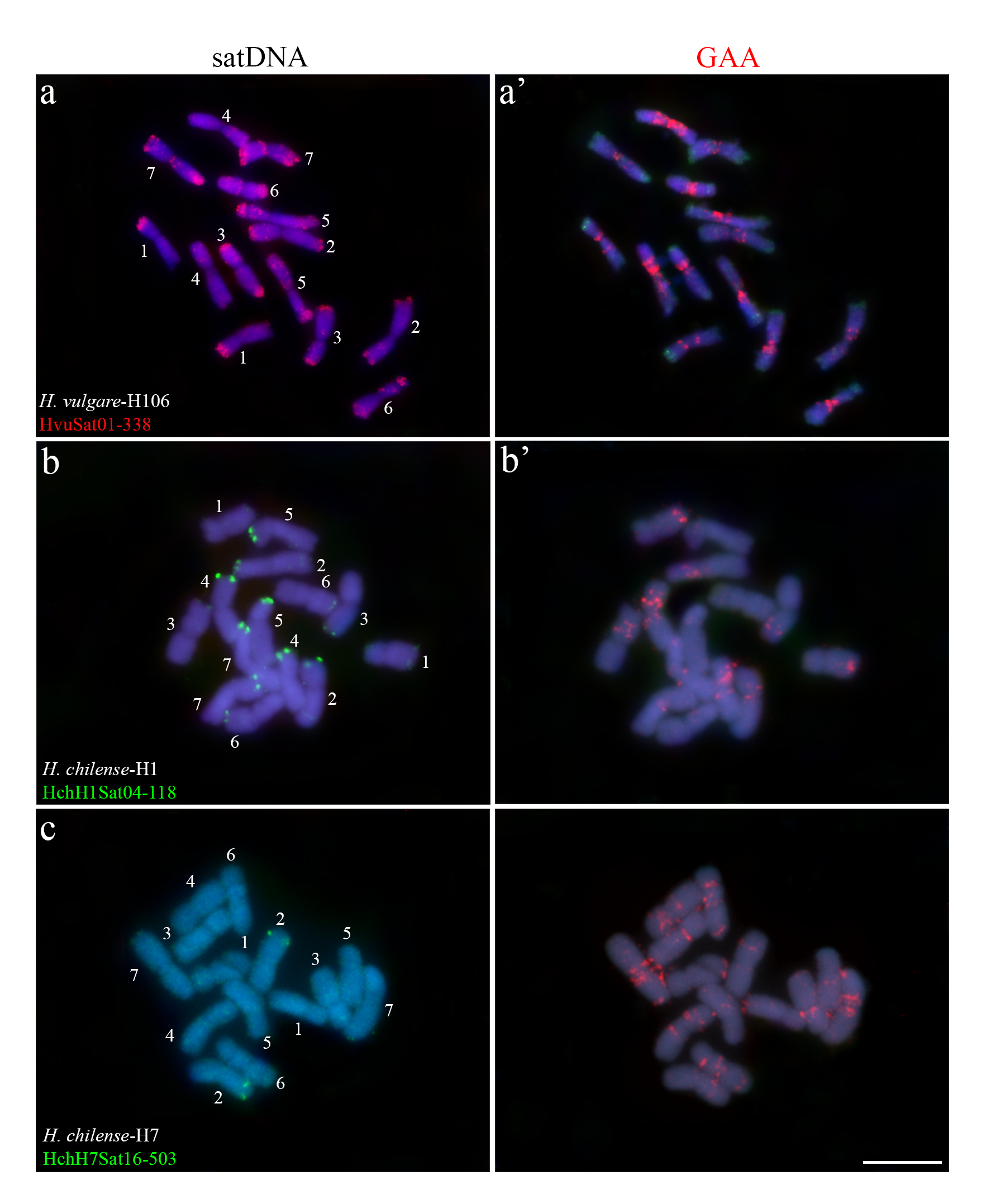

Supplement: Supplementary file 11 — Supplementary file11 (TIF 11115 KB) [file 11103_2024_1501_MOESM11_ESM.tif]

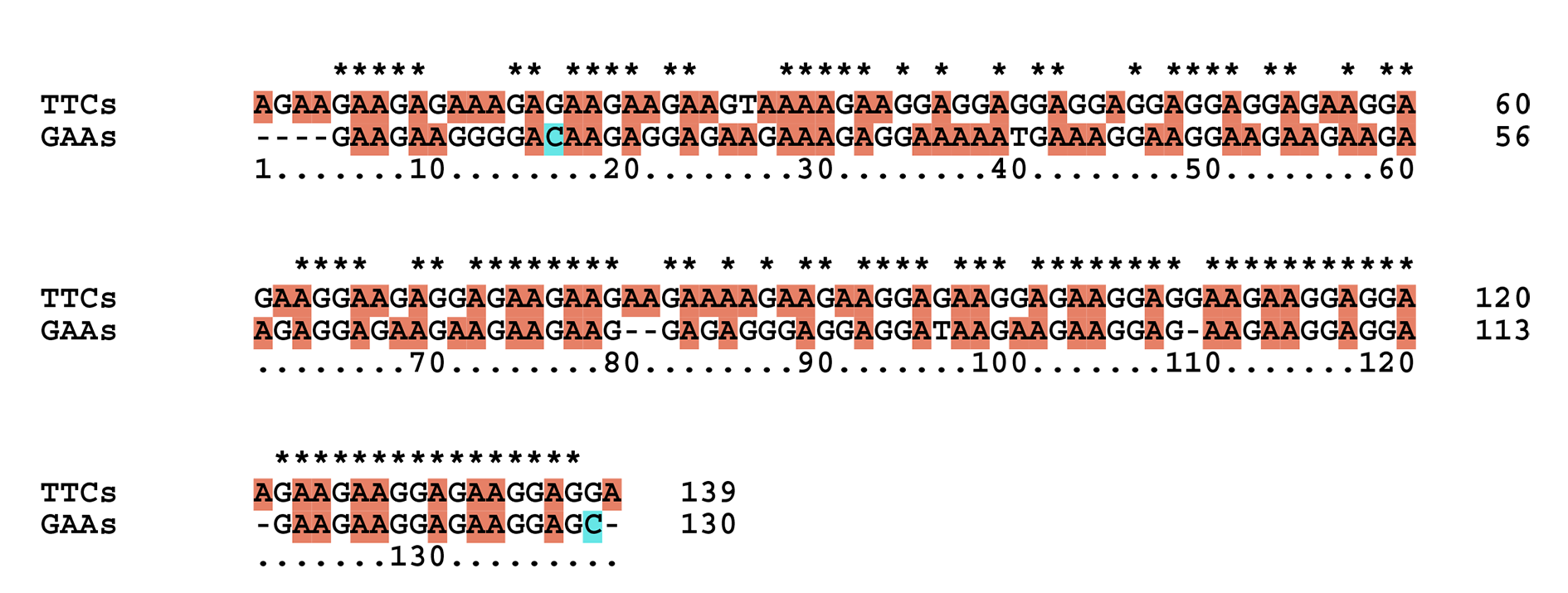

Supplement: Supplementary file 12 — Supplementary file12 (TIF 4723 KB) [file 11103_2024_1501_MOESM12_ESM.tif]
